# ﻿The Hydradephaga (Coleoptera, Dytiscidae, Gyrinidae, Haliplidae, Noteridae) of the Iberá wetlands, the second largest wetland area of South America

**DOI:** 10.3897/zookeys.1259.164084

**Published:** 2025-11-11

**Authors:** Matías R. Urcola, Juan I. Urcola, Mariano C. Michat, Patricia L. M. Torres

**Affiliations:** 1 Universidad de Buenos Aires, Facultad de Ciencias Exactas y Naturales, Departamento Biodiversidad y Biología Experimental, Instituto de Biodiversidad y Biología Experimental y Aplicada (IBBEA), CONICET-UBA, Laboratorio de Entomología. Intendente Güiraldes 2160, Ciudad Universitaria, C1428EGA, Ciudad Autónoma de Buenos Aires, Argentina Universidad de Buenos Aires Buenos Aires Argentina

**Keywords:** Aquatic beetles, biodiversity hotspot, Natural Reserve, Neotropical Region, new records, Ramsar site

## Abstract

This study presents the first inventory of Hydradephaga beetles from the Iberá wetlands, a natural reserve located in the province of Corrientes, Argentina. A total of 80 taxa were recognised, of which 62 are identified at the species level and 18 at the genus level. Of the four Hydradephaga families present in Argentina, Dytiscidae is the richest in terms of the number of genera and species (25 genera, 43 species), followed by Noteridae (6 genera, 32 species), Gyrinidae (2 genera, 3 species), and Haliplidae (1 genus, 2 species). The following five species are recorded for the first time in Argentina: Copelatus cf. inornatus Sharp, 1882; Bidessodes cf. evanidus Young, 1986; *Neobidessus trilineatus* (Zimmermann, 1925); *Haliplus nieseri* van Vondel & Spangler, 2008; and Suphisellus cf. pereirai Guignot, 1958. Additionally, eight species are reported for the first time in the province of Corrientes: *Meridiorhantus orbignyi* (Balke, 1992); Celina cf. parallela (Babington, 1842); Laccophilus cf. obliquatus Régimbart, 1889; Laccophilus cf. paraguensis Régimbart, 1903; *Haliplus ornatipennis* Zimmermann, 1921; *Hydrocanthus paraguayensis* Zimmermann, 1928; *Mesonoterus crassicornis* (Régimbart, 1889); and Suphisellus cf. rufipes (Sharp, 1882). The high diversity of Hydradephaga beetles recorded highlights the ecological significance of this protected area.

## ﻿Introduction

**Figure 1.**
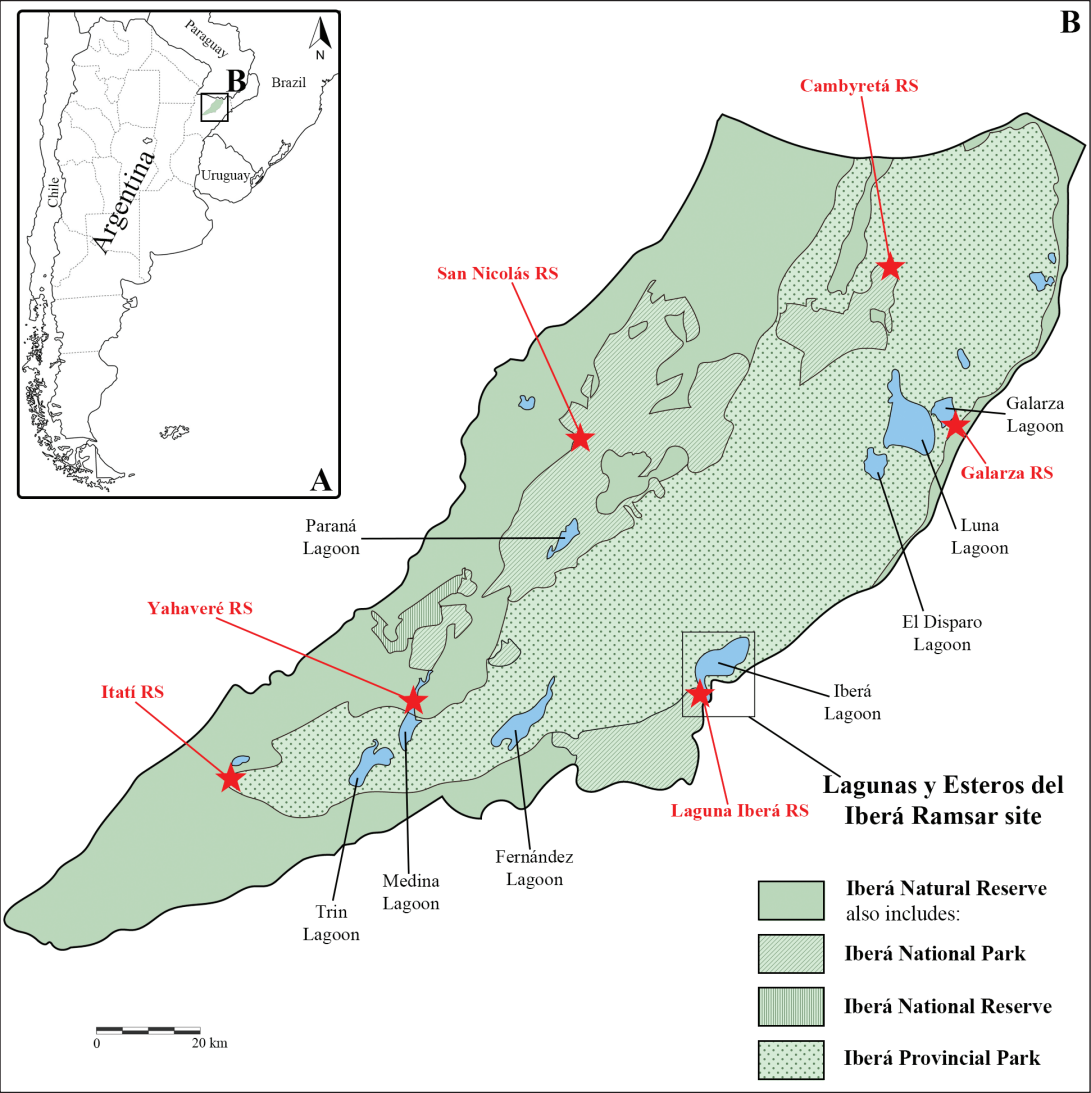
Study area. A. The area corresponding to the Iberá wetlands, located in the province of Corrientes, Argentina, is shaded in green; B. Detail of the squared area in A.

The Iberá wetlands are in a central depression in Corrientes Province, Argentina. This depression extends in a northeast to southwest direction and is bordered to the east and west by higher terrain along the margins of the Uruguay and Paraná rivers, respectively. Its area covers approximately 13,000 km^2^, representing one of the largest wetlands in South America. The Iberá macrosystem is characterized by a complex of lentic and lotic environments (Neiff 2003), including extensive areas of standing water (e.g., lagoons, marshes, ponds) and flowing water courses (e.g., rivers, streams, channels). Almost all the territory is protected within the Iberá Natural Reserve, which includes the Iberá Provincial Park (6,000 km^2^) and the Iberá National Park (1,580 km^2^) (Fig. 1).

In January 2002, an area of 245.50 km^2^, including Laguna Iberá and its surroundings, was designated as a Ramsar site by the Convention on Wetlands due to its significant ecological value. This designation was based on several reasons, including: the fact that the wetland supports a community of vulnerable and endangered species (Giraudo et al. 2006); its role as an important breeding and nursery area for various fish species (Bonetto et al. 1981; Almirón et al. 2003); and its key importance in natural freshwater filtration processes, as well as nutrient and sediment regulation (Neiff 2003).

From a biogeographical perspective, the Iberá wetlands belong to the eponymous Province (i.e., Iberá wetlands) of the Paranaense Domain, Chaco Subregion, Neotropical Region (Arana et al. 2021). This province is characterized by the absence of biogeographic barriers and the presence of river corridors, factors that facilitate connectivity with neighbouring biogeographic provinces. This connectivity has a significant impact on the region’s biodiversity, with numerous studies documenting the high biodiversity of this province (Bar et al. 2005; Ingaramo et al. 2012; Fontana 2017; Rubio et al. 2018). In particular, it promotes species exchange between adjacent areas, resulting in a high number of shared species among provinces. However, this interconnection also contributes to a low level of endemism in both flora and fauna, as species have greater opportunities for dispersion and establishment (Muzón et al. 2008).

Despite its status as a biodiversity hotspot, the Iberá wetlands present significant gaps in knowledge regarding its flora and fauna. This region, recognized for its ecological richness, remains inadequately explored in terms of detailed inventories and systematic studies, particularly concerning insects. Existing studies are limited to a few specific taxonomic groups (e.g., Diptera: Coscarón 2003a; Oscherov et al. 2007; Dufek et al. 2016; Heteroptera: Coscarón 2003b; Estévez et al. 2003; Mazzucconi et al. 2022; Hymenoptera: Arbino and Godoy 2003; Torales et al. 2003; Odonata: Muzón et al. 2008; del Palacio et al. 2022), providing fragmentary information. Since insects are the most diverse group on the planet, understanding their diversity and ecological functions in the Iberá wetlands is crucial for a comprehensive assessment of these ecosystems. In particular, aquatic beetle fauna in the province of Corrientes has received limited attention, with some studies in specific areas (Gómez Lutz et al. 2012, 2015; Torres et al. 2012; Gómez Lutz and Kehr 2017), and no specific inventories for the Iberá wetlands, despite the region’s extensive water-covered areas. Moreover, although it is considered a relatively pristine and protected area, the Esteros del Iberá faces increasing anthropogenic pressures such as agricultural and livestock expansion and infrastructure development (e.g., dam construction, channels, and road networks), which threaten its ecological integrity (Neiff 2004; Acerbi 2006; Neiff and Poi de Neiff 2006). This deficit in entomological research underscores the urgent need for more comprehensive studies to document and understand insect diversity in these ecosystems, which is essential for their conservation and effective management.

The present study focuses on the aquatic beetles of the suborder Adephaga, traditionally grouped under the term “Hydradephaga”. This grouping includes families such as Dytiscidae (predaceous diving beetles), Gyrinidae (whirligig beetles), Haliplidae (crawling water beetles), and Noteridae (burrowing water beetles), which are ecologically crucial components of freshwater environments. It is important to note that “Hydradephaga” is used here as a convenient ecological designation for these aquatic lineages, rather than implying a monophyletic clade. Phylogenetic studies consistently show that Hydradephaga is paraphyletic, with Gyrinidae being sister to the clade comprising Geadephaga (terrestrial adephagans) and the remaining aquatic families (Haliplidae + Dytiscoidea) (e.g., Cardenas et al. 2025).

This contribution aims to provide the first inventory of Hydradephaga beetles from the Iberá wetlands, based on abundant material collected between 1997 and 2022.

## ﻿Material and methods

### ﻿Study area

The Iberá wetlands are characterized by a humid subtropical climate, with mean summer temperatures ranging between 25 and 27 °C and mean winter temperatures ranging between 14 and 17 °C. Precipitation in the area is around 1500 mm annually (Montroull et al. 2013).

**Figure 2.**
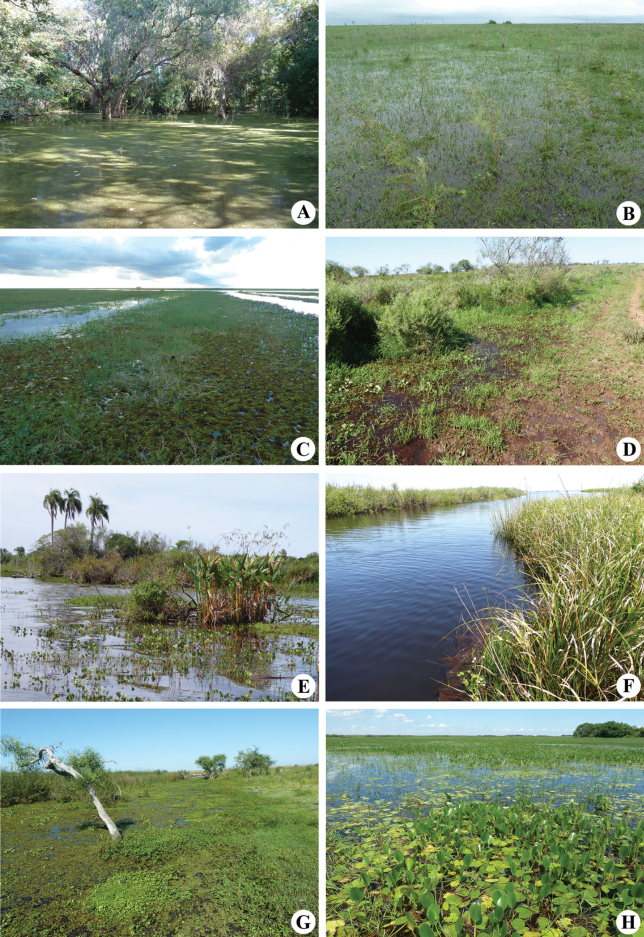
Some of the sampling sites in the Iberá wetlands. A. Marshland in Hermanos Fleita field, Galarza RS; B. Flood-prone areas in Potrero Becasina, Itatí RS; C. Artificial channel that connects the place of arrival for boats with the Corriente River, Itatí RS; D. Pond located along Route 40, Laguna Iberá RS; E. Paso Claro in Iberá Lagoon, Laguna Iberá RS; F. Miriñay channel, Laguna Iberá RS; G. Marshland next to the barbecue area of the ranger station, San Nicolás RS; H. Carambola Stream, San Nicolás RS.

Sampling sites included lentic and lotic environments around the following ranger stations (RS) (Figs 1, 2): Cambyretá (27°52'07"S, 56°52'49"W), Department of Ituzaingó, 16–19 November 2018; Galarza (28°05'50"S, 56°41'50"W), Department of Santo Tomé, 16–19 April 2013; Itatí (28°44'34"S, 58°07'36"W), Department of Mercedes, 25–27 September 2003, and 28–31 March 2014; Laguna Iberá (28°32'46"S, 57°11'45"W), Department of Mercedes, 6–11 August 1997, 24–26 March 2002, 1–3 December 2012, 14–17 February 2018, and 5–15 November 2019; San Nicolás (28°07'41"S, 57°26'04"W), Department of San Miguel, 13–15 December 2013, and 4–10 November 2022; and Yahaveré (28°32'24"S, 57°44'50"W), Department of Concepción, 5–8 November 2015.

### ﻿Sampling procedures

For the collection of aquatic beetles studied here, the techniques employed were the use of aquatic nets (round bag, 15–30 cm ring diameter, 0.5–1.0 mm mesh) and light traps. When collecting in aquatic environments, the net was moved from the bottom to the surface in a sweeping motion, ensuring thorough coverage of both the water column and the submerged and emergent vegetation. The contents of the net were transferred to a white tray containing clean water. Visible specimens were handpicked with the aid of a pipette or entomological forceps and preserved in small vials containing 96% ethanol. In some cases, the net contents were also preserved in 96% ethanol and examined later in the laboratory. The light trap consisted of two rectangular pieces of white cloth, one placed vertically with a 250-watt mercury lamp at the center of the upper edge, and the other piece laid on the ground below the vertical cloth. These were set up from dusk until after midnight, and the specimens of interest were manually collected with entomological forceps and preserved in 96% ethanol.

### ﻿Laboratory work

The collected specimens were identified to the lowest possible taxonomic level using available keys and literature. In cases where identification to the species level was possible, one specimen was selected for photography and illustration. Photographs of the dorsal and ventral habitus of each selected specimen were taken using a Nikon D800e digital camera equipped with Nikon AFS VR Micro-NIKKOR 105 mm f/2.8G IF-ED, Raynox DCR-250, and MSN-202 lenses (Tokyo, Japan). The images were stacked using Helicon Focus 6.7.1 Pro software (Kharkov, Ukraine) and digitally edited with Adobe Photoshop CC 2019 software.

All studied material (see Suppl. material 1) is held in the collection of the Laboratory of Entomology, Facultad de Ciencias Exactas y Naturales, Universidad de Buenos Aires, Argentina (LEBA). Specimens recorded only with collection site information were georeferenced using Google Earth, providing latitude and longitude coordinates, shown in brackets as: “[…]”.

Under each species name, the data corresponding to the original description are included, specifying the original name, the author, the year of publication, and the page where the description is found in the original work (see Suppl. material 1).

### ﻿Species distribution

The geographic distributions of the studied species were determined from publications reporting examined material from specific localities. Details of these distributions and their corresponding references are available in Suppl. material 1.

## ﻿Results

**Figure 3.**
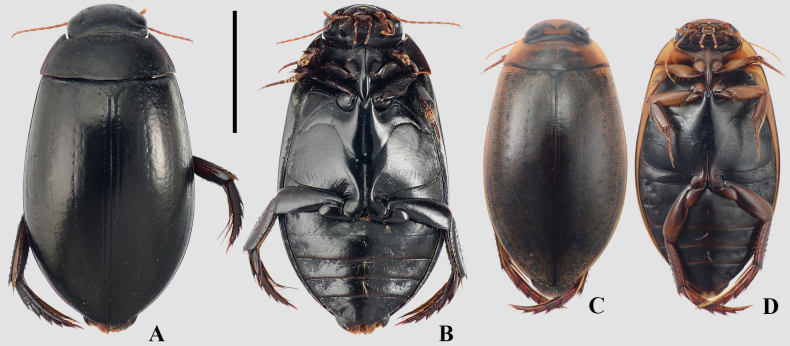
A, B. *Meridiorhantus orbignyi*; C, D. *Rhantus signatus signatus*; A, C. Dorsal habitus; B, D. Ventral habitus. Scale bar: 5 mm.

**Figure 4.**
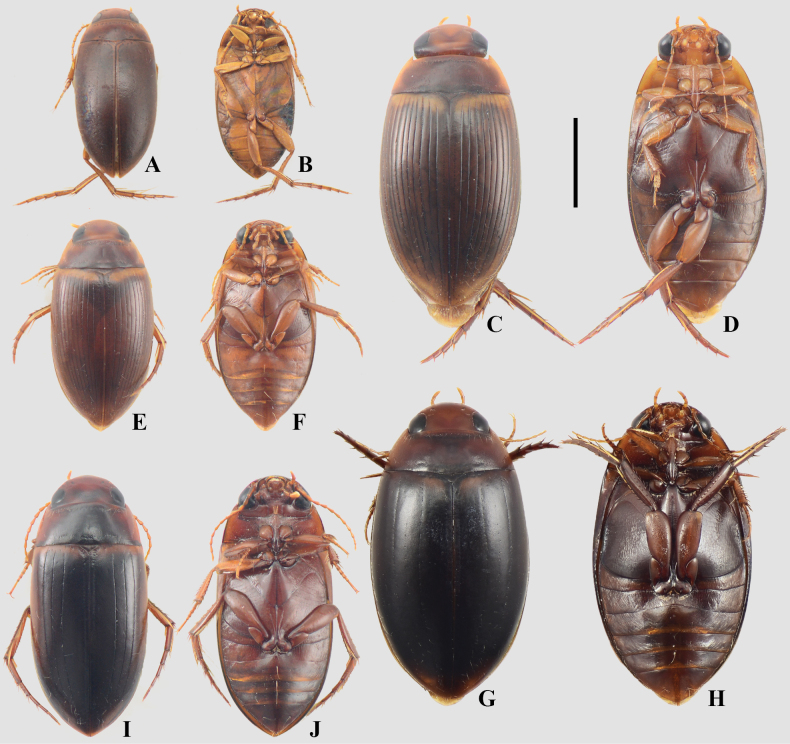
A, B. *Agaporomorphus mecolobus*; C, D. *Copelatus alternatus*; E, F. *Copelatus caelatipennis*; G, H. Copelatus cf. inornatus; I, J. *Copelatus longicornis*; A, C, E, G, I. Dorsal habitus; B, D, F, H, J. Ventral habitus. Scale bar: 2 mm.

**Figure 5.**
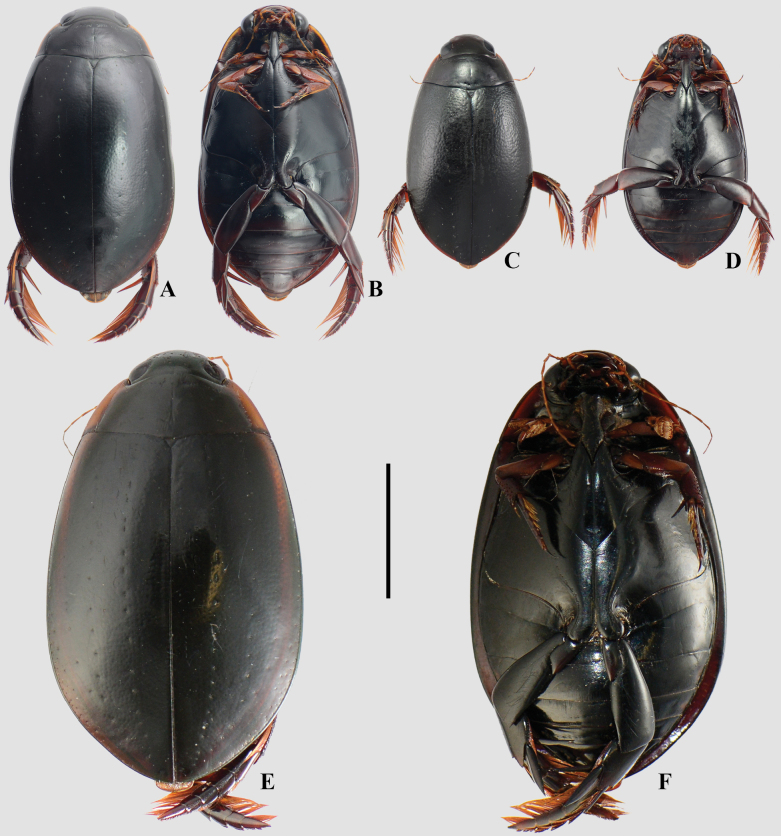
A, B. *Metaxydytes carcharias*; C, D. *Metaxydytes laevigatus*; E, F. *Trifurcitus robustus*; A, C, E. Dorsal habitus; B, D, F. Ventral habitus. Scale bar: 10 mm.

**Figure 6.**
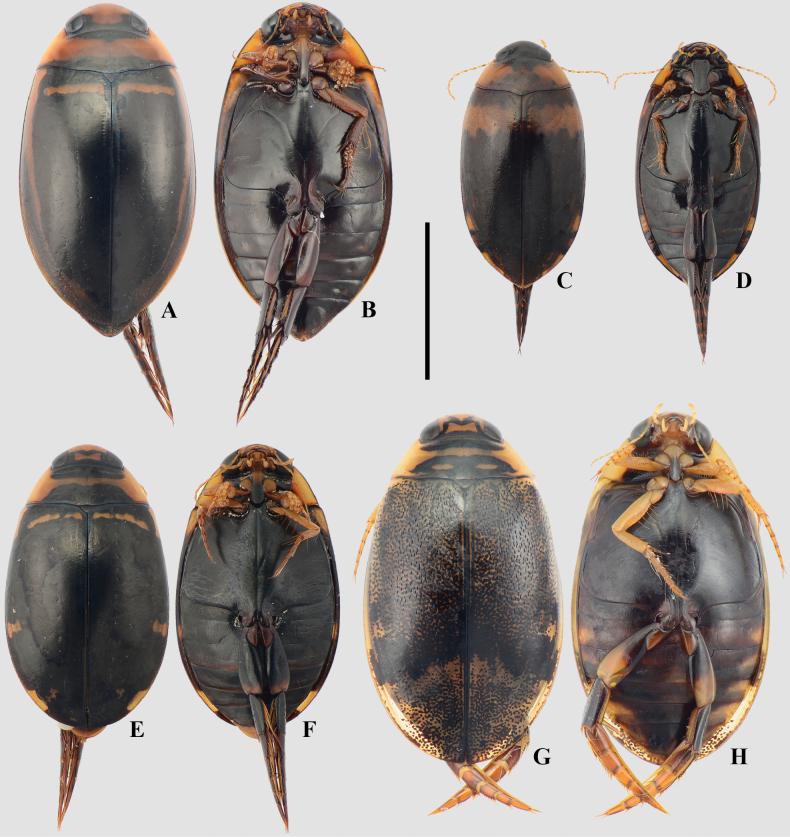
A, B. *Hydaticus xanthomelas*; C, D. *Notaticus fasciatus*; E, F. *Thermonectus nobilis*; G, H. *Thermonectus succinctus*; A, C, E, G. Dorsal habitus; B, D, F, H. Ventral habitus. Scale bar: 5 mm.

**Figure 7.**
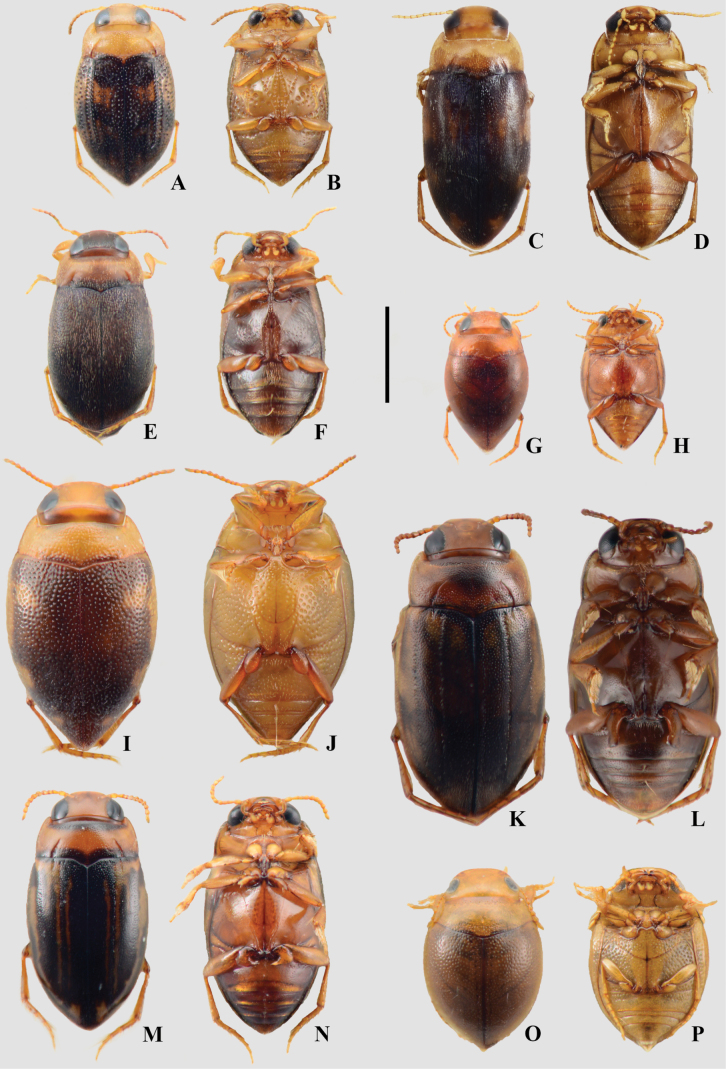
A, B. *Anodocheilus maculatus*; C, D. Bidessodes cf. evanidus; E, F. *Bidessonotus obtusatus*; G, H. *Brachyvatus acuminatus*; I, J. *Hemibidessus conicus*; K, L. *Neobidessus grandis*; M, N. *Neobidessus trilineatus*; O, P. *Hydrovatus turbinatus*; A, C, E, G, I, K, M, O. Dorsal habitus; B, D, F, H, J, L, N, P. Ventral habitus. Scale bar: 1 mm.

**Figure 8.**
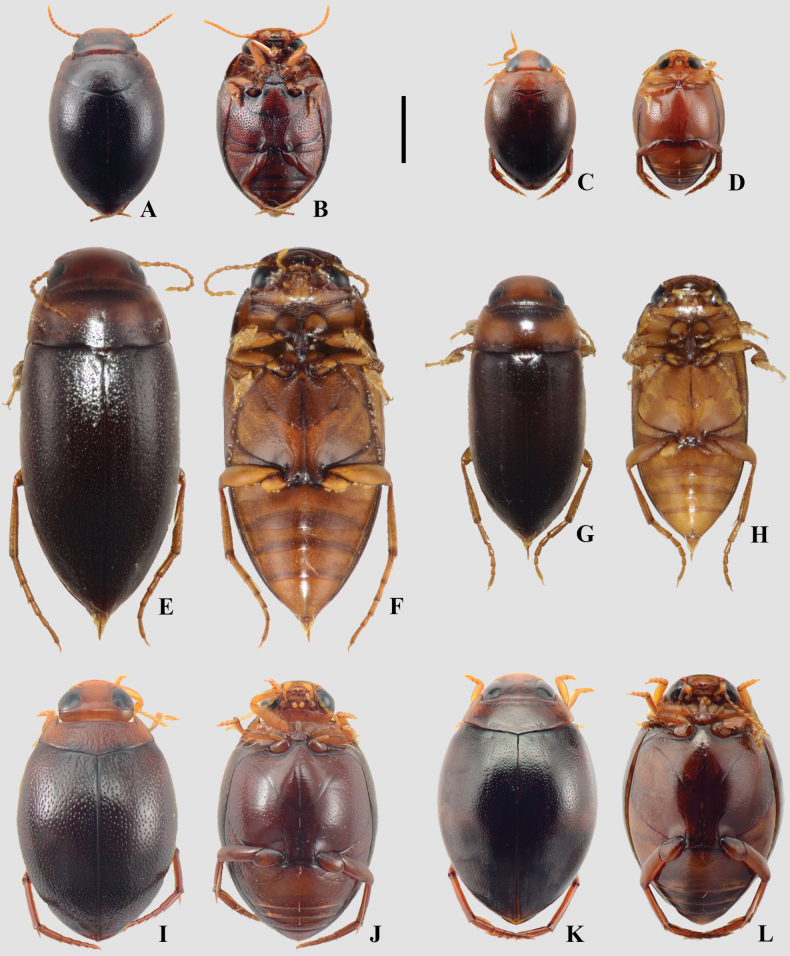
A, B. *Queda hydrovatoides*; C, D. *Desmopachria concolor*; E, F. Celina cf. latipes; G, H. Celina cf. parallela; I, J. *Pachydrus globosus*; K, L. *Pachydrus obesus*; A, C, E, G, I, K. Dorsal habitus; B, D, F, H, J, L. Ventral habitus. Scale bar: 1 mm.

**Figure 9.**
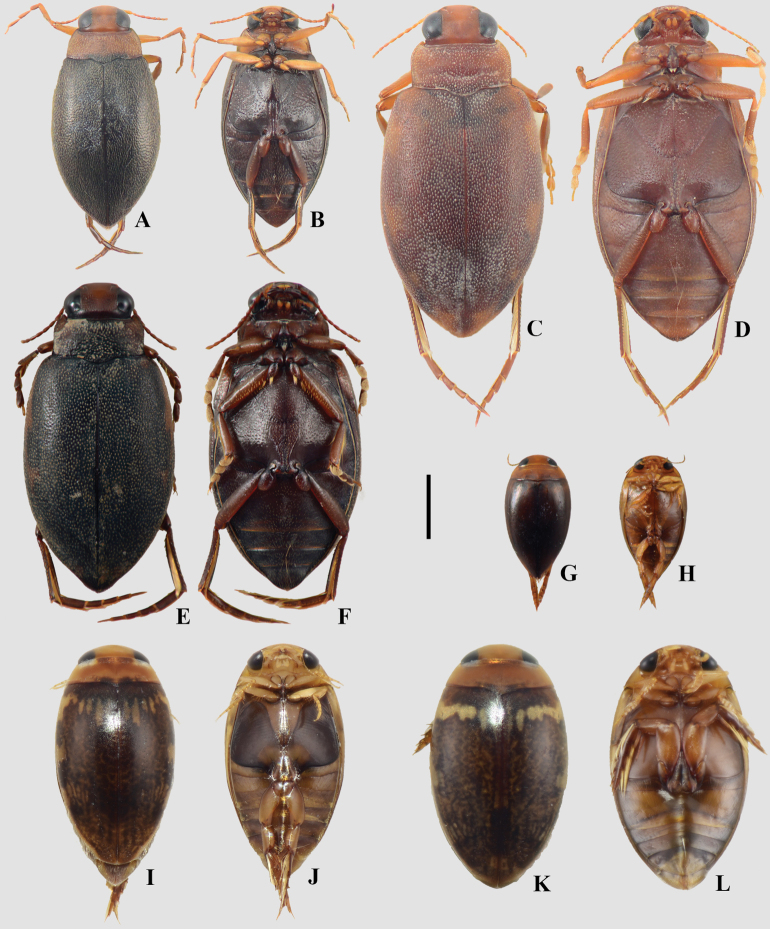
A, B. *Derovatellus lentus*; C, D. *Vatellus haagi*; E, F. *Vatellus wheeleri*; G, H. *Laccomimus alvarengi*; I, J. *Laccophilus obliquatus*; K, L. *Laccophilus paraguensis*; A, C, E, G, I, K. Dorsal habitus; B, D, F, H, J, L. Ventral habitus. Scale bar: 1 mm.

**Figure 10.**
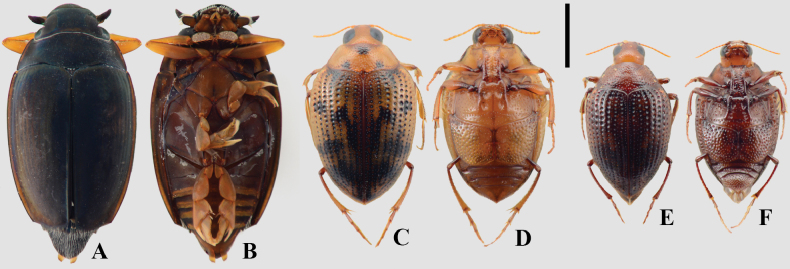
A, B. *Gyrinus violaceus*; C, D. *Haliplus nieseri*; E, F. *Haliplus ornatipennis*; A, C, E. Dorsal habitus; B, D, F. Ventral habitus. Scale bar: 1 mm.

**Figure 11.**
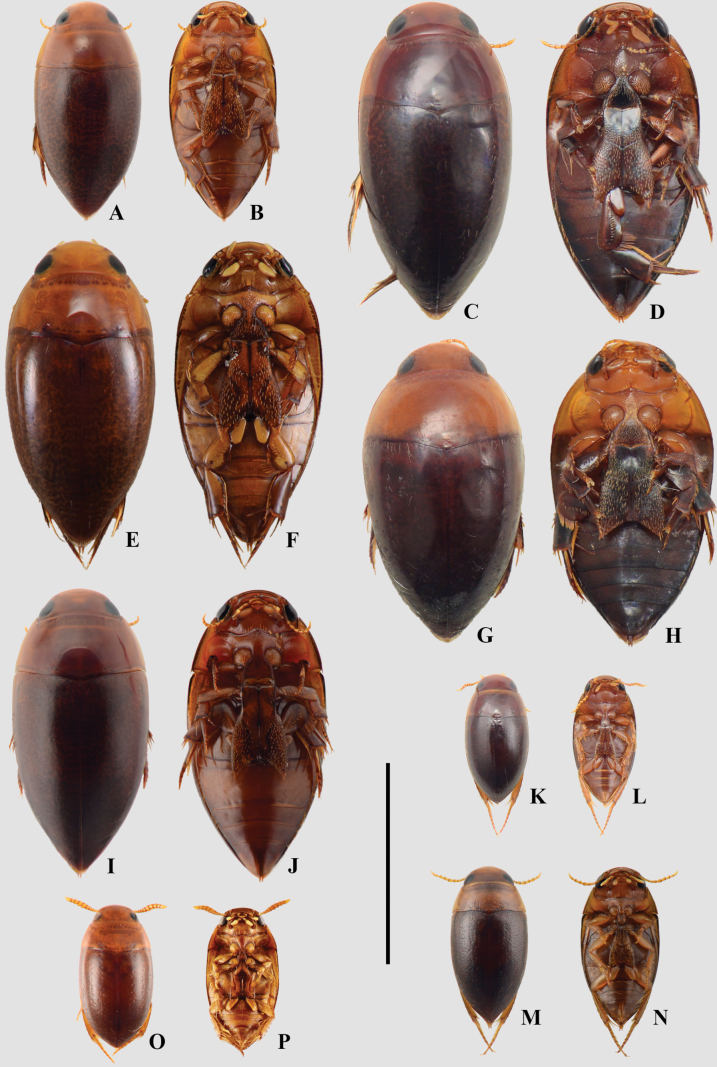
A, B. *Hydrocanthus debilis*; C, D. *Hydrocanthus levigatus*; E, F. *Hydrocanthus paraguayensis*; G, H. *Hydrocanthus sharpi*; I, J. *Hydrocanthus socius*; K, L. *Mesonoterus crassicornis*; M, N. *Mesonoterus laevicollis*; O, P. *Prionohydrus cambyreta*; A, C, E, G, I, K, M, O. Dorsal habitus; B, D, F, H, J, L, N, P. Ventral habitus. Scale bar: 3 mm.

**Figure 12.**
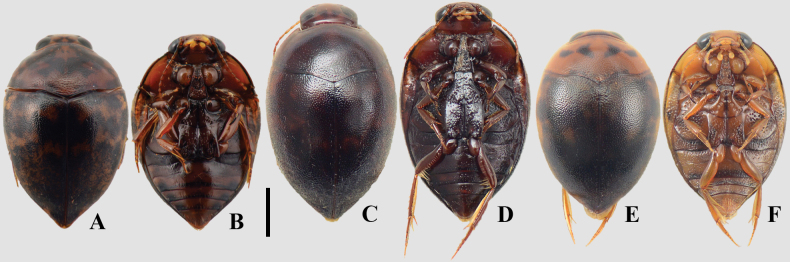
A, B. *Suphis cimicoides*; C, D. *Suphis freudei*; E, F. *Suphis notaticollis*; A, C, E. Dorsal habitus; B, D, F. Ventral habitus. Scale bar: 1 mm.

**Figure 13.**
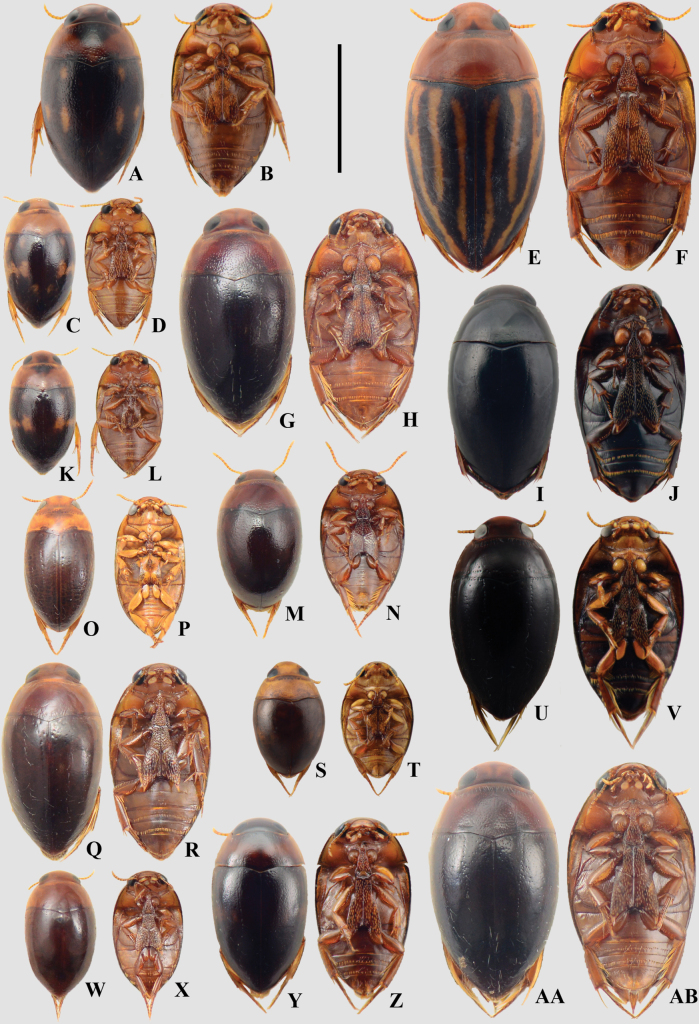
A, B. *Suphisellus balzani*; C, D. *Suphisellus flavopictus*; E, F. *Suphisellus grammicus*; G, H. *Suphisellus grossus*; I, J. Suphisellus cf. nigrinus; K, L. Suphisellus cf. pereirai; M, N. *Suphisellus pinguiculus*; O, P. *Suphisellus punctipennis*; Q, R. *Suphisellus remator*; S, T. *Suphisellus rotundatus*; U, V. Suphisellus cf. rufipes; W, X. *Suphisellus rufulus*; Y, Z. *Suphisellus sexnotatus*; AA, AB. *Suphisellus variicollis*; A, C, E, G, I, K, M, O, Q, S, U, W, Y, AA. Dorsal habitus; B, D, F, H, J, L, N, P, R, T, V, X, Z, AB. Ventral habitus. Scale bar: 2 mm.

A total of 80 Hydradephaga taxa were collected during this study (see Suppl. material 1). Of these, 62 were identified to the species level (Figs 3–13, Table 1), while the remaining 18 were identified to the genus level. Dytiscidae was the richest family, with 25 genera and 43 species, followed by Noteridae (6 genera; 32 species), Gyrinidae (2 genera; 3 species), and Haliplidae (1 genus; 2 species). Five species are recorded for the first time in Argentina: Copelatus cf. inornatus Sharp, 1882; Bidessodes cf. evanidus Young, 1986; *Neobidessus trilineatus* (Zimmermann, 1925); *Haliplus nieseri* van Vondel & Spangler, 2008; and Suphisellus cf. pereirai Guignot, 1958. Eight species are first recorded for the province of Corrientes: *Meridiorhantus orbignyi* (Balke, 1992); Celina cf. parallela (Babington, 1842); Laccophilus cf. obliquatus Régimbart, 1889; Laccophilus cf. paraguensis Régimbart, 1903; *Haliplus ornatipennis* Zimmermann, 1921; *Hydrocanthus paraguayensis* Zimmermann, 1928; *Mesonoterus crassicornis* (Régimbart, 1889); and Suphisellus cf. rufipes (Sharp, 1882). Fourteen taxa were exclusively collected using light traps.

**Table 1.** Hydradephagan beetles recorded in the Iberá wetlands during this study. Abbreviations for Argentine provinces: Bs.As.: Buenos Aires; Cba.: Córdoba; Cha.: Chaco; Chu.: Chubut; Cm.: Catamarca; Cs.: Corrientes; E.R.: Entre Ríos; Fo.: Formosa; Ju.: Jujuy; L.P.: La Pampa; L.R.: La Rioja; Mnes.: Misiones; Mza.: Mendoza; Nq.: Neuquén; R.N.: Río Negro; Sal.: Salta; S.E.: Santiago del Estero; S.J.: San Juan; S.L.: San Luis; S.Fe: Santa Fe; T.F.: Tierra del Fuego; Tuc.: Tucumán. Acronyms: LT: light trap; NA: new for Argentina; NCP: new for Corrientes Province.

Taxa	NA	NCP	Distribution	Habitat	LT	Figures
DYTISCIDAE						
Colymbetinae						
*Meridiorhantus orbignyi* (Balke, 1992)		X	Brazil, Uruguay, Argentina: Bs.As., Cs., E.R., Mnes., R.N., S.Fe	Lentic and lotic		Fig. 3A, B
*Rhantus signatus signatus* (Fabricius, 1775)			Bolivia, Brazil, Chile, Peru, Uruguay, Argentina: Bs.As., Cha., Chu., Cba., Cs., E.R., Ju., L.P., L.R., Mza., Mnes., Nq., R.N., Sal., S.J., S.L., S.Fe, T.F., Tuc.	Lentic and lotic	X	Fig. 3C, D
Copelatinae						
*Agaporomorphus mecolobus* Miller, 2001			Brazil, Argentina: Cs., Fo., Mnes.	Unknown	X	Fig. 4A, B
*Copelatus alternatus* Sharp, 1882			Brazil, Venezuela, Argentina: Cba., Cs., Tuc.	Lentic	X	Fig. 4C, D
*Copelatus caelatipennis* Aubé, 1838			Bolivia, Brazil, Guyana, Suriname, Venezuela, Argentina: Bs.As., Cha., Cs.	Lentic and lotic	X	Fig. 4E, F
Copelatus cf. inornatus Sharp, 1882	X	X	Bolivia, Argentina: Cs.	Unknown	X	Fig. 4G, H
*Copelatus longicornis* Sharp, 1882			Brazil, Suriname, Argentina: Bs.As., Cha., Cs., Mnes.	Lentic	X	Fig. 4I, J
Cybistrinae						
*Metaxydytes carcharias* (Griffini, 1895)			Brazil, Paraguay, Argentina: Bs.As., Cha., Cs., E.R., Fo., Ju., Sal., S.Fe	Lentic and lotic		Fig. 5A, B
*Metaxydytes laevigatus* (Olivier, 1791)			Bolivia, Brazil, Costa Rica, French Guiana, Guatemala, Mexico, Nicaragua, Panamá, Paraguay, Peru, Venezuela, Argentina: Bs.As., Cha., Cba., Cs., E.R., Fo., Mnes.	Lentic and lotic	X	Fig. 5C, D
*Trifurcitus robustus* (Aubé, 1838)			Brazil, Ecuador, Paraguay, Peru, Uruguay, Argentina: Bs.As., Cs., E.R., S.Fe	Unknown	X	Fig. 5E, F
Dytiscinae						
*Hydaticus xanthomelas* (Brullé, 1837)			Bolivia, Brazil, Paraguay, Peru, Argentina: Cs., E.R.	Lentic and lotic	X	Fig. 6A, B
*Notaticus fasciatus* Zimmermann, 1928			Bolivia, Brazil, Colombia, French Guiana, Paraguay, Uruguay, Venezuela, Argentina: Cha., Cs., Fo., Sal., S.Fe	Lentic and lotic	X	Fig. 6C, D
*Thermonectus nobilis* Zimmermann, 1924			Brazil, Paraguay, Peru, Argentina: Cs., Fo., Ju., Mnes., S.Fe	Lentic	X	Fig. 6E, F
*Thermonectus succinctus* (Aubé, 1838)			Bolivia, Brazil, Costa Rica, Cuba, Mexico, Paraguay, Peru, Uruguay, Argentina: Bs.As., Cha., Cba., Cs., E.R., Fo., Ju., L.P., L.R., Mnes., Sal., S.L., S.Fe, Tuc.	Lentic and lotic	X	Fig. 6G, H
Hydroporinae						
Bidessini						
*Anodocheilus maculatus* Babington, 1842			Brazil, French Guiana, Argentina: Bs.As., Cha., Cs., E.R., Mnes., S.Fe	Lentic and lotic	X	Fig. 7A, B
Bidessodes cf. evanidus Young, 1986	X	X	Brazil, Guyana, Suriname, Venezuela, Argentina: Cs.	Lentic	X	Fig. 7C, D
*Bidessonotus obtusatus* Régimbart, 1895			Bolivia, Brazil, Paraguay, Peru, Argentina: Cha., Cs., S.Fe	Lentic and lotic	X	Fig. 7E, F
*Brachyvatus acuminatus* (Steinheil, 1869)			Brazil, Argentina: Bs.As., Cha., Cs., E.R., S.Fe	Lentic and lotic	X	Fig. 7G, H
*Hemibidessus conicus* (Zimmermann, 1921)			Bolivia, Brazil, Paraguay, Argentina: Cha., Cs.	Lentic and lotic	X	Fig. 7I, J
*Neobidessus grandis* Pederzani & Rocchi, 2012			Argentina: Cs.	Unknown	X	Fig. 7K, L
*Neobidessus trilineatus* (Zimmermann, 1925)	X	X	Brazil, Argentina: Cs.	Lentic	X	Fig. 7M, N
Hydrovatini						
*Hydrovatus turbinatus* Zimmermann, 1921			Paraguay, Argentina: Bs.As., Cs., Sal.	Lentic		Fig. 7O, P
*Queda hydrovatoides* Zimmermann, 1921			Brazil, Argentina: Cs.	Lentic		Fig. 8A, B
Hyphydrini						
*Desmopachria concolor* Sharp, 1882			Brazil, Paraguay, Uruguay, Argentina: Bs.As., Cha., Cs., E.R., Mnes., S.Fe	Lentic and lotic	X	Fig. 8C, D
Methlini						
Celina cf. latipes (Brullé, 1836)			Brazil, Paraguay, Peru, Argentina: Cs.	Unknown	X	Fig. 8E, F
Celina cf. parallela (Babington, 1842)		X	Brazil, Argentina: Bs.As., Cs., E.R.	Lentic	X	Fig. 8G, H
Pachydrini						
*Pachydrus globosus* (Aubé, 1838)			Brazil, Paraguay, Puerto Rico, Argentina: Bs.As. Cha., Cba., Cs., E.R., Ju., S.Fe	Lentic and lotic		Fig. 8I, J
*Pachydrus obesus* Sharp, 1882			Brazil, Paraguay, Venezuela, Argentina: Bs.As., Cha., Cs., E.R., S.Fe	Lentic	X	Fig. 8K, L
Vatellini						
*Derovatellus lentus* (Wehncke, 1876)			Bolivia, Brazil, Colombia, Cuba, Dominica, Dominican Republic, Ecuador, French Guiana, Guadeloupe, Guatemala, Guyana, Panama, Paraguay, Peru, Puerto Rico, Suriname, Trinidad, Venezuela, Argentina: Bs.As., Cha., Cs., E.R., S.Fe, Tuc.	Lentic and lotic	X	Fig. 9A, B
*Vatellus haagi* Wehncke, 1876			Bolivia, Brazil, Paraguay, Uruguay, Argentina: Bs.As., Cha., Cba., Cs., E.R., Fo., Mnes., S.Fe	Lentic and lotic	X	Fig. 9C, D
*Vatellus wheeleri* Miller, 2005			Paraguay, Argentina: Bs.As., Cha., Cs.	Lentic	X	Fig. 9E, F
Laccophilinae						
*Laccomimus alvarengi* Toledo & Michat, 2015			Bolivia, Brazil, Ecuador, Panama, Paraguay, Peru, Suriname, Argentina: Cs.	Lentic	X	Fig. 9G, H
Laccophilus cf. obliquatus Régimbart, 1889		X	Brazil, Paraguay, Argentina: Bs.As., Cs.	Lentic and lotic	X	Fig. 9I, J
Laccophilus cf. paraguensis Régimbart, 1903		X	Brazil, Paraguay, Argentina: Bs.As., Cha., Cs., E.R.	Lentic and lotic	X	Fig. 9K, L
GYRINIDAE						
Gyrininae						
*Gyrinus violaceus* Régimbart, 1883			Brazil, Uruguay, Argentina: Cha., Cs., Mnes.	Lentic and lotic	X	Fig. 10A, B
HALIPLIDAE						
*Haliplus nieseri* van Vondel & Spangler, 2008	X	X	Brazil, Argentina: Cs.	Lentic		Fig. 10C, D
*Haliplus ornatipennis* Zimmermann, 1921		X	Bolivia, Brazil, Paraguay, Peru, Argentina: Bs.As., Cba., Cs., Fo., L.R., Sal., S.Fe, S.E., Tuc.	Lentic and lotic		Fig. 10E, F
NOTERIDAE						
Noterinae						
*Hydrocanthus debilis* Sharp, 188**2**			Belize, Bolivia, Brazil, Costa Rica, French Guiana, Guatemala, Mexico, Panama, Paraguay, Peru, Uruguay, Argentina: Bs.As., Cha., Cs., E.R., Fo., Mnes., Sal., S.Fe, Tuc.	Lentic and lotic	X	Fig. 11A, B
*Hydrocanthus levigatus* (Brullé, 1837)			Bolivia, Brazil, Guadeloupe, Panama, Paraguay, Venezuela, Argentina: Bs.As., Cm., Cha., Cs., E.R., Fo., Mnes., Sal., S.Fe, Tuc.	Lentic and lotic	X	Fig. 11C, D
*Hydrocanthus paraguayensis* Zimmermann, 1928		X	Bolivia, Brazil, Paraguay, Uruguay, Argentina: Bs.As., Cs.	Lentic		Fig. 11E, F
*Hydrocanthus sharpi* Zimmermann, 1928			Brazil, Ecuador, Argentina: Bs.As., Cha., Cs., E.R., Fo., Sal.	Lentic and lotic		Fig. 11G, H
*Hydrocanthus socius* Sahlberg, 1844			Bolivia, Brazil, Paraguay, Peru, Venezuela, Argentina: Cha., Cs., E.R., Fo.	Lentic and lotic	X	Fig. 11I, J
*Mesonoterus crassicornis* (Régimbart, 1889)		X	Brazil, Paraguay, Argentina: Cs., Fo.	Lentic	X	Fig. 11K, L
*Mesonoterus laevicollis* Sharp, 1882			Belize, Bolivia, Brazil, Costa Rica, Cuba, Guatemala, Mexico, Panama, Paraguay, Argentina: Cha., Cs., Fo., Mnes., S.Fe	Lentic and lotic	X	Fig. 11M, N
*Prionohydrus cambyreta* Urcola, Baca, Rodriguez & Michat, 2024			Argentina: Cs.	Unknown	X	Fig. 11O, P
*Suphis cimicoides* Aubé, 1837			Bolivia, Brazil, French Guiana, Guadeloupe, Mexico, Paraguay, Uruguay, Argentina: Bs.As., Cha., Cs., E.R., Fo., Mnes., Sal., S.Fe	Lentic and lotic	X	Fig. 12A, B
*Suphis freudei* Mouchamps, 1955			Paraguay, Argentina: Bs.As., Cha., Cs.	Lentic	X	Fig. 12C, D
*Suphis notaticollis* Zimmermann, 1921			Brazil, Paraguay, Argentina: Bs.As., Cha., Cs., E.R., Fo., Sal., S.Fe, S.E., Tuc.	Lentic and lotic	X	Fig. 12E, F
*Suphisellus balzani* (Régimbart, 1889)			Brazil, Paraguay, Argentina: Bs.As., Cha., Cs., E.R., Mnes., S.Fe, Tuc.	Lentic and lotic	X	Fig. 13A, B
*Suphisellus flavopictus* (Régimbart, 1889)			Bolivia, Paraguay, Venezuela, Argentina: Bs.As., Cha., Cs., E.R., Fo., Sal., S.Fe	Lentic and lotic	X	Fig. 13C, D
*Suphisellus grammicus* (Sharp, 1882)			Bolivia, Brazil, Paraguay, Peru, Argentina: Bs.As., Cha., Cba., Cs., E.R., Fo., Ju., Mnes., Sal., S.Fe, Tuc.	Lentic and lotic	X	Fig. 13E, F
*Suphisellus grossus* (Sharp, 1882)			Brazil, Paraguay, Argentina: Cha., Cs., Fo.	Lotic	X	Fig. 13G, H
Suphisellus cf. nigrinus (Aubé, 1838)			Antilles, Belize, Bolivia, Costa Rica, Cuba, Mexico, Paraguay, Argentina: Bs.As., Cha., Cs., E.R., Fo., L.R., Mnes., S.Fe, Tuc.	Lentic and lotic	X	Fig. 13I, J
Suphisellus cf. pereirai Guignot, 1958	X	X	Brazil, Argentina: Cs.	Lentic and lotic	X	Fig. 13K, L
*Suphisellus pinguiculus* (Régimbart, 1903)			Bolivia, Brazil, Argentina: Bs.As., Cs., Sal., S.Fe	Lentic and lotic	X	Fig. 13M, N
*Suphisellus punctipennis* (Sharp, 1882)			Bolivia, Brazil, Paraguay, Argentina: Bs.As., Cha., Cs., Fo., Sal., S.Fe, Tuc.	Lentic and lotic	X	Fig. 13O, P
*Suphisellus remator* (Sharp, 1882)			Bolivia, Brazil, Paraguay, Uruguay, Argentina: Bs.As., Cha., Cba., Cs., E.R., Fo., R.N., Sal., S.Fe, Tuc.	Lentic and lotic	X	Fig. 13Q, R
*Suphisellus rotundatus* (Sharp, 1882)			Bolivia, Brazil, Paraguay, Argentina: Bs.As., Cha., Cs., E.R., Fo., Sal., S.Fe	Lentic and lotic	X	Fig. 13S, T
Suphisellus cf. rufipes (Sharp, 1882)		X	Brazil, Cuba, Paraguay, Uruguay, Argentina: Bs.As., Cm., Cs., Ju., Sal., Tuc.	Lentic and lotic		Fig. 13U, V
*Suphisellus rufulus* Zimmermann, 1921			Brazil, Paraguay, Argentina: Cs.	Lentic and lotic		Fig. 13W, X
*Suphisellus sexnotatus* (Régimbart, 1889)			Brazil, Paraguay, Argentina: Cs.	Lentic	X	Fig. 13Y, Z
*Suphisellus variicollis* Zimmermann, 1921			Brazil, Paraguay, Argentina: Bs.As., Cs., S.Fe	Lentic and lotic	X	Fig. 13AA, AB

## ﻿Discussion

To date, no comprehensive inventory of Hydradephaga beetles has been available for the Iberá wetlands. Although our inventory remains incomplete and is subject to future updates as more fieldwork is conducted, the results clearly demonstrate that this area harbors a high diversity of these beetles. The genus and species richness recorded is comparable to that documented for the entire province of Corrientes. We recorded 25 genera (Michat et al. 2023) and 47 species (Gómez Lutz et al. 2012, 2015; Torres et al. 2012; Gómez Lutz and Kehr 2017) of Dytiscidae, two genera and four species (Michat and Archangelsky 2014) of Gyrinidae, one genus and two species (Archangelsky and Michat 2014) of Haliplidae, and six genera and 25 species (Urcola and Michat 2023) of Noteridae. These findings reinforce the importance of the Iberá wetlands as a priority conservation area.

Given that the study area is located approximately 35 km from Mburucuyá National Park, it is likely that the species reported by Torres et al. (2012), which were not recorded in this study, may yet be recorded in the future. These include *Trifurcitus fallax* (Aubé, 1838), *Hydrodytes opalinus* (Zimmermann, 1921), *Hemibidessus spiroductus* Miller, 2002, *Hydrovatus crassulus* Sharp, 1882, *Queda youngi* Biström, 1990, and *Suphisellus curtus* (Sharp, 1882).

According to the collected samples, the most widely distributed species in the Iberá wetlands are *Metaxydytes laevigatus* (Olivier, 1791), *Notaticus fasciatus* Zimmermann, 1928, *Thermonectus succinctus* (Aubé, 1838), *Anodocheilus maculatus* Babington, 1842, *Hemibidessus conicus* (Zimmermann, 1921), *Hydrocanthus debilis* Sharp, 1882, *Hydrocanthus sharpi* Zimmermann, 1928, *Hydrocanthus socius* Sahlberg, 1844, *Mesonoterus laevicollis* Sharp, 1882, *Suphis cimicoides* Aubé, 1837, *Suphisellus grammicus* (Sharp, 1882), Suphisellus cf. nigrinus (Aubé, 1838), *Suphisellus pinguiculus* (Régimbart, 1903), *Suphisellus punctipennis* (Sharp, 1882), *Suphisellus remator* (Sharp, 1882), *Suphisellus rotundatus* (Sharp, 1882), Suphisellus cf. rufipes, *Suphisellus rufulus* Zimmermann, 1921, *Suphisellus sexnotatus* (Régimbart, 1889), and *Suphisellus variicollis* Zimmermann, 1921. Some of these species, such as *M. laevigatus*, *N. fasciatus*, *T. succinctus*, *A. maculatus*, *H. debilis*, *M. laevicollis*, *S. cimicoides*, and S. cf. nigrinus, have been reported in other areas of Central and South America, suggesting a transcontinental distribution. This pattern highlights their ecological relevance and their ability to colonize a wide variety of aquatic habitats (see Suppl. material 1).

In Argentina, a total of 126 species of Dytiscidae (Michat et al. 2023), 26 species of Gyrinidae (Michat and Archangelsky 2014), 10 species of Haliplidae (Archangelsky and Michat 2014), and 45 species of Noteridae (Urcola and Michat 2023) have been recorded. Of these, the sampling conducted in the Iberá wetlands revealed the presence of 43 species of Dytiscidae, three species of Gyrinidae, two species of Haliplidae, and 32 species of Noteridae. These results indicate a significant representation of the families Dytiscidae and Noteridae in the wetlands, representing 34% and 71% of the species richness documented in Argentina for these families, respectively. In contrast, the diversity of Gyrinidae and Haliplidae is notably lower than that recorded for Argentina. The high proportion of Dytiscidae and Noteridae species in the Iberá wetlands could be related to the specific environmental characteristics of these wetlands, which appear to provide suitable habitats for these families. In contrast, the lower representation of Gyrinidae and Haliplidae may reflect lower ecological adaptability to the region’s conditions or simply a lower prevalence in the aquatic environments of the wetlands.

The records of C. cf. inornatus, B. cf. evanidus, *N. trilineatus*, *H. nieseri*, and S. cf. pereirai in the Iberá wetlands represent the first documented occurrences of these species in Argentina, significantly expanding their known distribution, previously restricted to South American regions including Brazil, Bolivia, Guyana, Suriname, and Venezuela (see Suppl. material 1). These findings, which may partly reflect historical sampling gaps in suitable Argentine habitats, substantially enhance our understanding of Hydradephaga diversity in the region. The observed distribution patterns suggest biogeographic connections among Neotropical wetlands, potentially facilitated by the Río de la Plata Basin network acting as a dispersal corridor for these aquatic species (Minotti 2016). While the presence of these taxa may reflect natural historical or recent dispersal processes, it could also stem from previous sampling gaps in suitable habitats. The strategic position of Iberá, connected to the Paraná and Uruguay river systems, supports its role as a key node for aquatic biodiversity.

These results not only reinforce the importance of Iberá as a critical refuge for aquatic beetle conservation in Argentina but also highlight the need for: (1) future molecular studies to elucidate the origin of these populations and their phylogeographic relationships, and (2) increased research efforts in understudied wetlands across the country, which may reveal new biogeographic patterns and cryptic diversity. The high Hydradephaga richness documented here underscores the unique ecological value of this ecosystem and its relevance for regional conservation initiatives.

## ﻿Additional information

### Use of AI

No use of AI was reported.

### Author ORCIDs

Matías R. Urcola https://orcid.org/0000-0002-1335-0072

Juan I. Urcola https://orcid.org/0000-0002-5684-2464

Mariano C. Michat https://orcid.org/0000-0002-1962-7976

Patricia L. M. Torres https://orcid.org/0000-0002-6532-9347

### Data availability

All of the data that support the findings of this study are available in the main text or Supplementary Information.

## Supplementary materials

Supplementary material 1 List of species Data type docx https://binary.pensoft.net/file/1462858This dataset is made available under the Open Database License (http://opendatacommons.org/licenses/odbl/1.0/). The Open Database License (ODbL) is a license agreement intended to allow users to freely share, modify, and use this Dataset while maintaining this same freedom for others, provided that the original source and author(s) are credited.Matías R. Urcola, Juan I. Urcola, Mariano C. Michat, Patricia L. M. Torres
