# Supplementary material for: ﻿The Hydradephaga (Coleoptera, Dytiscidae, Gyrinidae, Haliplidae, Noteridae) of the Iberá wetlands, the second largest wetland area of South America
Source: Zookeys. 2025 Nov 11;1259:287–307. doi: 10.3897/zookeys.1259.164084 (PMC12627999; doi:10.3897/zookeys.1259.164084)
Supplement: Supplementary material 1 — List of species [file zookeys-1259-287_article-164084__-s001.docx]

**The Hydradephaga (Coleoptera: Dytiscidae, Gyrinidae, Haliplidae, Noteridae) of the Iberá wetlands, the second largest wetland area of South America**

Matías R. Urcola, Juan I. Urcola, Mariano C. Michat and Patricia L.M. Torres

**Supplementary file.** List of species.

Family Dytiscidae Leach, 1815

Subfamily Colymbetinae Erichson, 1837

Genus *Meridiorhantus* Balke, Hájek and Hendrich, 2017

*Remarks:* This American genus of medium-sized beetles comprises five valid species (Nilsson and Hájek 2025), all of which are present in Argentina (Trémouilles 1984).

*Meridiorhantus orbignyi* (Balke, 1992)

Figs 3A, B

*Rhantus orbignyi* Balke, 1992: 38.

*Material examined:* **San Nicolás RS:** [28°07’41”S 57°26’04”W], 1 ♂, 13–15.xii.2013, coll. S. Mazzucconi.

*Distribution:* Brazil (Aubé 1838; Trémouilles 1984, 1998), Uruguay (Brullé 1837 (in Brullé 1835‒1842); Trémouilles 1984, 1998; Balke 1992), Argentina (provinces): Buenos Aires (Trémouilles 1984, 1998; Fontanarrosa et al. 2004; Fernández and López Ruf 2006; Alarie et al. 2009a; Macchia 2022; Macchia and Cicchino 2023), Corrientes (**new record**), Entre Ríos (Trémouilles 1984, 1998), Misiones (Fernández et al. 2008), Río Negro (Trémouilles 1984, 1998), Santa Fe (Trémouilles 1984, 1998; Balke 1993).

*Habitat:* This species has been found in a wide diversity of lotic (streams) and lentic (large lagoons, ponds, temporary rain pools) habitats. In most cases, associated with abundant aquatic vegetation (Fontanarrosa et al. 2004; Alarie et al. 2009a; Macchia 2022).

*Remarks*: *Meridiorhantus orbignyi* was listed as extinct in the 1996 IUCN Red List of Threatened Species (World Conservation Monitoring Centre 1996). However, numerous records have documented this species since 2004 (Fontanarrosa et al. 2004; Fernández and López Ruf 2006; Fernández et al. 2008; Alarie et al. 2009a; Macchia 2022; Macchia and Cicchino 2023; this study).

Genus *Rhantus* Dejean, 1833

*Remarks:* This genus of medium-sized beetles with a cosmopolitan distribution comprises 93 valid species (Nilsson and Hájek 2025). Only four species have been recorded in Argentina (Trémouilles 1998): *Rhantus calileguai* Trémouilles, 1984, *Rhantus duponti* (Aubé, 1838), *Rhantus obscuricollis* (Aubé, 1838), and *Rhantus signatus signatus* (Fabricius, 1775).

*Rhantus signatus signatus* (Fabricius, 1775)

Figs 3C, D

*Dytiscus signatus* Fabricius, 1775: 234.

*Material examined:* **Laguna Iberá RS:** 28°32’41”S 57°11’46”W, 1 ♀, 6–11.viii.1997, coll. F. Crespo and M. Iglesias. **San Nicolás RS:** 28°08’17”S 57°25’49”W, 4 ♂♂ and 2 ♀♀, 5.xi.2022, coll. M. Urcola; 28°07’42”S 57°26’02”W, 1 ♂, 9.xi.2022, light trap, coll. M. Urcola; 28°07’43”S 57°26’09”W, 1 ♂ and 1 ♀, 10.xi.2022, coll. J. Urcola.

*Distribution:* Bolivia (Régimbart 1899a; Roback et al. 1980), Brazil (Brullé 1837 (in Brullé 1835‒1842); Benetti et al. 2003; Benetti and Régil Cueto 2004), Chile (Aubé 1838; Babington 1842; Philippi and Philippi 1860; Régimbart 1903; Moroni 1988), Peru (Roback et al. 1980; Chaboo and Shepard 2015; Bustamante 2018), Uruguay (Brullé 1837 (in Brullé 1835‒1842); Babington 1842; Benetti and Garrido 2004), Argentina (provinces): Buenos Aires (Régimbart 1889a; Régimbart 1903; Bruch 1915; Trémouilles 1984; Moroni 1988; Fernández and López Ruf 1999, 2006; von Ellenrieder and Fernández 2000; Fontanarrosa et al. 2004, 2009; Alarie et al. 2009a; Macchia 2022; Macchia and Cicchino 2023), Chaco (Régimbart 1903), Chubut (Trémouilles 1984), Córdoba (Régimbart 1903; Viana and Williner 1972; Trémouilles 1984; Corigliano and Raffaini 2001), Corrientes (Gómez Lutz et al. 2012), Entre Ríos (Trémouilles 1984; Torres et al. 2007), Jujuy (Trémouilles 1984; Moroni 1988; Torres et al. 2008), La Pampa (Trémouilles 1984), La Rioja (Trémouilles 1984), Mendoza (Bruch 1915), Misiones (Bruch 1915; Trémouilles 1984; Fernández et al. 2008), Neuquén (Trémouilles 1984), Río Negro (Brullé 1837 (in Brullé 1835‒1842); Bruch 1915; Trémouilles 1984), Salta (Régimbart 1903; Trémouilles 1984), San Juan (Trémouilles 1984), San Luis (Bruch 1915; Viana and Williner 1972; Trémouilles 1984), Santa Fe (Bruch 1915), Tierra del Fuego (Babington 1842; Trémouilles 1984), Tucumán (Trémouilles 1984).

*Habitat:* This widely distributed species has been found in a wide diversity of habitats (e.g., streams, lagoons, swamps, permanent ponds, temporary pools, water storage tanks for livestock) (Torres et al. 2007, 2008; Gómez Lutz et al. 2012; Macchia 2022). In this study, specimens of *R. signatus* were collected from a small grassland stream and a permanent pond, as well as using light traps.

Subfamily Copelatinae Branden, 1884

Genus *Agaporomorphus* Zimmermann, 1921

*Remarks:* This genus of small Neotropical beetles comprises 12 valid species (Nilsson and Hájek 2025). In Argentina only *Agaporomorphus mecolobus* Miller, 2001 has been recorded (Torres et al. 2012).

*Agaporomorphus mecolobus* Miller, 2001

Figs 4A, B

*Agaporomorphus mecolobus* Miller, 2001: 527, figs 10‒12, 18, 23, 28, 36.

*Material examined:* **Laguna Iberá RS:** 28°32’46”S 57°11’45”W, 1 ♂ and 2 ♀♀, 8.xi.2019, light trap, coll. G. Rodriguez and J. Urcola; 28°32’46”S 57°11’45”W, 6 ♂♂ and 5 ♀♀, 14.xi.2019, light trap, coll. G. Rodriguez and J. Urcola. **San Nicolás RS:** 28°07’42”S 57°26’02”W, 1 ♂, 9.xi.2022, light trap, coll. M. Urcola.

*Distribution:* Brazil (Miller 2001; Hendrich et al. 2015), Argentina (provinces): Corrientes (Libonatti et al. 2011; Torres et al. 2012), Formosa (M. Michat, pers. obs.), Misiones (M. Michat, pers. obs.).

*Habitat:* To date, the habitat of this species remains unknown; however, it is frequently collected using light traps (Miller 2001; Torres et al. 2012; Hendrich et al. 2015; this study).

Genus *Copelatus* Erichson, 1832

*Remarks:* This cosmopolitan genus of medium sized beetles currently includes 449 valid species (Nilsson and Hájek 2025). In Argentina, eight species were reported (Trémouilles 1998): *Copelatus caelatipennis* Aubé, 1838, *Copelatus consors* Sharp, 1882, *Copelatus dimorphus* Sharp, 1882, *Copelatus incognitus* Sharp, 1882, *Copelatus integer* Sharp, 1882, *Copelatus longicornis* Sharp, 1882, *Copelatus restrictus* Sharp, 1882, and *Copelatus silvestrii* Régimbart, 1903.

*Copelatus alternatus* Sharp, 1882

Figs 4C, D

*Copelatus alternatus* Sharp, 1882a: 576.

*Material examined:* **Cambyretá RS:** [27°49’00”S 56°50’00”W], 1 ♂, 16.xi.2018, light trap, coll. S. Mazzucconi, G. Rodriguez and J. Urcola. **Laguna Iberá RS:** 28°32’49”S 57°11’48”W, 2 spec, 17.ii.2018, coll. M. Michat.

*Distribution:* Brazil (Sharp 1882a), Venezuela (Régimbart 1889b), Argentina (provinces): Córdoba (Viana and Williner 1978), Corrientes (Gómez Lutz et al. 2012, 2015; Gómez Lutz and Kehr 2017), Tucumán (Alarie et al. 2022).

*Habitat:* In Corrientes Province, this species has been found in small lagoons, rice fields and temporary ponds (Gómez Lutz et al. 2012, 2015; Gómez Lutz and Kehr 2017). It was also collected using a light trap (this study).

*Copelatus caelatipennis* Aubé, 1838

Figs 4E, F

*Copelatus caelatipennis* Aubé, 1838: 382.

*Material examined:* **Laguna Iberá RS:** 28°32’46”S 57°11’45”W, 4 spec, 8.xi.2019, light trap, coll. G. Rodriguez and J. Urcola.

*Distribution:* Bolivia (Régimbart 1899a), Brazil (Aubé 1838; Sharp 1882b; Régimbart 1889c; Zimmermann 1921; Benetti et al. 2003; Benetti and Régil Cueto 2004), Guyana (Régimbart 1889c), Suriname (Régimbart 1889c), Venezuela (Régimbart 1889b), Argentina (provinces): Buenos Aires (Bruch 1915), Chaco (Libonatti et al. 2013), Corrientes (Torres et al. 2012).

*Habitat:* This species has been found in rivers with very slow currents and abundant vegetation, streams and semi-permanent ponds (Benetti and Régil Cueto 2004; Libonatti et al. 2013). In this study, as in Torres et al. (2012), the specimens were collected using light traps.

*Copelatus* cf. *inornatus* Sharp, 1882

Figs 4G, H

*Copelatus inornatus* Sharp, 1882a: 569.

*Material examined:* **Laguna Iberá RS:** 28°32’46”S 57°11’45”W, 1 ♀, 14.xi.2019, light trap, coll. G. Rodriguez and J. Urcola.

*Distribution:* Bolivia (Grant et al. 2025), Argentina: Corrientes Province (**new record**).

*Habitat:* Currently unknown, the only specimen studied was collected using a light trap.

*Copelatus longicornis* Sharp, 1882

Figs 4I, J

*Copelatus longicornis* Sharp, 1882a: 570.

*Material examined:* **Cambyretá RS:** [27°49’00”S 56°50’00”W], 1 ♂, 16.xi.2018, light trap, coll. S. Mazzucconi, G. Rodriguez and J. Urcola. **Laguna Iberá RS:** 28°32’46”S 57°11’45”W, 26 ♂♂ and 52 ♀♀, 14.xi.2019, light trap, coll. G. Rodriguez and J. Urcola.

*Distribution:* Brazil (Sharp 1882a; Zimmermann 1921; Benetti et al. 2003; Benetti and Régil Cueto 2004), Suriname (Régimbart 1889c), Argentina (provinces): Buenos Aires (Bruch 1915; Michat and Torres 2009), Chaco (Régimbart 1889a; Bruch 1915; Libonatti et al. 2013), Corrientes (Gómez Lutz et al. 2012, 2015; Gómez Lutz and Kehr 2017), Misiones (Bruch 1915).

*Habitat:* This species has been found in small lagoons, rice fields and temporary ponds (Gómez Lutz et al. 2012, 2015; Gómez Lutz and Kehr 2017). The specimens studied here, like those reported by Libonatti et al. (2013), were collected using light traps.

Subfamily Cybistrinae Sharp, 1880

Genus *Metaxydytes* Miller, Michat and Ferreira-Jr, 2024

*Remarks:* This American genus comprises large aquatic beetles. Currently, nine species are known (Nilsson and Hájek 2025). In Argentina, the following species were found (Trémouillles and Bachmann 1980): *Metaxydytes carcharias* (Griffini, 1895), *Metaxydytes fraternus* (Sharp, 1882), *Metaxydytes laevigatus* (Olivier, 1791), and *Metaxydytes marginithorax* (Perty, 1830).

*Metaxydytes carcharias* (Griffini, 1895)

Figs 5A, B

*Megadytes carcharias* Griffini, 1895: 5.

*Material examined:* **San Nicolás RS:** 28°07’39”S 57°25’59”W, 1 ♀, 8.xi.2022, coll. J. Urcola.

*Distribution:* Brazil (Trémouilles 1989a; Benetti et al. 2003; Benetti and Régil Cueto 2004), Paraguay (Griffini 1895; Mouchamps 1957; Trémouilles and Bachmann 1980), Argentina (provinces): Buenos Aires (Trémouilles and Bachmann 1980), Chaco (Trémouilles and Bachmann 1980; Michat 2010; Libonatti et al. 2013), Corrientes (Trémouilles and Bachmann 1980), Entre Ríos (Trémouilles and Bachmann 1980; Torres et al. 2007), Formosa (Trémouilles and Bachmann 1980), Jujuy (Torres et al. 2008), Salta (Trémouilles and Bachmann 1980), Santa Fe (Mouchamps 1957; Trémouilles and Bachmann 1980).

*Habitat:* The specimen studied here was collected in a habitat very similar to that reported for this species in previous studies (Torres et al. 2007; Libonatti et al. 2013). This habitat consisted of a small pond with muddy bottom and very turbid water, due to abundant decomposing organic matter and a high concentration of filamentous algae. This species was also found in a small deep lagoon, with floating vegetation and abundant organic matter (Torres et al. 2008) and in a stream with muddy bottom, relatively clear water and almost completely covered with floating vegetation (Libonatti et al. 2013).

*Metaxydytes laevigatus* (Olivier, 1791)

Figs 5C, D

*Dytiscus laevigatus* Olivier, 1791: 308.

*Material examined:* **Galarza RS:** [28°05’51”S 56°41’49”W], 1 ♂, 18.iv.2013, coll. S. Mazzucconi. **Itatí RS:** 28°44’56”S 58°07’11”W, 1 ♀, 30.iii.2014, coll. S. Mazzucconi. **Laguna Iberá RS:** 28°34’13”S 57°10’13”W, 2 ♂♂, 16.ii.2018, coll. M. Michat; 28°32’49”S 57°11’48”W, 1 ♂, 17.ii.2018, coll. M. Michat; 28°32’46”S 57°11’45”W, 1 ♀, 8.xi.2019, light trap, coll. G. Rodriguez and J. Urcola. **San Nicolás RS:** [28°07’41”S 57°26’04”W], 1 ♀, 13–15.xii.2013, coll. S. Mazzucconi; 28°07’43”S 57°26’09”W, 1 ♂, 6.xi.2022, coll. M. Urcola.

*Distribution:* Bolivia (Brullé 1837 (in Brullé 1835‒1842); Régimbart 1899a), Brazil (Sharp 1882b; Trémouilles and Bachmann 1980; Trémouilles 1989a; Benetti and Régil Cueto 2004), Costa Rica (Blanco Aller and Régil 2013; Blanco Aller 2016), French Guiana (Olivier 1791; Régimbart 1904), Guatemala (Sharp 1882b), Mexico (Sharp 1882b), Nicaragua (Sharp 1882b), Panama (Sharp 1882b), Paraguay (Griffini 1895; Trémouilles and Bachmann 1980; Trémouilles 1989a), Peru (Bustamante 2018), Venezuela (Régimbart 1889b), Argentina (provinces): Buenos Aires (Bruch 1915), Chaco (Trémouilles and Bachmann 1980; Michat 2010; Libonatti et al. 2013), Córdoba (Bruch 1915), Corrientes (Brullé 1837 (in Brullé 1835‒1842); Trémouilles and Bachmann 1980; Gómez Lutz et al. 2012, 2015), Entre Ríos (Torres et al. 2007), Formosa (Trémouilles and Bachmann 1980), Misiones (Bruch 1915; Trémouilles 1989a).

*Habitat:* This species lives in a wide diversity of habitats. It has been found in small ponds with turbid water, muddy bottom and a large amount of organic matter (Torres et al. 2007; this study), in shallow lagoons with semi-transparent waters and with different proportions of aquatic macrophytes (Torres et al. 2007; Gómez Lutz et al. 2012) and in a stream with a muddy bottom, relatively clear water and completely covered with floating vegetation (Libonatti et al. 2013). Gómez Lutz et al. (2015) found *M. laevigatus* in rice fields in the province of Corrientes. Specimens were also collected using light traps (Torres et al. 2007; this study).

Genus *Trifurcitus* Brinck, 1945

*Remarks:* This genus of large aquatic beetles with a Neotropical distribution, currently includes six species (Nilsson and Hájek 2025). In Argentina, only two species were found (Trémouillles and Bachmann 1980): *Trifurcitus fallax* (Aubé, 1838), and *Trifurcitus robustus* (Aubé, 1838).

*Trifurcitus robustus* (Aubé, 1838)

Figs 5E, F

*Cybister robustus* Aubé, 1838: 49, 50.

*Material examined:* **Laguna Iberá RS:** 28°32’49”S 57°11’44”W, 1 ♂, 16.ii.2018, light trap, coll. M. Michat.

*Distribution:* Brazil (Aubé 1838), Ecuador (Caputo et al. 2006), Paraguay (Trémouilles 1989a), Peru (Miller et al. 2007), Uruguay (Trémouilles and Bachmann 1980), Argentina (provinces): Buenos Aires (Sharp 1882a; Trémouilles and Bachmann 1980), Corrientes (Trémouilles and Bachmann 1980), Entre Ríos (Michat 2006); Santa Fe (Trémouilles and Bachmann 1980; Trémouilles 1989a).

*Habitat:* Very little information exists about the biology of this species. Caputo et al. (2006) reported attack events by *T. robustus* adults on young turtles in three artificial pools of the “Reserva de Producción de Fauna Cubayeno” in Ecuador. The specimen studied here was collected using a light trap.

Subfamily Dytiscinae Leach, 1815

Genus *Hydaticus* Leach, 1817

*Remarks:* The cosmopolitan genus *Hydaticus* comprises 160 species of medium-sized beetles (Nilsson and Hácek 2025). In Argentina, only three species were found (Trémouilles 1996, 1998): *Hydaticus palliatus* Aubé, 1838, *Hydaticus tuyuensis* Trémouilles, 1996, and *Hydaticus xanthomelas* (Brullé, 1837).

*Hydaticus xanthomelas* (Brullé, 1837)

Figs 6A, B

*Dytiscus xanthomelas* Brullé, 1837 (in Brullé 1835‒1842): 47, 48.

*Material examined:* **Laguna Iberá RS:** 28°32’49”S 57°11’48”W, 1 ♂, 17.ii.2018, coll. M. Michat; 28°32’49”S 57°11’44”W, 1 ♀, 17.ii.2018, light trap, coll. M. Michat; 28°32’46”S 57°11’45”W, 7 ♂♂ and 8 ♀♀, 8.xi.2019, light trap, coll. G. Rodriguez and J. Urcola; 28°32’46”S 57°11’45”W, 9 ♂♂ and 3 ♀♀, 14.xi.2019, light trap, coll. G. Rodriguez and J. Urcola.

*Distribution:* Bolivia (Miller et al. 2007, 2009), Brazil (Sharp 1882a; Trémouilles 1996; Ferreira-Jr et al. 1998; Benetti and Hamada 2003; Megna et al. 2019), Paraguay (Trémouilles 1996; Megna et al. 2019), Peru (Megna et al. 2019), Argentina (provinces): Corrientes (Brullé 1837 (in Brullé 1835‒1842); Bruch 1915), Entre Ríos (Trémouilles 1996).

*Habitat:* This species has been found in lentic habitats (semi-permanent pools) (Ferreira-Jr et al. 1998) and in lotic habitats (streams) with slow to moderate water flow (Benetti and Hamada 2003). Gómez Lutz et al. (2012) reported an unidentified species of *Hydaticus* from a shallow lagoon located in the northwest of Corrientes Province.

Genus *Notaticus* Zimmermann, 1928

*Remarks:* This genus of medium-sized beetles comprises two species: *Notaticus fasciatus* Zimmermann, 1928 (described from Brazil), and *Notaticus obscurus* García and Navarro, 2001 (described from Venezuela). In Argentina so far only *N. fasciatus* has been found.

*Notaticus fasciatus* Zimmermann, 1928

Figs 6C, D

*Notaticus fasciatus* Zimmermann, 1928: 183.

*Material examined:* **Galarza RS:** [28°04’54”S 56°42’07”W], 1 ♂ and 1 ♀, 16.iv.2013, coll. S. Mazzucconi; [28°05’24”S 56°42’33”W], 1 ♂, 18.iv.2013, coll. S. Mazzucconi; [28°04’50”S 56°44’16”W], 1 ♀, 19.iv.2013, coll. S. Mazzucconi. **Laguna Iberá RS:** 28°35’27”S 57°10’28”W, 1 ♀, 1.xii.2012, coll. S. Mazzucconi; 28°33’54”S 57°12’59”W, 1 ♂, 15.ii.2018, coll. M. Michat; 28°33’21”S 57°11’40”W, 1 ♂, 16.ii.2018, coll. S. Mazzucconi; 28°32’46”S 57°11’45”W, 2 ♂♂, 8.xi.2019, light trap, coll. G. Rodriguez and J. Urcola; 28°32’46”S 57°11’45”W, 14 ♂♂ and 16 ♀♀, 14.xi.2019, light trap, coll. G. Rodriguez and J. Urcola. **San Nicolás RS:** 28°10’06”S 57°25’47”W, 4 ♀♀, 14.xii.2013, coll. S. Mazzucconi.

*Distribution:* Bolivia (Spangler 1973; Miller et al. 2007, 2009), Brazil (Zimmermann 1928; Guignot 1942; Spangler 1973), Colombia (Spangler 1973), French Guiana (Miller et al. 2007), Paraguay (Spangler 1973; Trémouilles and Bachmann 1981), Uruguay (Zimmermann 1928; Spangler 1973), Venezuela (Spangler 1973; García and Navarro 2001), Argentina (provinces): Chaco (Trémouilles and Bachmann 1981; Libonatti et al. 2013), Corrientes (Trémouilles and Bachmann 1981; Michat and Alarie 2009; Gómez Lutz et al. 2012; Torres et al. 2012), Formosa (Trémouilles and Bachmann 1981), Salta (Trémouilles and Bachmann 1981), Santa Fe (Spangler 1973; Trémouilles and Bachmann 1981; Macchia et al. 2015).

*Habitat: Notaticus fasciatus* lives in a wide variety of aquatic habitats. This species has been found in rivers and streams with very slow current, muddy bottom and covered with floating vegetation (Torres et al. 2012; Libonatti et al. 2013; this study). It was also found in small lagoons, ponds and ditches, with turbid water, muddy or sandy bottom and abundant floating vegetation (Spangler 1973; Gómez Lutz et al. 2012; Torres et al. 2012; Libonatti et al. 2013; this study). Specimens of this species were also collected using a light trap (Macchia et al. 2015; this study).

Genus *Thermonectus* Dejean, 1833

*Remarks:* The American genus *Thermonectus* comprises a group of medium-sized beetles that includes 20 valid species (Nilsson and Hájek 2025). Of these, only six species have been recorded in Argentina: *Thermonectus alfredi* Griffini, 1898, *Thermonectus circumscriptus* (Latreille, 1809), *Thermonectus margineguttatus* (Aubé, 1838), *Thermonectus nobilis* Zimmermann, 1924, *Thermonectus succinctus* (Aubé, 1838), and *Thermonectus tremouillesi* Michat and Torres, 2016.

*Thermonectus nobilis* Zimmermann, 1924

Figs 6E, F

*Thermonectes nobilis* Zimmermann, 1924: 3.

*Material examined:* **Laguna Iberá RS:** 28°32’49”S 57°11’44”W, 2 ♂♂ and 2 ♀♀, 14.ii.2018, light trap, coll. M. Michat; 28°32’49”S 57°11’44”W, 1 ♀, 15.ii.2018, light trap, coll. M. Michat; 28°32’46”S 57°11’43”W, 2 ♀♀, 16.ii.2018, coll. M. Michat; 28°32’49”S 57°11’44”W, 3 ♂♂ and 3 ♀♀, 16.ii.2018, light trap, coll. M. Michat; 28°32’49”S 57°11’48”W, 9 ♂♂ and 6 ♀♀, 17.ii.2018, coll. M. Michat; 28°32’49”S 57°11’44”W, 1 ♀, 17.ii.2018, light trap, coll. M. Michat; 28°32’46”S 57°11’45”W, 7 ♂♂ and 5 ♀♀, 8.xi.2019, light trap, coll. G. Rodriguez and J. Urcola; 28°32’46”S 57°11’45”W, 15 ♂♂ and 8 ♀♀, 14.xi.2019, light trap, coll. G. Rodriguez and J. Urcola.

*Distribution:* Brazil (Zimmermann 1924; Trémouilles 1989b), Paraguay (Trémouilles 1989), Peru (Miller et al. 2007), Argentina (provinces): Corrientes (Trémouilles 1989b; Gómez Lutz et al. 2012, 2015; Torres et al. 2012; Gómez Lutz and Kehr 2017), Formosa (Trémouilles 1989b), Jujuy (Torres et al. 2008), Misiones (Trémouilles 1989b; Fernández et al. 2008), Santa Fe (Trémouilles 1989b).

*Habitat: Thermonectus nobilis* has been found in lentic habitats with muddy bottom, abundant organic matter and floating vegetation (e.g., lagoons, marshes, temporary ponds and rice fields) (Torres et al. 2008, 2012; Gómez Lutz et al. 2012, 2015; Gómez Lutz and Kehr 2017). In this study, specimens were also collected using light traps.

*Thermonectus succinctus* (Aubé, 1838)

Figs 6G, H

*Acilius succinctus* Aubé, 1838: 145‒147.

*Material examined:* **Galarza RS:** [28°05’50”S 56°41’52”W], 1 ♀, 18.iv.2013, coll. S. Mazzucconi. **Itatí RS:** 28°44’17”S 58°08’10”W, 1 ♂, 26.ix.2003, coll. S. Mazzucconi; 28°44’56”S 58°07’11”W, 5 ♂♂ and 1 ♀, 30.iii.2014, coll. S. Mazzucconi. **Laguna Iberá RS:** 28°32’49”S 57°11’44”W, 1 ♂ and 1 ♀, 16.ii.2018, light trap, coll. M. Michat; 28°32’46”S 57°11’45”W, 1 ♀, 8.xi.2019, light trap, coll. G. Rodriguez and J. Urcola; 28°32’46”S 57°11’45”W, 2 ♂♂ and 4 ♀♀, 14.xi.2019, light trap, coll. G. Rodriguez and J. Urcola. **San Nicolás RS:** [28°07’41”S 57°26’04”W], 2 ♀♀, 13–15.xii.2013, light trap, coll. S. Mazzucconi; 28°07’20”S 57°25’57”W, 1 ♀, 9.xi.2022, coll. M. Urcola. **Yahaveré RS:** [28°32’30”S 57°44’45”W], 1 ♂, 4–9.xi.2015, coll. S. Mazzucconi.

*Distribution:* Bolivia (Régimbart 1899a; Trémouilles 1989b; Miller et al. 2007), Brazil (Aubé 1838; Laporte 1840; Sharp 1882b; Trémouilles 1989b; Ferreira-Jr et al. 1998; Benetti et al. 2003; Benetti and Régil Cueto 2004), Costa Rica (Blanco Aller 2016), Cuba (Alarie et al. 2009b; Megna and Sánchez-Fernández 2016), Mexico (Aubé 1838; Sharp 1882b; Horn 1894; Arce-Pérez 1995), Paraguay (Aubé 1838; Régimbart 1903; Trémouilles 1989b), Peru (Sharp 1882b; Chaboo and Shepard 2015), Uruguay (Benetti and Garrido 2004), Argentina (provinces): Buenos Aires (Régimbart 1889a; Régimbart 1903; Bruch 1915; Zimmermann 1919; Viana 1937; Trémouilles 1989b; Fernández and López Ruf 1999; von Ellenrieder and Fernández 2000; Fontanarrosa et al. 2004, 2009; Michat and Torres 2005; Fernández et al. 2010; Macchia and Cicchino 2023), Chaco (Régimbart 1889a; Trémouilles 1989b; Libonatti et al. 2013), Córdoba (Trémouilles 1989b; Corigliano and Raffaini 2001), Corrientes (Trémouilles 1989b; Gómez Lutz et al. 2012, 2015; Torres et al. 2012; Gómez Lutz and Kehr 2017), Entre Ríos (Trémouilles 1989b; Torres et al. 2007), Formosa (Trémouilles 1989b), Jujuy (Trémouilles 1989b; Torres et al. 2008), La Pampa (Trémouilles 1989b), La Rioja (Trémouilles 1989b), Misiones (Bruch 1915; Trémouilles 1989b; Fernández et al. 2008), Salta (Régimbart 1903; Bruch 1915; Trémouilles 1989b), San Luis (Bruch 1915), Santa Fe (Macchia et al. 2015), Tucumán (Trémouilles 1989b).

*Habitat:* This species has been found in small lagoons, ponds and temporary pools with abundant aquatic vegetation (Fontanarrosa et al. 2004, 2009; Torres et al. 2007, 2008, 2012; Gómez Lutz et al. 2012; Libonatti et al. 2013; Gómez Lutz and Kehr 2017; Macchia and Cicchino 2023). It was also collected in rivers and streams with very slow current (Libonatti et al. 2013), rice fields (Gómez Lutz et al. 2015), and through light traps (Torres et al. 2007; Libonatti et al. 2013; Macchia et al. 2015; this study).

Subfamily Hydroporinae Aubé, 1836

Genus *Anodocheilus* Babington, 1842

*Remarks:* This American genus of small beetles comprises 22 species (Nilsson and Hájek 2025). Only two species have been recorded in Argentina (Trémouilles 1998): *Anodocheilus maculatus* Babington, 1842, and *Anodocheilus silvestrii* Régimbart, 1903.

*Anodocheilus maculatus* Babington, 1842

Figs 7A, B

*Anodocheilus maculatus* Babington, 1842: 16, pl. 1, fig. 4

*Material examined:* **Cambyretá RS:** 27°49’00”S 56°50’00”W, 1 spec, 16.xi.2018, light trap, coll. S. Mazzucconi, G. Rodriguez and J. Urcola. **Itatí RS:** 28°44’56”S 58°07’12”W, 1 spec, 30.iii.2014, coll. S. Mazzucconi. **Laguna Iberá RS:** 28°32’46”S 57°11’45”W, 1 ♂, 8.xi.2019, light trap, coll. G. Rodriguez and J. Urcola; 28°32’49”S 57°11’44”W, 9 spec, 14.ii.2018, light trap, coll. M. Michat; 28°33’54”S 57°12’59”W, 41 spec, 15.ii.2018, coll. M. Michat; 28°32’49”S 57°11’48”W, 20 spec, 17.ii.2018, coll. M. Michat. **San Nicolás RS:** 28°07’51”S 57°26’02”W, 27 spec, 4.xi.2022, coll. M. Urcola; 28°08’17”S 57°25’49”W, 30 spec, 5.xi.2022, coll. M. Urcola; 28°07’43”S 57°26’09”W, 9 spec, 6.xi.2022, coll. M. Urcola; 28°10’48”S 57°26’46”W, 26 spec, 7.xi.2022, coll. M. Urcola; 28°09’25”S 57°26’34”W, 2 spec, 8.xi.2022, coll. M. Urcola; 28°07’39”S 57°25’59”W, 51 spec, 8.xi.2022, coll. M. Urcola; 28°07’20”S 57°25’57”W, 88 spec, 9.xi.2022, coll. M. Urcola; 28°07’43”S 57°26’23”W, 5 spec, 9.xi.2022, coll. M. Urcola; 28°07’43”S 57°26’09”W, 5 spec, 9.xi.2022, coll. M. Urcola; 28°07’42”S 57°26’02”W, 9 spec, 9.xi.2022, light trap, coll. M. Urcola.

*Distribution:* Brazil (Babington 1842; Zimmermann 1921; Benetti and Régil Cueto 2004), French Guiana (Régimbart 1904), Argentina (provinces): Buenos Aires (Bruch 1915; Zimmermann 1919; von Ellenrieder and Fernández 2000), Chaco (Libonatti et al. 2013), Corrientes (Gómez Lutz et al. 2012, 2015; Torres et al. 2012; Gómez Lutz and Kehr 2017), Entre Ríos (Michat and Torres 2006; Torres et al. 2007), Misiones (Bruch 1915), Santa Fe (Macchia et al. 2015).

*Habitat:* This species has been found in small lagoons (Gómez Lutz et al. 2012) and shallow ponds with high insolation, abundant aquatic vegetation and organic matter (von Ellenrieder and Fernández 2000; Torres et al. 2007, 2012; Libonatti et al. 2013; Gómez Lutz and Kehr 2017; this study) and in small streams with shallow depth, very slow current, high exposure to sunlight and abundant aquatic vegetation (Torres et al. 2012; Libonatti et al. 2013). It was also found in rice fields (Gómez Lutz et al. 2015) and was collected using a light trap (Torres et al. 2012; Libonatti et al. 2013; Macchia et al. 2015; this study).

Genus *Bidessodes* Régimbart, 1895

*Remarks:* *Bidessodes* comprises a group of small Neotropical beetles with 20 valid species (Nilsson and Hájek 2025). Specimens of this genus are present in Argentina, although they have not been recognized at a specific level (Trémouilles 1998; Libonatti et al. 2011; Macchia et al. 2015).

*Bidessodes* cf. *evanidus* Young, 1986

Figs 7C, D

*Bidessodes evanidus* Young, 1986: 212, 213, figs 16‒19.

*Material examined:* **Laguna Iberá RS:** 28°32’46”S 57°11’45”W, 2 ♀♀, 8.xi.2019, light trap, coll. G. Rodriguez and J. Urcola. **San Nicolás RS:** 28°07’20”S 57°25’57”W, 1 ♂, 9.xi.2022, coll. J. Urcola.

*Distribution:* Brazil (Young 1986; Miller 2017), Guyana (Miller 2017), Suriname (Young 1986; Miller 2017), Venezuela (Young 1986; Miller 2017), Argentina: Corrientes Province (**new record**).

*Habitat:* This species has been found in a clear-water pond at the edge of a marsh (Young 1986), but also in a muddy pond with abundant macrophytes and filamentous algae (this study). Specimens were also collected using light traps (Young 1986; this study).

Genus *Bidessonotus* Régimbart, 1895

*Remarks:* This American genus of small beetles currently is made up of 36 species (Nilsson and Hájek 2025). In Argentina, only *Bidessonotus obtusatus* Régimbart, 1895 has been recorded (Miller 2016).

*Bidessonotus obtusatus* Régimbart, 1895

Figs 7E, F

*Bidessonotus obtusatus* Régimbart, 1895: 336.

*Material examined:* **Laguna Iberá RS:** 28°32’49”S 57°11’44”W, 3 ♂♂ and 2 ♀♀, 14.ii.2018, light trap, coll. M. Michat; 28°32’49”S 57°11’48”W, 1 ♂, 17.ii.2018, coll. M. Michat; 28°32’49”S 57°11’44”W, 1 ♀, 17.ii.2018, light trap, coll. M. Michat. **San Nicolás RS:** 28°07’43”S 57°26’09”W, 1 spec, 6.xi.2022, coll. M. Urcola; 28°07’42”S 57°26’02”W, 1 spec, 8.xi.2022, light trap, coll. M. Urcola; 28°07’20”S 57°25’57”W, 1 spec, 9.xi.2022, coll. M. Urcola; 28°07’43”S 57°26’23”W, 3 spec, 9.xi.2022, coll. M. Urcola; 28°07’42”S 57°26’02”W, 3 spec, 9.xi.2022, light trap, coll. M. Urcola.

*Distribution:* Bolivia (Balfour-Browne 1947), Brazil (Régimbart 1895; Balfour-Browne 1947), Paraguay (Régimbart 1895), Peru (Chaboo and Shepard 2015), Argentina (provinces): Chaco (Libonatti et al. 2013), Corrientes (Torres et al. 2012), Santa Fe (Macchia et al. 2015).

*Habitat:* This species has been found in small shallow lagoons with high exposure to sunlight, abundant aquatic vegetation and organic matter, as well as in small streams with abundant vegetation, high insolation and slow current (Torres et al. 2012; Libonatti et al. 2013; this study). It was also collected using light traps (Torres et al. 2012; Libonatti et al. 2013; Macchia et al. 2015; this study).

Genus *Brachyvatus* Zimmermann, 1919

*Remarks:* This American genus of tiny beetles includes four species (Nilsson and Hájek 2025). In Argentina, only *Brachyvatus acuminatus* (Steinheil, 1869) has been reported (Trémouilles 1998; Michat and Torres 2013).

*Brachyvatus acuminatus* (Steinheil, 1869)

Figs 7G, H

*Hyphydrus acuminatus* Steinheil, 1869: 249.

*Material examined:* **Laguna Iberá RS:** 28°32’49”S 57°11’44”W, 1 spec, 14.ii.2018, light trap, coll. M. Michat; 28°33’21”S 57°11’40”W, 14 spec, 16.ii.2018, coll. M. Michat; 28°32’46”S 57°11’43”W, 1 spec, 16.ii.2018, coll. M. Michat; 28°32’49”S 57°11’48”W, 13 spec, 17.ii.2018, coll. M. Michat. **San Nicolás RS:** 28°07’43”S 57°26’09”W, 2 spec, 6.xi.2022, coll. J. Urcola.

*Distribution*: Brazil (Zimmermann 1921; Benetti et al. 2003; Benetti and Régil Cueto 2004), Argentina (provinces): Buenos Aires (Steinheil 1869; Zimmermann 1919; Fernández and López Ruf 1999; von Ellenrieder and Fernández 2000; Fernández and López Ruf 2006; Fontanarrosa et al. 2009), Chaco (Libonatti et al. 2013), Corrientes (Gómez Lutz et al. 2012; Torres et al. 2012), Entre Ríos (Torres et al. 2007; Michat and Torres 2013), Santa Fe (Macchia et al. 2015).

*Habitat:* This species has been found in small lagoons (Gómez Lutz et al. 2012), natural ponds (Libonatti et al. 2013; this study), artificial ponds (Fernández and López Ruf 2006), and slow-flowing rivers or streams (Libonatti et al. 2013), always associated with abundant aquatic vegetation. Both adults and larvae prefer environments with surfaces covered by a dense layer of floating macrophytes (mainly duckweeds), which restrict swimming (Michat and Torres 2013). Adults have also been collected using light traps (Torres et al. 2007, 2012; Libonatti et al. 2013; Macchia et al. 2015; this study).

Genus *Hemibidessus* Zimmermann, 1921

*Remarks:* This genus of small Neotropical beetles comprises six species (Nilsson and Hájek 2025), of which three are found in Argentina: *Hemibidessus bifasciatus* (Zimmermann, 1921), *Hemibidessus conicus* (Zimmermann, 1921), and *Hemibidessus spangleri* Miller, 2002.

*Hemibidessus conicus* (Zimmermann, 1921)

Figs 7I, J

*Bidessus conicus* Zimmermann, 1921: 196, 197.

*Material examined:* **Cambyretá RS:** 27°54’27”S 56°53’19”W, 11 spec, 17.xi.2018, coll. S. Mazzucconi, G. Rodriguez and J. Urcola; 27°54’10”S 56°53’14”W, 17 spec, 17.xi.2018, coll. S. Mazzucconi, G. Rodriguez and J. Urcola. **Laguna Iberá RS:** 28°32’49”S 57°11’44”W, 1 spec, 14.ii.2018, light trap, coll. M. Michat; 28°32’46”S 57°11’45”W, 1 ♀, 8.xi.2019, light trap, coll. G. Rodriguez and J. Urcola; 28°33’08”S 57°12’48”W, 1 spec, 12.xi.2019, coll. G. Rodriguez and J. Urcola. **San Nicolás RS:** 28°07’51”S 57°26’02”W, 2 spec, 4.xi.2022, coll. M. Urcola; 28°07’40”S 57°26’09”W, 1 spec, 4.xi.2022, light trap, coll. M. Urcola; 28°08’30”S 57°25’32”W, 2 spec, 5.xi.2022, coll. M. Urcola; 28°07’43”S 57°26’09”W, 27 spec, 6.xi.2022, coll. M. Urcola; 28°08’27”S 57°26’20”W, 1 spec, 8.xi.2022, coll. M. Urcola; 28°07’39”S 57°25’59”W, 6 spec, 8.xi.2022, coll. M. Urcola; 28°07’43”S 57°26’09”W, 4 spec, 9.xi.2022, coll. M. Urcola.

*Distribution:* Bolivia (Miller 2002), Brazil (Zimmermann 1921; Miller 2002), Paraguay (Zimmermann 1921; Miller 2002), Argentina (provinces): Chaco (Libonatti et al. 2013), Corrientes (Gómez Lutz et al. 2012, 2015; Torres et al. 2012; Gómez Lutz and Kehr 2017; Michat et al. 2022).

*Habitat:* This species has been found in a wide variety of habitats, such as small streams with very slow current and abundant aquatic vegetation (Miller 2002; Torres et al. 2012; Michat et al. 2022), small lagoons with abundant aquatic vegetation on the shore (Gómez Lutz et al. 2012; Michat et al. 2022), temporary ponds (Gómez Lutz and Kehr 2017; this study), roadside ditch (Michat et al. 2022), rice fields (Gómez Lutz et al. 2015), and artificial cattle water troughs (Miller 2002). It was also collected using light traps (Miller 2002; Torres et al. 2012; Libonatti et al. 2013; this study).

Genus *Liodessus* Guignot, 1939

*Remarks*: This genus of small beetles distributed across America and Africa comprises 49 valid species (Nilsson and Hájek 2025). In Argentina, the presence of nine species has been reported (Trémouilles 1998; Balke et al. 2020): *Liodessus affinis* (Say, 1823), *Liodessus bonariensis* (Steiheil, 1869), *Liodessus chilensis* (Solier, 1849), *Liodessus crassus* (Sharp, 1882), *Liodessus delfini* (Régimbart, 1899), *Liodessus flavofasciatus* (Steiheil, 1869), *Liodessus patagonicus* (Zimmermann, 1923), *Liodessus strobeli* (Steiheil, 1869), and *Liodessus uruguensis* (Sharp, 1882). In the present study, numerous specimens of an unidentified species were found. However, a morphological comparison with the material examined by Torres et al. (2012) suggests that they resemble the species mentioned in that work, which also could not be identified to the species level.

Genus *Neobidessus* Young, 1967

*Remarks:* This genus of small beetles with a Neotropical distribution, currently comprises 29 species (Nilsson and Hájek 2025). In Argentina, only two species were found: *Neobidessus curticornis* (Régimbart, 1903), and *Neobidessus grandis* Pederzani and Rocchi, 2012.

*Neobidessus grandis* Pederzani and Rocchi, 2012

Figs 7K, L

*Neobidessus grandis* Pederzani and Rocchi, 2012: 110‒112, figs 1‒5.

*Material examined:* **Laguna Iberá RS:** 28°32’46”S 57°11’45”W, 3 ♂♂ and 1 ♀, 8.xi.2019, light trap, coll. G. Rodriguez and J. Urcola.

*Distribution:* Argentina: so far only known from Corrientes Province (Pederzani and Rocchi, 2012).

*Habitat:* Currently unknown; so far, specimens have only been collected using light traps (Pederzani and Rochi 2012; this study).

*Neobidessus trilineatus* (Zimmermann, 1925)

Figs 7M, N

*Bidessus trilineatus* Zimmermann, 1925: 255, 256.

*Material examined:* **Laguna Iberá RS:** 28°32’49”S 57°11’44”W, 4 ♂♂ and 4 ♀♀, 14.ii.2018, light trap, coll. M. Michat; 28°32’49”S 57°11’44”W, 2 ♀♀, 16.ii.2018, light trap, coll. M. Michat; 28°32’46”S 57°11’45”W, 1 ♂ and 3 ♀♀, 8.xi.2019, light trap, coll. G. Rodriguez and J. Urcola. **San Nicolás RS:** 28°07’40”S 57°26’09”W, 1 spec, 5.xi.2022, light trap, coll. M. Urcola; 28°07’43”S 57°26’09”W, 1 ♂, 6.xi.2022, coll. M. Urcola; 28°07’42”S 57°26’02”W, 1 ♂, 7.xi.2022, light trap, coll. M. Urcola; 28°07’42”S 57°26’02”W, 2 ♂♂ and 1 ♀, 8.xi.2022, light trap, coll. M. Urcola; 28°07’20”S 57°25’57”W, 1 ♂, 9.xi.2022, coll. M. Urcola; 28°07’42”S 57°26’02”W, 2 ♂♂ and 3 ♀♀, 9.xi.2022, light trap, coll. M. Urcola.

*Distribution:* Brazil (Zimmermann 1925; Young 1981; Benetti et al. 2003; Benetti and Régil Cueto 2004), Argentina: Corrientes Province (**new record**).

*Habitat:* In this study, *N. trilineatus* was found in shallow ponds with turbid water and muddy bottom, exposed to high insolation and with abundant aquatic plants and filamentous algae. In addition, it was collected using light traps.

Genus *Hydrovatus* Motschulsky, 1853

*Remarks:* This cosmopolitan genus of small beetles comprises 217 species (Nilsson and Hájek 2025). In Argentina, only three species have been reported so far (Trémouilles et al. 2005): *Hydrovatus caraibus* Sharp, 1882, *Hydrovatus crassulus* Sharp, 1882, and *Hydrovatus turbinatus* Zimmermann, 1921.

*Hydrovatus turbinatus* Zimmermann, 1921

Figs 7O, P

*Hydrovatus turbinatus* Zimmermann, 1921: 191.

*Material examined:* **San Nicolás RS:** 28°07’43”S 57°26’09”W, 3 ♀♀, 6.xi.2022, coll. M. Urcola; 28°07’43”S 57°26’09”W, 1 ♂ and 1 ♀, 10.xi.2022, coll. M. Urcola.

*Distribution:* Paraguay (Trémouilles et al. 2005), Argentina (provinces): Buenos Aires (Zimmermann 1921; Trémouilles et al. 2005; Fernández and López Ruf 2006), Corrientes (Trémouilles et al. 2005; Gómez Lutz et al. 2012, 2015), Salta (Trémouilles et al. 2005).

*Habitat:* This species has been found in small lagoons (Gómez Lutz et al. 2012) and semi-permanent ponds, associated with abundant aquatic vegetation (Trémouilles et al. 2005; Fernández and López Ruf 2006; this study), and also in rice fields (Gómez Lutz et al. 2015).

Genus *Queda* Sharp, 1882

*Remarks:* This genus of small beetles, characterized by a Neotropical distribution, currently comprises three valid species (Nilsson and Hájek 2025). In Argentina, two of these species have been recorded: *Queda hydrovatoides* Zimmermann, 1921 (Michat et al. 2025), and *Queda youngi* Biström, 1990 (Libonatti et al. 2011; Torres et al. 2012).

*Queda hydrovatoides* Zimmermann, 1921

Figs 8A, B

*Queda hydrovatoides* Zimmermann, 1921: 191, 192.

*Material examined:* **Laguna Iberá RS:** 28°32’49”S 57°11’44”W, 1 spec, 5.xi.2019, coll. G. Rodriguez and J. Urcola.

*Distribution:* Brazil (Zimmermann 1921; Biström 1990; Trémouilles et al. 2004), Argentina: Corrientes Province (Michat et al. 2025).

*Habitat:* To date, the natural history of the genus *Queda* remains unknown (Biström 1990). The only specimen studied here was collected from a shallow, temporary pool adjacent to a dirt road, surrounded by a small patch of woodland vegetation.

Genus *Desmopachria* Babington, 1842

*Remarks:* This new-world genus of small diving beetles includes 155 species (Nilsson and Hájek 2025). In Argentina, 11 species have been recorded (Trémouilles 1998; Torres et al. 2008): *Desmopachria brevicollis* Régimbart, 1903, *Desmopachria chei* Miller, 1999, *Desmopachria concolor* Sharp, 1882, *Desmopachria granum* (LeConte, 1855), *Desmopachria grouvellei* Régimbart, 1895, *Desmopachria mendozana* (Steinheil, 1869), *Desmopachria nitida* Babington, 1842, *Desmopachria ovalis* Sharp, 1882, *Desmopachria punctatissima* Zimmermann, 1923, *Desmopachria subtilis* Sharp, 1882, and *Desmopachria suturalis* Sharp, 1882.

*Desmopachria concolor* Sharp, 1882

Figs 8C, D

*Desmopachria concolor* Sharp, 1882a: 340.

*Material examined:* **Laguna Iberá RS:** [28°32’47”S 57°11’45”W], 2 spec, 2.xii.2012, light trap, coll. S. Mazzucconi. **San Nicolás RS:** 28°07’51”S 57°26’02”W, 1 spec, 4.xi.2022, coll. M. Urcola; 28°07’40”S 57°26’09”W, 1 spec, 4.xi.2022, light trap, coll. M. Urcola; 28°08’17”S 57°25’49”W, 1 spec, 5.xi.2022, coll. M. Urcola; 28°07’40”S 57°26’09”W, 2 spec, 5.xi.2022, light trap, coll. M. Urcola; 28°07’43”S 57°26’09”W, 2 spec, 6.xi.2022, coll. J. Urcola; 28°10’48”S 57°26’46”W, 6 spec, 7.xi.2022, coll. M. Urcola; 28°07’42”S 57°26’02”W, 1 spec, 7.xi.2022, light trap, coll. M. Urcola; 28°07’43”S 57°26’23”W, 1 spec, 9.xi.2022, coll. M. Urcola.

*Distribution:* Brazil (Benetti et al. 2003; Benetti and Régil Cueto 2004), Paraguay (Zimmermann 1919), Uruguay (Sharp 1882a; Régimbart 1903; Benetti and Garrido 2004), Argentina (provinces): Buenos Aires (Régimbart 1903; Bruch 1915; Zimmermann 1919; Viana 1937; Fernández and López Ruf 1999, 2006; von Ellenrieder and Fernández 2000; Michat and Archangelsky 2007; Fontanarrosa et al. 2009; Macchia 2022; Macchia and Cicchino 2023), Chaco (Régimbart 1903; Libonatti et al. 2013), Corrientes (Torres et al. 2012; Gómez Lutz et al. 2012; Gómez Lutz et al. 2015; Gómez Lutz and Kehr 2017), Entre Ríos (Régimbart 1903; Torres et al. 2007), Misiones (Bruch 1915), Santa Fe (Bruch 1915; Macchia et al. 2015).

*Habitat:* This species has been found in a wide variety of habitats, including: temporary ponds (Torres et al. 2007; Fontanarrosa et al. 2009; Libonatti et al. 2013; Gómez Lutz and Kehr 2017; Macchia 2022), semi-permanent ponds (Fernández and López Ruf 2006; Torres et al. 2007; Libonatti et al. 2013; Macchia and Cicchino 2023; this study) and permanent ponds (Macchia 2022; Macchia and Cicchino 2023; this study), small lagoons with aquatic vegetation on their shores (Torres et al. 2007; Gómez Lutz et al. 2012), small streams with very slow currents and abundant aquatic vegetation (Fernández and López Ruf 2006; Torres et al. 2007, 2012; Libonatti et al. 2013; this study), artificial ponds and channels (Fernández and López Ruf 2006) and rice fields (Gómez Lutz et al. 2015). It was also collected using light traps (Torres et al. 2007, 2012; Libonatti et al. 2013; Macchia et al. 2015; this study).

*Remarks:* In this study, three additional taxa of the genus *Desmopachria* were found, but they could not be identified to the species level.

Genus *Celina* Aubé, 1837

*Remarks:* This American genus of small beetles comprises 34 species (Nilsson and Hájek 2025). In Argentina, Trémouilles (1998) mentioned the presence of seven species: *Celina angustata* Aubé, 1838, *Celina bruchi* Zimmermann, 1921, *Celina debilis* Sharp, 1882, *Celina latipes* (Brullé, 1836), *Celina mucronata* Sharp, 1882, *Celina parallela* (Babington, 1842), and *Celina punctata* Sharp, 1882.

*Celina* cf. *latipes* (Brullé, 1836)

Figs 8E, F

*Hydroporus latipes* Brullé, 1836 (in Brullé 1835‒1842): 50, pl. 4, fig. 8.

*Material examined:* **Cambyretá RS:** 27°49’00”S 56°50’00”W, 1 ♂, 16.xi.2018, light trap, coll. S. Mazzucconi, G. Rodriguez and J. Urcola. **Laguna Iberá RS:** 28°32’46”S 57°11’45”W, 6 ♂♂ and 10 ♀♀, 8.xi.2019, light trap, coll. G. Rodriguez and J. Urcola; 28°32’46”S 57°11’45”W, 14 ♂♂ and 18 ♀♀, 14.xi.2019, light trap, coll. G. Rodriguez and J. Urcola.

*Distribution:* Brazil (Aubé 1837 (in Aubé 1836–1838); Régimbart 1899b), Paraguay (Régimbart 1899b), Peru (Chaboo and Shepard 2015), Argentina: Corrientes Province (Brullé 1836 (in Brullé 1835‒1842)).

*Habitat:* Until now, unknown. The specimens studied here were collected using a light trap.

*Celina* cf. *parallela* (Babington, 1842)

Figs 8G, H

*Hydroporomorpha parallela* Babington, 1842: 15, pl. 1, fig. 3.

*Material examined:* **Laguna Iberá RS:** 28°32’46”S 57°11’45”W, 1 ♂ and 1 ♀, 8.xi.2019, light trap, coll. G. Rodriguez and J. Urcola. **San Nicolás RS:** 28°07’42”S 57°26’02”W, 1 ♂, 8.xi.2022, light trap, coll. M. Urcola; 28°07’20”S 57°25’57”W, 1 ♂ and 1 ♀, 9.xi.2022, coll. M. Urcola; 28°07’42”S 57°26’02”W, 4 ♂♂ and 2 ♀♀, 9.xi.2022, light trap, coll. M. Urcola.

*Distribution:* Brazil (Babington 1842), Argentina (provinces): Buenos Aires (Régimbart 1903), Corrientes (**new record**), Entre Ríos (Michat et al. 2007).

*Habitat*: This species has been found in shallow ponds with a muddy bottom and abundant aquatic vegetation (Michat et al. 2007; this study). It was also collected using light traps (this study).

*Remarks:* In addition to the two *Celina* species mentioned above, three other taxa of this genus were found in this study but could not be identified to the species level.

Genus *Pachydrus* Sharp, 1882

*Remarks:* This genus of small beetles comprises nine valid species (Nilsson and Hájek 2025), all with a Neotropical distribution. In Argentina, Trémouilles (1998) reported the presence of the two species found in this study.

*Pachydrus globosus* (Aubé, 1838)

Figs 8I, J

*Hyphydrus globosus* Aubé, 1838: 457.

*Material examined:* **Laguna Iberá RS:** [28°32’47”S 57°11’45”W], 1 spec, 1.xii.2012, coll. S. Mazzucconi. **Yahaveré RS:** [28°32’30”S 57°44’45”W], 1 spec, 4.ix.2015, coll. S. Mazzucconi.

*Distribution:* Brazil (Ferreira-Jr et al. 1998), Paraguay (Zimmermann 1919), Puerto Rico (Aubé 1838), Argentina (provinces): Buenos Aires (Zimmermann 1919; Fernández and López Ruf 1999, 2006; von Ellenrieder and Fernández 2000), Chaco (Libonatti et al. 2013), Córdoba (Corigliano and Raffaini 2001), Corrientes (Gómez Lutz et al. 2012, 2015; Gómez Lutz and Kehr 2017), Entre Ríos (Torres et al. 2007), Jujuy (Torres et al. 2008), Santa Fe (Macchia et al. 2015).

*Habitat:* This species has been recorded in a variety of habitats, demonstrating its adaptability to diverse environmental conditions. It inhabits small lagoons with abundantly vegetated shore, high exposure to sunlight, floating vegetation, and a high concentration of organic matter (Torres et al. 2008; Gómez Lutz et al. 2012). It is also present in small, shallow ponds with turbid water, abundant organic matter, and muddy bottom (Fernández and López Ruf 2006; Torres et al. 2007; Libonatti et al. 2013). Gómez Lutz and Kehr (2017) collected it in temporary rain-fed ponds, which provide a suitable environment during the wet season. Additionally, it has been found in slow-flowing rivers with muddy substrates and abundant aquatic vegetation (Libonatti et al. 2013), indicating a preference for stable, vegetated environments. In agricultural landscapes, it occurs in rice fields (Gómez Lutz et al. 2015), where conditions mimic those of its natural aquatic habitats. The species has also been collected using light traps (Libonatti et al. 2013; Macchia et al. 2015).

*Pachydrus obesus* Sharp, 1882

Figs 8K, L

*Pachydrus obesus* Sharp, 1882a: 339.

*Material examined:* **Cambyretá RS:** [27°49’00”S 57°11’00”W], 8 spec, 16.xi.2018, light trap, coll. S. Mazzucconi, G. Rodriguez and J. Urcola. **Laguna Iberá RS:** [28°32’47”S 57°11’45”W], 1 spec, 2.xii.2012, light trap, coll. S. Mazzucconi; 28°32’49”S 57°11’44”W, 5 spec, 14.ii.2018, light trap, coll. M. Michat; 28°33’54”S 57°12’59”W, 14 spec, 15.ii.2018, coll. M. Michat, 28°32’49”S 57°11’48”W, 22 spec, 17.ii.2018, coll. M. Michat; 28°32’46”S 57°11’45”W, 515 spec, 8.xi.2019, light trap, coll. G. Rodriguez and J. Urcola; 28°32’46”S 57°11’45”W, 237 spec, 14.xi.2019, light trap, coll. G. Rodriguez and J. Urcola.

*Distribution:* Brazil (Régimbart 1903; Zimmermann 1921; Ferreira-Jr et al. 1998), Paraguay (Régimbart 1889a; Zimmermann 1919), Venezuela (Sharp 1882a; García and Jiménez-Ramos 2020), Argentina (provinces): Buenos Aires (Bruch 1915; Fernández and López Ruf 1999; von Ellenrieder and Fernández 2000), Chaco (Libonatti et al. 2013), Corrientes (Michat and Torres 2008; Gómez Lutz et al. 2012, 2015; Torres et al. 2012), Entre Ríos (Régimbart 1903; Torres et al. 2007), Santa Fe (Macchia et al. 2015).

*Habitat:* This species has been documented in a variety of lentic habitats. It inhabits small lagoons with abundantly vegetated shore, high exposure to sunlight, floating vegetation, and a significant accumulation of organic matter (Gómez Lutz et al. 2012). It was also reported from small marshes characterized by abundant macrophytes, sandy soils, and rich organic matter (Torres et al. 2012). In agricultural landscapes, the species seems to adapt well to rice fields (Gómez Lutz et al. 2015), where moist conditions and the presence of aquatic vegetation create a suitable habitat. Additionally, it has been collected using light traps (Libonatti et al. 2013; Macchia et al. 2015; this study).

Genus *Derovatellus* Sharp, 1882

*Remarks:* This cosmopolitan genus of small beetles currently comprises 42 species (Nilsson and Hájek 2025). In Argentina, three species have been found (Miller 2005): *Derovatellus bruchi* (Zimmermann, 1919), *Derovatellus lentus* (Wehncke, 1876), and *Derovatellus spangleri* Miller, 2005.

*Derovatellus lentus* (Wehncke, 1876)

Figs 9A, B

*Vatellus lentus* Wehncke, 1876: 356‒357.

*Material examined:* **Laguna Iberá RS:** 28°32’49”S 57°11’48”W, 3 ♂♂ and 5 ♀♀, 17.ii.2018, coll. M. Michat; 28°32’46”S 57°11’45”W, 6 ♂♂ and 5 ♀♀, 8.xi.2019, light trap, coll. G. Rodriguez and J. Urcola; 28°32’46”S 57°11’45”W, 1♂ and 1♀, 14.xi.2019, light trap, coll. G. Rodriguez and J. Urcola. **San Nicolás RS:** 28°08’17”S 57°25’49”W, 1 ♀, 5.xi.2022, coll. M. Urcola.

*Distribution:* Bolivia (Miller 2005), Brazil (Zimmermann 1921; Ferreira-Jr et al. 1998; Benetti and Hamada 2003; Benetti et al. 2003; Benetti and Régil Cueto 2004; Miller 2005; Braga and Ferreira-Jr 2016a), Colombia (Miller 2005), Cuba (Megna and Sánchez-Fernández 2016), Dominica (Miller 2005), Dominican Republic (Miller 2005), Ecuador (Miller 2005), French Guiana (Régimbart 1904; Miller 2005), Guadeloupe (Manuel 2015), Guatemala (Miller 2005), Guyana (Miller 2005), Panama (Miller 2005), Paraguay (Miller 2005), Peru (Miller 2005; Chaboo and Shepard 2015), Puerto Rico (Wehncke 1876), Suriname (Miller 2005), Trinidad (Miller 2005), Venezuela (Miller 2005), Argentina (provinces): Buenos Aires (Fernández and López Ruf 1999), Chaco (Michat and Torres 2011; Libonatti et al. 2013), Corrientes (Miller 2005; Torres et al. 2012; Michat et al. 2024), Entre Ríos (Torres et al. 2007), Santa Fe (Miller 2005; Macchia et al. 2015), Tucumán (Miller 2005).

*Habitat:* This species has been found in a wide variety of habitats, including small shallow lagoons with high exposure to sunlight and abundant vegetation and organic matter (Torres et al. 2012), as well as in semi-permanent ponds with vegetated margins (Miller 2005; Torres et al. 2007; Michat and Torres 2011; Libonatti et al. 2013) and temporary pools (Benetti and Hamada 2003; Benetti and Régil Cueto 2004; Miller 2005; Torres et al. 2007; this study). Additionally, it has been reported from small streams with very slow current, completely covered by aquatic vegetation (Benetti and Hamada 2003; Libonatti et al. 2013; this study). It has been also collected using light traps (Miller 2005; Torres et al. 2012; Libonatti et al. 2013; Macchia et al. 2015; this study).

Genus *Vatellus* Aubé, 1837

*Remarks:* This American genus of small beetles comprises 17 valid species (Nilsson and Hájek 2025). Up to now, only the two species mentioned below have been found in Argentina (Miller 2005).

*Vatellus haagi* Wehncke, 1876

Figs 9C, D

*Vatellus haagi* Wehncke, 1876: 357.

*Material examined:* **Laguna Iberá RS:** [28°32’47”S 57°11’45”W], 1 ♀, 2.xii.2012, light trap, coll. S. Mazzucconi.

*Distribution:* Bolivia (Régimbart 1899a; Miller 2005), Brazil (Wehncke 1876), Paraguay (Miller 2005), Uruguay (Benetti and Garrido 2004; Miller 2005), Argentina (provinces): Buenos Aires (Régimbart 1903; Bruch 1915; Zimmermann 1919; von Ellenrieder and Fernández 2000; Michat and Torres 2005; Miller 2005; Fontanarrosa et al. 2009; Macchia and Cicchino 2023), Chaco (Régimbart 1903; Miller 2005; Libonatti et al. 2013), Córdoba (Corigliano and Raffaini 2001; Miller 2005), Corrientes (Bruch 1915; Torres et al. 2012), Entre Ríos (Régimbart 1903; Michat and Torres 2005; Miller 2005; Torres et al. 2007), Formosa (Miller 2005), Misiones (Bruch 1915; Miller 2005), Santa Fe (Bruch 1915; Miller 2005; Macchia et al. 2015).

*Habitat:* This species inhabits a variety of freshwater environments, including shallow lagoons (Torres et al. 2007), temporary ponds (Miller 2005; Torres et al. 2007; Fontanarrosa et al. 2009; Macchia and Cicchino 2023), and small streams with very slow current (Libonatti et al. 2013). These habitats are characterized by muddy substrates and are often completely covered with dense vegetation. Additionally, individuals of this species have been frequently collected using light traps (Miller 2005; Torres et al. 2012; Libonatti et al. 2013; Macchia et al. 2015; this study).

*Vatellus wheeleri* Miller, 2005

Figs 9E, F

*Vatellus wheeleri* Miller, 2005: 473‒475, figs 189‒202.

*Material examined:* **Laguna Iberá RS:** 28°32’46”S 57°11’45”W, 1 ♂ and 1 ♀, 8.xi.2019, light trap, coll. G. Rodriguez and J. Urcola; 28°32’46”S 57°11’45”W, 1 ♂, 14.xi.2019, light trap, coll. G. Rodriguez and J. Urcola.

*Distribution:* Paraguay (Miller 2005), Argentina (provinces): Buenos Aires (Macchia 2022; Macchia and Cicchino 2023), Chaco (Miller 2005), Corrientes (Miller 2005; Torres et al. 2012).

*Habitat:* This species can be found in lentic environments, including small lagoons (Torres et al. 2012) and temporary ponds (Macchia 2022; Macchia and Cicchino 2023). Additionally, specimens have been collected using light traps (Miller 2005; Torres et al. 2012; this study).

Subfamily Laccophilinae Gistel, 1848

Genus *Laccomimus* Toledo and Michat, 2015

*Remarks:* This American genus of very small beetles currently comprises 13 species (Nilsson and Hájek 2025), three of which have been mentioned as present in Argentina: *Laccomimus alvarengi* Toledo and Michat, 2015, *Laccomimus bordoni* Toledo and Michat, 2015, and *Laccomimus distinctus* Toledo and Michat, 2015.

*Laccomimus alvarengi* Toledo and Michat, 2015

Figs 9G, H

*Laccomimus alvarengi* Toledo and Michat, 2015: 317‒319, figs 3, 5, 11, 14, 15, 28, 39, 57, 64, 69, 77‒81, 94‒102, 111, 118.

*Material examined:* **Laguna Iberá RS:** 28°32’46”S 57°11’45”W, 14 spec, 8.xi.2019, light trap, coll. G. Rodriguez and J. Urcola; 28°32’46”S 57°11’45”W, 26 spec, 14.xi.2019, light trap, coll. G. Rodriguez and J. Urcola. **San Nicolás RS:** 28°07’51”S 57°26’02”W, 1 ♀, 4.xi.2022, coll. J. Urcola.

*Distribution:* Bolivia (Toledo and Michat 2015), Brazil (Toledo and Michat 2015; Braga and Ferreira-Jr 2016b; Manuel 2017), Ecuador (Toledo and Michat 2015), Panama (Toledo and Michat 2015), Paraguay (Toledo and Michat 2015), Peru (Toledo and Michat 2015), Suriname (Toledo and Michat 2015), Argentina: Corrientes Province (Toledo and Michat 2015).

*Habitat:* This species has been found in a series of interconnected shallow ponds characterized by muddy bottom and abundant floating and submerged vegetation (this study). Specimens were also collected using light traps (Toledo and Michat 2015; this study).

Genus *Laccophilus* Leach, 1815

*Remarks:* This cosmopolitan genus of small diving beetles comprises 295 valid species (Nilsson and Hájek 2025). In Argentina, Trémouilles (1998) lists the presence of 12 species: *Laccophilus angustus* Régimbart, 1889, *Laccophilus balzani* Régimbart, 1889, *Laccophilus chelinus* Guignot, 1955, *Laccophilus gounellei* Régimbart, 1903, *Laccophilus latipes* Sharp, 1882, *Laccophilus nigricans* Sharp, 1882, *Laccophilus notatus* Boheman, 1858, *Laccophilus nubilus* Régimbart, 1889, *Laccophilus obliquatus* Régimbart, 1889, *Laccophilus paraguensis* Régimbart, 1903, *Laccophilus tarsalis* Sharp, 1882, and *Laccophilus testudo* Régimbart, 1903. This study recorded four species, two of which could be identified to the species level, as detailed below.

*Laccophilus* cf. *obliquatus* Régimbart, 1889

Figs 9I, J

*Laccophilus obliquatus* Régimbart, 1889a: 264, 265.

*Material examined:* **Laguna Iberá RS:** 28°34’13”S 57°10’13”W, 1 spec, 16.ii.2018, coll. M. Michat; 28°32’46”S 57°11’45”W, 230 spec, 8.xi.2019, light trap, coll. G. Rodriguez and J. Urcola. **San Nicolás RS:** 28°07’51”S 57°26’02”W, 3 ♂♂ and 1 ♀, 4.xi.2022, coll. M. Urcola; 28°08’17”S 57°25’49”W, 1 ♂ and 3 ♀♀, 5.xi.2022, coll. M. Urcola; 28°07’43”S 57°26’09”W, 2 ♂♂ and 2 ♀♀, 6.xi.2022, coll. M. Urcola; 28°10’48”S 57°26’46”W, 52 spec, 7.xi.2022, coll. M. Urcola; 28°09’25”S 57°26’34”W, 1 ♂ and 3 ♀♀, 8.xi.2022, coll. M. Urcola; 28°07’39”S 57°25’59”W, 34 spec, 8.xi.2022, coll. M. Urcola; 28°07’42”S 57°26’02”W, 1 ♀, 8.xi.2022, light trap, coll. M. Urcola; 28°07’20”S 57°25’57”W, 3 ♂♂ and 3 ♀♀, 9.xi.2022, coll. M. Urcola; 28°07’42”S 57°26’02”W, 1 ♀, 9.xi.2022, light trap, coll. M. Urcola.

*Distribution:* Brazil (Régimbart 1889a; Benetti et al. 2003), Paraguay (Régimbart 1889a), Argentina (provinces): Buenos Aires (Michat 2008; Macchia and Cicchino 2023), Corrientes (**new record**).

*Habitat:* This species inhabits a wide range of environments, including shallow lagoons (Macchia and Cicchino 2023; this study), temporary ponds (Michat 2008; Macchia and Cicchino 2023; this study), and small streams with very slow current (Macchia and Cicchino 2023; this study). These habitats are all characterized by muddy bottom and abundant aquatic vegetation. Specimens were also collected using light traps (this study).

*Laccophilus* cf. *paraguensis* Régimbart, 1903

Figs 9K, L

*Laccophilus paraguensis* Régimbart, 1903: 68.

*Material examined:* **San Nicolás RS:** 28°07’51”S 57°26’02”W, 1 ♂, 4.xi.2022, coll. M. Urcola; 28°07’40”S 57°26’09”W, 1 ♂, 4.xi.2022, light trap, coll. M. Urcola; 28°08’17”S 57°25’49”W, 1 ♀, 5.xi.2022, coll. M. Urcola; 28°10’48”S 57°26’46”W, 2 ♀♀, 7.xi.2022, coll. M. Urcola; 28°07’42”S 57°26’02”W, 1 ♂ and 1 ♀, 7.xi.2022, light trap, coll. M. Urcola; 28°07’42”S 57°26’02”W, 1 ♂, 8.xi.2022, light trap, coll. M. Urcola; 28°07’20”S 57°25’57”W, 2 ♀♀, 9.xi.2022, coll. M. Urcola; 28°07’42”S 57°26’02”W, 1 ♂, 9.xi.2022, light trap, coll. M. Urcola.

*Distribution:* Brazil (Benetti et al. 2003), Paraguay (Régimbart 1903), Argentina (provinces): Buenos Aires (Régimbart 1903; Michat 2008; Macchia and Cicchino 2023), Chaco (Régimbart 1903), Corrientes (**new record**), Entre Ríos (Régimbart 1903).

*Habitat:* This species inhabits both lentic and lotic environments, including small lagoons (this study), temporary ponds (Michat 2008; Macchia and Cicchino 2023), and small streams with very slow current (Macchia and Cicchino 2023; this study). Specimens were also collected using light traps (this study).

*Remarks:* The specimen figured by Macchia (2022) as *L. paraguensis* (fig. 5D in that paper) corresponds to *L. obliquatus*. In our study, in addition to the two *Laccophilus* species mentioned above, two other taxa were recorded but could not be identified to the species level.

Family Gyrinidae Latreille, 1810

Subfamily Gyrininae Latreille, 1810

Genus *Gyretes* Brullé, 1835

*Remarks:* This American genus of small beetles includes 204 species. In Argentina, the occurrence of 19 species has been reported (Michat and Archangelsky 2014). In this study, two unidentified species of *Gyretes* were found. Unfortunately, the uniformity of the morphological traits exhibited by the species of this genus, coupled with the fact that existing taxonomic keys in the literature only partially differentiate a limited number of species outside Argentina, hindered their identification at the species level.

Genus *Gyrinus* Geoffroy, 1762

*Remarks:* This cosmopolitan genus of small to medium sized beetles currently comprises 138 valid species, of which six have been mentioned for Argentina (Michat and Archangelsky 2014): *Gyrinus argentinus* Steinheil, 1869, *Gyrinus chalybeus* Perty, 1830, *Gyrinus gibbus* Aubé, 1838, *Gyrinus monrosi* Mouchamps, 1957, *Gyrinus ovatus* Aubé, 1838, and *Gyrinus violaceus* Régimbart, 1883.

*Gyrinus violaceus* Régimbart, 1883

Figs 10A, B

*Gyrinus violaceus* Régimbart, 1883: 187, 188, pl. 6, fig. 94.

*Material examined:* **Laguna Iberá RS:** 28°32’46”S 57°11’43”W, 2 ♂♂ and 3 ♀♀, 16.ii.2018, coll. M. Michat; 28°32’46”S 57°11’45”W, 2 ♂♂, 8.xi.2019, light trap, coll. G. Rodriguez and J. Urcola. **San Nicolás RS:** 28°07’43”S 57°26’09”W, 4 ♂♂ and 3 ♀♀, 6.xi.2022, coll. M. Urcola; 28°10’48”S 57°26’46”W, 16 ♂♂ and 25 ♀♀, 7.xi.2022, coll. M. Urcola; 28°09’25”S 57°26’34”W, 22 ♂♂ and 30 ♀♀, 8.xi.2022, coll. M. Urcola.

*Distribution:* Brazil (Benetti et al. 2003; Benetti and Régil Cueto 2004), Uruguay (Régimbart 1883; Ochs 1956), Argentina (provinces): Chaco (Libonatti et al. 2013), Corrientes (Torres et al. 2012), Misiones (Bruch 1915; Fernández et al. 2008).

*Habitat:* This species inhabits a variety of aquatic environments, both lentic and lotic. It has been documented in small, very slow-flowing streams characterized by high exposure to sunlight, muddy bottom, and dense aquatic vegetation (Benetti and Régil Cueto 2004; Torres et al. 2012; Libonatti et al. 2013). It has also been observed in shallow ponds with high exposure to sunlight, high concentration of organic matter, muddy bottom, and an almost complete vegetation cover (this study). Additionally, it has been found in small, shallow pools that are fully exposed to sunlight, with turbid water, muddy bottom, and abundant marginal vegetation (Libonatti et al. 2013; this study). Some individuals of this species have also been captured using light traps (Torres et al. 2012; Libonatti et al. 2013; this study).

Family Haliplidae Aubé, 1836

Genus *Haliplus* Latreille, 1802

*Remarks:* This cosmopolitan genus of small beetles comprises 186 species (Bánki et al. 2024). In Argentina, Archangelsky and Michat (2014) reported the presence of 10 species: *Haliplus bachmanni* Vidal Sarmiento and Grosso, 1970, *Haliplus bonariensis* Steinheil, 1869, *Haliplus drechseli* van Vondel and Spangler, 2008, *Haliplus gravidus* Aubé, 1838, *Haliplus indistinctus* Zimmermann, 1928, *Haliplus maculicollis* Zimmermann, 1924, *Haliplus oblongus* Zimmermann, 1921, *Haliplus ornatipennis* Zimmermann, 1921, *Haliplus subseriatus* Zimmermann, 1921, and *Haliplus testaceus* Zimmermann, 1924.

*Haliplus nieseri* van Vondel and Spangler, 2008

Figs 10C, D

*Haliplus nieseri* van Vondel and Spangler, 2008: 107, 162, 186, figs 285‒293, 498.

*Material examined:* **San Nicolás RS:** 28°07’39”S 57°25’59”W, 1 ♂ and 1 ♀, 8.xi.2022, coll. J. Urcola.

*Distribution:* Brazil (van Vondel and Spangler 2008), Argentina: Corrientes Province (**new record**).

*Habitat:* van Vondel and Spangler (2008) collected this species in a sandy pool and with a light trap. In this study, the two specimens examined were found in a small pool fully exposed to sunlight, with very turbid water, muddy bottom, and high concentration of filamentous algae.

*Haliplus ornatipennis* Zimmermann, 1921

Figs 10E, F

*Haliplus ornatipennis* Zimmermann, 1921: 182, 183.

*Material examined:* **Laguna Iberá RS:** 28°33’54”S 57°12’59”W, 3 ♂♂ and 2 ♀♀, 15.ii.2018, coll. M. Michat. **San Nicolás RS:** 28°08’17”S 57°25’49”W, 1 ♀, 5.xi.2022, coll. J. Urcola.

*Distribution:* Bolivia (van Vondel and Spangler 2008; van Vondel 2016), Brazil (van Vondel and Spangler 2008), Paraguay (van Vondel and Spangler 2008), Peru (van Vondel and Spangler 2008; Chaboo and Shepard 2015), Argentina (provinces): Buenos Aires (Vidal Sarmiento and Grosso 1971), Córdoba (van Vondel and Spangler 2008; van Vondel 2016), Corrientes (**new record**), Formosa (Vidal Sarmiento and Grosso 1971; van Vondel and Spangler 2008), La Rioja (Vidal Sarmiento and Grosso 1971; van Vondel and Spangler 2008), Salta (Vidal Sarmiento and Grosso 1971; van Vondel and Spangler 2008), Santa Fe (van Vondel and Spangler 2008), Santiago del Estero (Vidal Sarmiento and Grosso 1971; van Vondel and Spangler 2008), Tucumán (Zimmermann 1921; Vidal Sarmiento and Grosso 1971; van Vondel and Spangler 2008).

*Habitat:* van Vondel and Spangler (2008) collected this species in ponds and pools, as well as using light traps. In this study, specimens were found in a temporary puddle next to a dirt road, covered with dense aquatic vegetation, as well as in a small stream with very slow current, featuring abundant grasses.

Family Noteridae Thomson, 1860

Subfamily Noterinae Thomson, 1860

Genus *Hydrocanthus* Say, 1823

*Remarks: Hydrocanthus* is endemic to America (Baca et al. 2017) and includes 17 species of medium to large-sized noterids (total length about 3.4–5.2 mm) (Nilsson 2011; Guimarães et al. 2018). In Argentina, six species have been recorded: *Hydrocanthus debilis* Sharp, 1882, *Hydrocanthus levigatus* (Brulle, 1837), *Hydrocanthus fasciatus* Steinheil, 1869, *Hydrocanthus paraguayensis* Zimmermann, 1928, *Hydrocanthus sharpi* Zimmermann, 1928, and *Hydrocanthus socius* Sahlberg, 1844. In this study, in addition to the species detailed below, a male specimen was collected that could not be assigned to any of them, suggesting that it may represent a species not yet recorded in Argentina or even a new species.

*Hydrocanthus debilis* Sharp, 1882

Figs 11A, B

*Hydrocanthus debilis* Sharp, 1882a: 281.

*Material examined:* **Galarza RS:** [28°06’23”S 56°44’11”W], 1 ♀, 17.iv.2013, coll. S. Mazzucconi. **Cambyretá RS:** 27°49’27”S 56°50’51”W, 7 ♂♂ and 12 ♀♀, 16.xi.2018, light trap, coll. J. Urcola. **Itatí RS:** [28°44’27”S 58°07’46”W], 26.ix.2003, 1 ♀, coll. S. Mazzucconi. **Laguna Iberá RS:** [28°32’47”S 57°11’45”W], 2.xii.2012, 1 ♂, coll. S. Mazzucconi; 28°32’47”S 57°11’44”W, 2 ♂♂, 14.ii.2018, coll. J. Urcola; 28°32’49”S 57°11’48”W, 1 ♀, 14.ii.2018, light trap, coll. J. Urcola; 28°33’54”S 57°12’59”W, 4 ♂♂ and 5 ♀♀, 15.ii.2018, coll. J. Urcola; 28°33’21”S 57°11’40”W, 1 ♂ and 2 ♀♀, 16.ii.2018, coll. J. Urcola; 28°32’48”S 57°11’48”W, 1 ♀, 17.ii.2018, coll. J. Urcola; 28°32’48”S 57°11’48”W, 2 ♂♂ and 11 ♀♀, 5.xi.2019, coll. J. Urcola; 28°32’48”S

57°11’48”W, 1 ♀, 5.xi.2019, coll. J. Urcola; 28°32’46”S 57°12’05”W, 1 ♂ and 1 ♀, 6.xi.2019, coll. J. Urcola; 28°32’46”S 57°11’05”W, 1 spec, 6.xi.2019, coll. J. Urcola; 28°33’21”S 57°11’40”W, 43 spec, 7.xi.2019, coll. J. Urcola; 28°32’46”S 57°11’45”W, 57 spec, 7.xi.2019, light trap, coll. J. Urcola; 28°32’46”S 57°11’45”W, 55 spec, 8.xi.2019, light trap, coll. J. Urcola; 28°32’48”S 57°11’48”W, 3 ♀♀, 9.xi.2019, coll. J. Urcola; 28°32’51”S 57°11’53”W, 6 ♂♂ and 12 ♀♀, 9.xi.2019, coll. J. Urcola; 28°33’54”S 57°12’59”W, 1 ♂ and 2 ♀♀, 10.xi.2019, coll. J. Urcola; 28°32’46”S 57°11’45”W, 1 ♂ and 1 ♀, 10.xi.2019, light trap, coll. J. Urcola; 28°33’01”S 57°12’45”W, 1 ♂ and 8 ♀♀, 11.xi.2019, coll. J. Urcola; 28°33’08”S 57°12’48”W, 3 ♂♂ and 8 ♀♀, 11.xi.2019, coll. J. Urcola; 28°33’17”S 57°12’54”W, 3 ♂♂ and 1 ♀, 12.xi.2019, coll. J. Urcola; 28°33’08”S 57°12’48”W, 36 spec, 12.xi.2019, coll. J. Urcola; 28°32’46”S 57°11’45”W, 4 ♀♀, 12.xi.2019, light trap, coll. J. Urcola; 28°32’56”S 57°11’58”W, 2 ♂♂ and 1 ♀, 14.xi.2019, coll. J. Urcola; 28°32’56”S 57°11’58”W, 2 ♂♂ and 5 ♀♀, 14.xi.2019, coll. J. Urcola; 28°32’46”S 57°11’45”W, 318 spec, 14.xi.2019, light trap, coll. J. Urcola. **San Nicolás RS:** 28°07’43”S 57°26’09”W, 1 ♂, 6.xi.2022, coll. J. Urcola; 28°09’25”S 57°26’34”W, 1 ♂ and 1 ♀, 8.xi.2022, coll. J. Urcola.

*Distribution:* Belize (Scheers and Thomaes 2017), Bolivia (Grosso 1979), Brazil (Sharp 1882a, b; Zimmermann 1921; Ferreira-Jr et al. 1998; Benetti et al. 2003; Benetti and Hamada 2003; Benetti and Régil Cueto 2004; Guimarães et al. 2018), Costa Rica (Blanco Aller 2015), French Guiana (Guimarães et al. 2018), Guatemala (Sharp 1882b), Mexico (Sharp 1882b; Young 1985), Panama (Sharp 1882b), Paraguay (Zimmermann 1919; Grosso 1979), Peru (Chaboo and Shepard 2015), Uruguay (Benetti and Garrido 2004), Argentina (provinces): Buenos Aires (Régimbart 1903; Bruch 1915; Viana 1937; Grosso 1979; Fernández and López Ruf 1999; Fernández and López Ruf 2006), Chaco (Régimbart 1889a; Grosso 1979; Libonatti et al. 2013), Corrientes (Régimbart 1903; Bruch 1915; Grosso 1979; Torres et al. 2012; Urcola et al. 2022), Entre Ríos (Grosso 1979), Formosa (Grosso 1979), Misiones (Bruch 1915; Grosso 1979), Salta (Grosso 1979), Santa Fe (Grosso 1979; Macchia et al. 2015), Tucumán (Grosso 1979).

*Habitat:* The species is common in lagoons, marshes and ponds of various sizes, always associated with abundant aquatic vegetation. It also occurs in vegetated parts of streams with very slow current (Torres et al. 2012; Libonatti et al. 2013; this study). Urcola et al. (2022: fig. 24) found this species in a shallow puddle, completely exposed to sunlight, with a muddy bottom and abundant emergent vegetation, mainly grasses. It is also frequently collected in light traps (Macchia et al. 2015; this study).

*Hydrocanthus levigatus* (Brullé, 1837)

Figs 11C, D

*Noterus levigatus* Brullé, 1837 (in Brullé 1835‒1842): 50.

*Material examined:* **Laguna Iberá RS:** 28°32’49”S 57°11’48”W, 49 spec, 14.ii.2018, light trap, coll. J. Urcola; 28°33’21”S 57°11’40”W, 1 ♀, 7.xi.2019, coll. J. Urcola; 28°33’17”S 57°12’54”W, 1 ♀, 7.xi.2019, coll. J. Urcola; 28°32’46”S 57°11’45”W, 39 spec, 14.xi.2019, light trap, coll. J. Urcola. **San Nicolás RS:** 28°08’30”S 57°25’32”W, 1 ♂, 5.xi.2022, coll. J. Urcola; 28°08’17”S 57°25’49”W, 1 ♀, 5.xi.2022, coll. J. Urcola; 28°07’43”S 57°26’09”W, 2 ♂♂ and 2 ♀♀, 6.xi.2022, coll. J. Urcola; 28°10’48”S 57°26’46”W, 1 ♂, 7.xi.2022, coll. J. Urcola; 28°07’39”S 57°25’59”W, 2 ♂♂, 8.xi.2022, coll. J. Urcola; 28°07’20”S 57°25’57”W, 1 ♀, 9.xi.2022, coll. J. Urcola; 28°07’42”S 57°26’02”W, 1 ♀, 9.xi.2022, light trap, coll. J. Urcola.

*Distribution:* Bolivia (Grosso 1979), Brazil (Sharp 1882a; Régimbart 1903; Zimmermann 1919), Guadeloupe (Zimmermann 1919), Panama (Sharp 1882b), Paraguay (Zimmermann 1919; Grosso 1979), Venezuela (Sharp 1882a), Argentina (provinces): Buenos Aires (Grosso 1979; von Ellenrieder and Fernández 2000; Fernández and López Ruf 2006), Catamarca (Grosso 1979), Chaco (Régimbart 1903; Grosso 1979; Libonatti et al. 2013), Corrientes (Grosso 1979; Torres et al. 2012; Urcola et al. 2024a), Entre Ríos (Régimbart 1903; Grosso 1979), Formosa (Grosso 1979), Misiones (Grosso 1979), Salta (Grosso 1979), Santa Fe (Grosso 1979; Macchia et al. 2015), Tucumán (Grosso 1979).

*Habitat:* This species has been found in ponds that were mostly exposed to sunlight, with a muddy bottom, relatively high turbidity, and abundant emergent vegetation (Torres et al. 2012; Libonatti et al. 2013; Urcola et al. 2024a; this study). It was also recorded in small streams with very slow current, covered with vegetation (Libonatti et al. 2013; this study). Additionally, it was collected using light traps (Torres et al. 2012; Macchia et al. 2015; this study).

*Hydrocanthus paraguayensis* Zimmermann, 1928

Figs 11E, F

*Hydrocanthus paraguayensis* Zimmermann, 1928: 166, 167.

*Material examined:* **Laguna Iberá RS:** [28°32’46”S 57°11’44”W], 1 ♀, 15.iv.2014, coll. S. Mazzucconi; 28°33’21”S 57°11’40”W, 1 ♀, 16.ii.2018, coll. J. Urcola.

*Distribution:* Bolivia (Guimarães et al. 2018), Brazil (Benetti et al. 2003; Benetti and Régil Cueto 2004), Paraguay (Zimmermann 1928; Guimarães et al. 2018), Uruguay (Young 1985), Argentina (provinces): Buenos Aires (Fernández and López Ruf 1999; von Ellenrieder and Fernández 2000), Corrientes (**new record**).

*Habitat:* In this study, specimens of *H. paraguayensis* were collected in a semi-permanent pond near Laguna Iberá.

*Remarks:* Several previous records of this species (Grosso 1979; Torres et al. 2012; Libonatti et al. 2013; Macchia et al. 2015) correspond to *H. levigatus* (Urcola et al. 2024a), and were therefore included in the distribution of the latter species (see above).

*Hydrocanthus sharpi* Zimmermann, 1928

Figs 11G, H

*Hydrocanthus sharpi* Zimmermann, 1928: 169.

*Material examined:* **Itatí RS:** [28°44’27”S 58°07’46”W], 1 ♂, 26.ix.2003, coll. S. Mazzucconi. **Laguna Iberá RS:** 28°33’54”S 57°12’59”W, 1 ♂ and 3 ♀♀, 15.ii.2018, coll. J. Urcola; 28°33’21”S 57°11’40”W, 1 ♂ and 1 ♀, 16.ii.2018, coll. J. Urcola; 28°32’48” S 57°11’48”W, 1 ♂ and 3 ♀♀, 17.ii.2018, coll. J. Urcola; 28°33’17”S 57°12’54”W, 1 ♀, 12.xi.2019, coll. J. Urcola. **San Nicolás RS:** 28°07’43”S 57°26’09”W, 3 ♂♂, 5.xi.2022, coll. J. Urcola; 28°07’43”S 57°26’09”W, 3 ♂♂, 6.xi.2022, coll. J. Urcola; 28°07’43”S 57°26’09”W, 1 ♂ and 2 ♀♀, 10.xi.2022, coll. J. Urcola.

*Distribution:* Brazil (Benetti et al. 2003; Benetti and Régil Cueto 2004), Ecuador (Young 1985), Argentina (provinces): Buenos Aires (Grosso 1979; Fernández and López Ruf 1999; Fernández and López Ruf 2006; Urcola et al. 2019a), Chaco (Grosso 1979; Libonatti et al. 2013), Corrientes (Gómez Lutz et al. 2012; Torres et al. 2012), Entre Ríos (Grosso 1979), Formosa (Grosso 1979), Salta (Grosso 1979).

*Habitat:* This species has been found in shallow ponds exposed to high insolation, with muddy bottom and abundant aquatic vegetation (Benetti and Régil Cueto 2004; Fernández and López Ruf 2006; Torres et al. 2012; Urcola et al. 2019a; this study). It was also collected from a slow-flowing stream with high exposure to sunlight, muddy bottom, and a surface completely covered with aquatic vegetation (Libonatti et al. 2013). Gómez Lutz et al. (2012) found the species in two small, shallow lagoons in the northern part of Corrientes province. Torres et al. (2012) collected specimens of *H. sharpi* using light traps.

*Hydrocanthus socius* Sahlberg, 1844

Figs 11I, J

*Hydrocanthus socius* Sahlberg, 1844: 516.

*Material examined:* **Cambyretá RS:** 27°49’27”S 56°50’51”W, 3545 spec, 16.xi.2018, light trap, coll. J. Urcola; 27°54’10”S 56°53’14”W, 43 spec, 17.xi.2018, coll. J. Urcola; 27°54’00”S 56°53’06”W, 7 ♂♂ and 13 ♀♀, 17.xi.2018, coll. J. Urcola; 27°52’11”S 56°52’59”W, 1 ♂ and 1♀, 18.xi.2018, coll. J. Urcola; 27°52’11”S 56°52’59”W, 1 ♂ and 1♀, 18.xi.2018, coll. J. Urcola; 27°52’15”S 56°53’00”W, 2 ♂♂ and 3 ♀♀, 18.xi.2018, coll. J. Urcola; 27°52’22”S 56°52’57”W, 3 ♀♀, 19.xi.2018, coll. J. Urcola; 27°52’26”S 56°52’49”W, 1 ♀, 19.xi.2018, coll. J. Urcola. **Galarza RS:** [28°04’54”S 56°42’07”W], 1 ♀, 16.iv.2013, coll. S. Mazzucconi; [28°04’50”S 56°44’16”W], 1 ♂, 19.iv.2013, coll. S. Mazzucconi. **Laguna Iberá RS:** [28°32’47”S 57°11’45”W], 171 spec, 2.xii.2012, light trap, coll. S. Mazzucconi; 28°31’37”S 57°8’46”W, 1 ♂, 3.xii.2012, coll. S. Mazzucconi; 28°32’49”S 57°11’48”W, 4 ♂♂ and 8 ♀♀, 14.ii.2018, coll. J. Urcola; 28°33’54”S 57°12’59”W, 1 ♂, 15.ii.2018, coll. J. Urcola; 28°33’21”S 57°11’40”W, 1 ♀, 16.ii.2018, coll. J. Urcola; 28°32’49”S 57°11’48”W, 1 ♂ and 2 ♀, 16.ii.2018, light trap, coll. J. Urcola; 28°32’48”S 57°11’48”W, 4 ♀♀, 5.xi.2019, coll. J. Urcola; 28°32’46”S 57°11’45”W, 1 ♂ and 4 ♀♀, 7.xi.2019, light trap, coll. J. Urcola; 28°32’46”S 57°11’45”W, 1 ♂ and 10 ♀♀, 8.xi.2019, light trap, coll. J. Urcola; 28°32’48”S 57°11’48”W, 2 ♀♀, 9.xi.2019, coll. J. Urcola; 28°33’54”S 57°12’59”W, 3 ♂♂ and 8 ♀♀, 10.xi.2019, coll. J. Urcola; 28°33’08”S 57°12’48”W, 1 ♀, 11.xi.2019, coll. J. Urcola; 28°33’08”S 57°12’48”W, 2 ♀♀, 12.xi.2019, coll. J. Urcola; 28°32’46”S 57°11’45”W, 2 ♂♂ and 3 ♀♀, 12.xi.2019, light trap, coll. J. Urcola; 28°32’46”S 57°11’45”W, 84 spec, 14.xi.2019, light trap, coll. J. Urcola. **San Nicolás RS:** 28°07’51”S 57°26’02”W, 2 ♂♂ and 5 ♀♀, 4.xi.2022, coll. J. Urcola; 28°08’17”S 57°25’49”W, 1 ♀, 5.xi.2022, coll. J. Urcola; 28°07’43”S 57°26’09”W, 1 ♂, 6.xi.2022, coll. J. Urcola; 28°09’25”S 57°26’34”W, 1 ♂, 8.xi.2022, coll. J. Urcola; 28°07’43”S 57°26’09”W, 1 ♀, 9.xi.2022, coll. J. Urcola.

*Distribution:* Bolivia (Grosso 1979), Brazil (Zimmermann 1921; Young 1985; Benetti et al. 2003; Benetti and Régil Cueto 2004), Paraguay (Régimbart 1903; Grosso 1979), Peru (Chaboo and Shepard 2015), Venezuela (Régimbart 1903), Argentina (provinces): Chaco (Libonatti et al. 2013), Corrientes (Torres et al. 2012), Entre Ríos (Grosso 1979), Formosa (Grosso 1979).

*Habitat:* This species has been found in various habitats (e.g., marshes, lagoons, pools, and streams), all characterized by high exposure to sunlight, muddy substrate, and abundant aquatic vegetation (Benetti and Régil Cueto 2004; Torres et al. 2012; this study). It was also collected using light traps (Torres et al. 2012; Libonatti et al. 2013; this study).

Genus *Mesonoterus* Sharp, 1882

*Remarks:* This American genus of small beetles comprises 11 valid species (García Ramírez 2019). In Argentina, *Mesonoterus crassicornis* (Régimbart, 1889), and *Mesonoterus laevicollis* Sharp, 1882 have been recorded (Urcola and Michat 2023b).

*Mesonoterus crassicornis* (Régimbart, 1889)

Figs 11K, L

*Canthydrus crassicornis* Régimbart, 1889a: 258, 259.

*Material examined:* **Cambyretá RS:** 27°49’27”S 56°50’51”W, 63 ♂♂ and 98 ♀♀, 16.xi.2018, light trap, coll. J. Urcola; 27°54’00”S 56°53’06”W, 2 ♂♂, 17.xi.2018, coll. J. Urcola; 27°52’00”S 56°52’00”W, 1 ♀, 18.xi.2018, coll. J. Urcola. **Laguna Iberá RS:** 28°33’54”S 57°12’59”W, 1 ♂, 15.ii.2018, coll. J. Urcola; 28°33’21”S 57°11’40”W, 1 ♂, 16.ii.2018, coll. J. Urcola; 28°32’46”S 57°11’45”W, 1 ♂ and 9 ♂♂, 12.xi.2019, light trap, coll. J. Urcola; 28°32’46”S 57°11’45”W, 217 spec, 14.xi.2019, light trap, coll. J. Urcola.

*Distribution:* Brazil (Régimbart 1889a), Paraguay (Régimbart 1889a), Argentina (provinces): Corrientes (**new record**), Formosa (Grosso 1979).

*Habitat:* In this study, specimens of *M. crassicornis* were found in semi-permanent and temporary ponds with high exposure to sunlight and characterized by abundant aquatic vegetation. They were also collected using light traps.

*Mesonoterus laevicollis* Sharp, 1882

Figs 11M, N

*Mesonoterus laevicollis* Sharp, 1882a: 4, pl. 1, fig. 2.

*Material examined:* **Cambyretá RS:** 27°49’26.86”S 56°50’51.46”W, 136 spec, 16.xi.2018, light trap, coll. J. Urcola; 27°54’00”S 56°53’06”W, 1 ♀, 17.xi.2018, coll. J. Urcola. **Laguna Iberá RS:** 28°32’49”S 57°11’48”W, 1 ♂ and 5 ♀♀, 14.ii.2018, light trap, coll. J. Urcola; 28°33’54”S 57°12’59”W, 1 ♂ and 7 ♀♀, 15.ii.2018, coll. J. Urcola; 28°33’21”S 57°11’40”W, 2 ♀♀, 16.ii.2018, coll. J. Urcola; 28°33’21”S 57°11’40”W, 1 ♀, 7.xi.2019, coll. J. Urcola; 28°32’46”S 57°11’45”W, 10 ♂♂ and 17 ♀♀, 7.xi.2019, light trap, coll. J. Urcola; 28°32’46”S 57°11’45”W, 78 spec, 8.xi.2019, light trap, coll. J. Urcola; 28°32’46”S 57°11’45”W, 100 spec, 14.xi.2019, light trap, coll. J. Urcola. **San Nicolás RS:** 28°07’43”S 57°26’09”W, 2 ♀♀, 6.xi.2022, coll. J. Urcola; 28°07’43”S 57°26’09”W, 1 ♀, 10.xi.2022, coll. J. Urcola. **Yahaveré RS:** 28°23’16”S 57°52’36”W, 1 ♂, 7.xi.2015, coll. S. Mazzucconi.

*Distribution:* Belize (Scheers and Thomaes 2017), Bolivia (Grosso 1979), Brazil (Zimmermann 1921; Benetti et al. 2003; Benetti and Régil Cueto 2004), Costa Rica (Blanco Aller 2015), Cuba (Megna and Deler 2006), Guatemala (Sharp 1882b), Mexico (Régimbart 1895; Arce-Pérez et al. 2018), Panama (Sharp 1882b), Paraguay (Grosso 1979), Argentina (provinces): Chaco (Grosso 1979; Libonatti et al. 2013), Corrientes (Grosso 1979; Torres et al. 2012), Formosa (Grosso 1979), Misiones (Grosso 1979), Santa Fe (Grosso 1979; Macchia et al. 2015).

*Habitat:* This species has been found in ponds and slow-flowing streams exposed to high insolation, with muddy bottom and abundant aquatic vegetation (Benetti and Régil Cueto 2004; Torres et al. 2012; Libonatti et al. 2013; this study). Specimens were also collected using light traps (Torres et al. 2012; Libonatti et al. 2013; Macchia et al. 2015; this study).

Genus *Prionohydrus* Gómez and Miller, 2013

*Remarks:* This genus of small neotropical beetles currently includes five valid species (Gómez and Miller 2013; García 2018; Urcola et al. 2024b). In Argentina, only the species listed below has been recorded to date.

*Prionohydrus cambyreta* Urcola, Baca, Rodriguez and Michat, 2024

Figs 11O, P

*Prionohydrus cambyreta* Urcola, Baca, Rodriguez and Michat, 2024: 484‒491, figs 1‒7.

*Material examined:* **Cambyretá RS:** 27°49’00”S 56°50’00”W, 16 ♂♂ and 79 ♀♀, 16.xi.2018, light trap, coll. J. Urcola.

*Distribution:* Argentina: to date, only known from Corrientes Province (Urcola et al. 2024b).

*Habitat:* Unknown. This species was collected using light trap (Urcola et al. 2024b).

Genus *Suphis* Aubé, 1836

*Remarks:* The genus *Suphis* includes distinctive members of Noteridae characterized by their robust and dorsally convex body shape. Currently, 11 species are known (Nilsson 2011), six of which occur in Argentina (Grosso 1993): *Suphis cimicoides* Aubé, 1937, *Suphis fluviatilis* Guignot, 1948, *Suphis freudei* Mouchamps, 1955, *Suphis minutus* Régimbart, 1903, *Suphis notaticollis* Zimmermann, 1921, and *Suphis werneri* Guignot, 1940.

*Suphis cimicoides* Aubé, 1837

Figs 12A, B

*Suphis cimicoides* Aubé, 1837 (in Aubé 1836–1838): 209, 210, pl. 24, fig. 5.

*Material examined:* **Cambyretá RS:** 27°51’27”S 56°54’10”W, 2 ♀♀, 16.xi.2018, coll. J. Urcola; 27°49’27”S 56°50’51”W, 1 ♂ and 5 ♀♀, 16.xi.2018, light trap, coll. J. Urcola; 27°54’10”S 56°53’14”W, 1 ♂ and 2 ♀♀, 17.xi.2018, coll. J. Urcola; 27°54’00”S 56°53’06”W, 1 ♀, 17.xi.2018, coll. J. Urcola; 27°52’26”S 56°52’49”W, 5 ♂♂ and 5 ♀♀, 19.xi.2018, coll. J. Urcola; **Laguna Iberá RS:** 28°33’29”S 57°11’38”W, 1 ♂, 2.xii.2012, coll. S. Mazzucconi; 28°33’54”S 57°12’59”W, 3 ♂♂ and 12 ♀♀, 15.ii.2018, coll. J. Urcola; 28°32’48”S 57°11’48”W, 100 spec, 17.ii.2018, coll. J. Urcola; 28°32’46”S 57°12’05”W, 6 ♂♂ and 3 ♀♀, 6.xi.2019, coll. J. Urcola; 6 ♂♂ 28°32’46” S 57°11’05” W, 1 ♀, 6.xi.2019, coll. J. Urcola; 28°32’46”S 57°11’45”W, 1 ♂ and 4 ♀♀, 7.xi.2019, light trap, coll. J. Urcola; 28°33’21” S 57°11’40” W, 4 ♂♂ and 8 ♀♀, 7.xi.2019, coll. J. Urcola; 28°32’46”S 57°11’45”W, , 1 ♂ and 6 ♀♀, 8.xi.2019, light trap, coll. J. Urcola; 28°33’01” S 57°12’45”W, 1 ♂ and 5 ♀♀, 11.xi.2019, coll. J. Urcola; 28°32’46”S 57°11’45”W, 2 ♀♀, 12.xi.2019, light trap, coll. J. Urcola; 28°32’46”S 57°11’45”W, 142 spec, 14.xi.2019, light trap, coll. J. Urcola; **San Nicolás RS:** [28°07’41”S 57°26’04”W], 1 ♂ and 1 ♀, 13–15.xii.2013, light trap, coll. S. Mazzucconi; 28°07’51”S 57°26’02”W, 2 ♀, 4.xi.2022, coll. J. Urcola and M. Urcola; 28°07’43”S 57°26’09”W; 1 ♀, 6.xi.2022, coll. J. Urcola; 28°07’43”S 57°26’09”W, 2 ♂♂ and 4 ♀, 6.xi.2022, coll. J. Urcola; 28°10’48”S 57°26’46”W, 1 ♂, 7.xi.2022, coll. J. Urcola; 28°07’39”S 57°25’59”W, 1 ♀, 8.xi.2022, coll. J. Urcola; 28°07’43” S 57°26’23”W; 1 ♂, 8.xi.2022, coll. J. Urcola; 28°07’43”S 57°26’09”W, 1 ♀, 10.xi.2022, coll. J. Urcola. **Yahaveré RS:** 28°23’16”S 57°52’36”W, 1 ♀, 7.xi.2015, coll. S. Mazzucconi.

*Distribution:* Bolivia (Grosso 1979, 1993), Brazil (Aubé 1837 (in Aubé 1836–1838); Zimmermann 1921), French Guiana (Aubé 1837 (in Aubé 1836–1838); Régimbart 1904; Mouchamps 1955), Guadeloupe (Régimbart 1903), Mexico (Arce-Pérez 2004), Paraguay (Mouchamps 1955; Grosso 1979, 1993), Uruguay (Grosso 1979, 1993), Argentina (provinces): Buenos Aires (Régimbart 1903; Bruch 1915, 1927; Grosso 1979, 1993; Fontanarrosa et al. 2009; Urcola et al. 2019b), Chaco (Régimbart 1903; Grosso 1979, 1993; Libonatti et al. 2013), Corrientes (Régimbart 1903; Grosso 1979, 1993; Torres et al. 2012), Entre Ríos (Grosso 1979, 1993), Formosa (Grosso 1979, 1993), Misiones (Bruch 1915), Salta (Régimbart 1903; Grosso 1979, 1993), Santa Fe (Grosso 1979, 1993; Macchia et al. 2015).

*Habitat:* This species, with a broad geographical distribution, has been found in a wide variety of habitats, including lagoons, ponds, urban rain pools, rivers, and streams (Fontanarrosa et al. 2009; Torres et al. 2012; Libonatti et al. 2013; Urcola et al. 2019b; this study). It was also collected using light traps (Torres et al. 2012; Libonatti et al. 2013; Macchia et al. 2015; this study).

*Suphis freudei* Mouchamps, 1955

Figs 12C, D

*Suphis freudei* Mouchamps, 1955: 4, 5.

*Material examined:* **San Nicolás RS:** [28°07’41”S 57°26’04”W], 2 ♂♂ and 2 ♀♀, 13–15.xii.2013, light trap, coll. S. Mazzucconi.

*Distribution*: Paraguay (Mouchamps 1955; Grosso 1979, 1993), Argentina (provinces): Buenos Aires (Grosso 1979, 1993), Chaco (Grosso 1979, 1993), Corrientes (Torres et al. 2012).

*Habitat:* Torres et al. (2012) found this species in a shallow pond characterized by high exposure to sunlight, muddy substrate, and abundant aquatic vegetation. Specimens were also collected using light traps (Torres et al. 2012; this study).

*Suphis notaticollis* Zimmermann, 1921

Figs 12E, F

*Suphis notaticollis* Zimmermann, 1921: 184, 185.

*Material examined:* **Laguna Iberá RS:** 28°32’46”S 57°11’45”W, 1 ♂, 7.xi.2019, light trap, coll. J. Urcola. **San Nicolás RS:** 28°10’48”S 57°26’46”W, 7 ♂♂ and 17 ♀♀, coll. J. Urcola.

*Distribution:* Brazil (Benetti et al. 2003; Benetti and Régil Cueto 2004), Paraguay (Guignot 1950; Grosso 1979, 1993), Argentina (provinces): Buenos Aires (Zimmermann 1921; Grosso 1979, 1993; Fontanarrosa et al. 2009), Chaco (Grosso 1979, 1993; Libonatti et al. 2013), Corrientes (Grosso 1979, 1993), Entre Ríos (Grosso 1979, 1993), Formosa (Grosso 1993), Salta (Grosso 1993), Santa Fe (Grosso 1979, 1993; Macchia et al. 2015), Santiago del Estero (Grosso 1979, 1993), Tucumán (Grosso 1993).

*Habitat:* This species has been found in both temporary (Fontanarrosa et al. 2009; Libonatti et al. 2013) and permanent ponds (this study), as well as in streams with very slow currents (Libonatti et al. 2013). All these habitats are characterized by high exposure to sunlight, muddy bottom, and near-complete vegetation cover. Specimens were also collected using light traps (Libonatti et al. 2013; Macchia et al. 2015; this study).

*Remarks:* The records of *S. fluviatilis* for the provinces of Chaco (Libonatti et al. 2013) and Santa Fe (Macchia et al. 2015), correspond to *S. notaticollis* (J. Urcola, pers. obs.).

Genus *Suphisellus* Crotch, 1873

*Remarks: Suphisellus* is the largest noterid genus in the Americas. It is endemic to this continent and includes more than 58 species distributed from Canada to central Argentina (Urcola and Michat 2023a). So far, 26 species have been recorded in this last (Urcola and Michat 2023b). In our study, 14 taxa were identified at the species level, which are listed below, as well as four taxa that could not be reliably determined to species. One of them shows a morphological match with the specimens reported by Torres et al. (2012) as *Suphisellus hieroglyphicus* Zimmermann, 1921; however, after a detailed analysis of certain characters, it may represent a different species.

*Suphisellus balzani* (Régimbart, 1889)

Figs 13A, B

*Canthydrus balzani* Régimbart, 1889a: 259.

*Material examined:* **Cambyretá RS:** 27°49’27”S 56°50’51”W, 8 ♂♂ and 18 ♀♀, 16.xi.2018, light trap, coll. J. Urcola. **Laguna Iberá RS:** 28°32’49”S 57°11’48”W, 2 ♀♀, 14.ii.2018, light trap, coll. J. Urcola; 28°32’56”S 57°11’58”W, 1 ♀, 14.xi.2019, coll. J. Urcola; 28°32’46”S 57°11’45”W, 1 ♂, light trap, coll. J. Urcola.

*Distribution:* Brazil (Régimbart 1889a, 1895; Zimmermann 1921), Paraguay (Régimbart 1889a; Grosso 1979), Argentina (provinces): Buenos Aires (Bruch 1915; Grosso 1979), Chaco (Régimbart 1889a), Corrientes (Grosso 1979; Torres et al. 2012), Entre Ríos (Régimbart 1903), Misiones (Grosso 1979), Santa Fe (Bruch 1915; Grosso 1979), Tucumán (Grosso 1979).

*Habitat:* Torres et al. (2012) found this species in a slow-flowing stream with high sunlight exposure, a muddy bottom, and abundant vegetation. Specimens were also collected using light traps (Torres et al. 2012; this study).

*Suphisellus flavopictus* (Régimbart, 1889)

Figs 13C, D

*Canthydrus flavopictus* Régimbart, 1889a: 260, 261.

*Material examined:* **Cambyretá RS:** 27°49’27”S 56°53’06”W, 29 spec, 16.xi.2018, light trap, coll. J. Urcola; 27°54’00”S 56°53’06”W, 1 ♂, 17.xi.2018, coll. J. Urcola. **Laguna Iberá RS:** 28°33’54”S 57°12’59”W, 9 ♂♂ and 15 ♀♀, 15.ii.2018, coll. J. Urcola; 28°33’21”S 57°11’40”W, 1 ♂ and 1 ♀, 16.ii.2018, coll. J. Urcola; 28°32’48”S 57°11’48”W, 1 ♂ and 1 ♀, 17.ii.2018, coll. J. Urcola; 28°32’48”S 57°11’48”W, 1 ♀, 5.xi.2019, coll. J. Urcola; 28°33’21”S 57°11’40”W, 1 ♀, 7.xi.2019, coll. J. Urcola; 28°32’46”S 57°11’45”W, 1 ♂ and 3 ♀♀, 7.xi.2019, light trap, coll. J. Urcola; 28°32’46”S 57°11’45”W, 6 ♂♂ and 3 ♀♀, 8.xi.2019, light trap, coll. J. Urcola; 28°33’54” S 57°12’59”W, 1 ♂ and 1 ♀, 10.xi.2019, coll. J. Urcola; 28°33’08”S 57°12’48”W, 1 ♂, 12.xi.2019, coll. J. Urcola; 28°32’56”S 57°11’58”W, 1 ♂ 17.xi.2019, coll. J. Urcola; 28°32’46”S 57°11’45”W, 11 ♂♂ and 18 ♀♀, 14.xi.2019, light trap, coll. J. Urcola.

*Distribution:* Bolivia (Zimmermann 1921; Grosso 1979), Paraguay (Régimbart 1889a; Grosso 1979), Venezuela (García 2020), Argentina (provinces): Buenos Aires (Grosso 1979; von Ellenrieder and Fernández 2000), Chaco (Régimbart 1889a; Bruch 1915; Grosso 1979; Libonatti et al. 2013), Corrientes (Grosso 1979; Torres et al. 2012; Gómez Lutz et al. 2012, 2015), Entre Ríos (Grosso 1979), Formosa (Grosso 1979), Salta (Grosso 1979), Santa Fe (Bruch 1915; Grosso 1979; Macchia et al. 2015).

*Habitat:* This species has been recorded in a wide range of lentic environments, including temporary and semi-permanent ponds (von Ellenrieder and Fernández 2000; Libonatti et al. 2013; this study), small lagoons (Gómez Lutz et al. 2012; Torres et al. 2012), and rice fields (Gómez Lutz et al. 2015), and also in lotic environments, including artificial channels (this study), streams (Torres et al. 2012; Libonatti et al. 2013), and rivers (Libonatti et al. 2013; García 2020). All these habitats are characterized by high exposure to sunlight, muddy substrates, and abundant aquatic vegetation. Specimens were also collected using light traps (Torres et al. 2012; Libonatti et al. 2013; Macchia et al. 2015; this study).

*Suphisellus grammicus* (Sharp, 1882)

Figs 13E, F

*Canthydrus grammicus* Sharp, 1882a: 274.

*Material examined:* **Cambyretá RS:** 27°49’27”S 56°50’51”W, 1 ♂ and 2 ♀♀, 16.xi.2018, light trap, coll. J. Urcola. **Laguna Iberá RS:** 28°32’49”S 57°11’48”W, 3 ♂♂ and 5 ♀♀, 14.ii.2018, light trap, coll. J. Urcola; 28°32’49”S 57°11’48”W, 1 ♀, 16.ii.2018, light trap, coll. J. Urcola; 28°32’46”S 57°11’45”W, 2 ♂♂ and 5 ♀♀, 7.xi.2019, light trap, coll. J. Urcola; 28°32’46”S 57°11’45”W, 2 ♀♀, 8.xi.2019, light trap, coll. J. Urcola; 28°33’01”S 57°12’45”W, 1 ♀, 11.xi.2019, coll. J. Urcola; 28°32’46”S 57°11’45”W, 1 ♀, 12.xi.2019, light trap, coll. J. Urcola; 28°32’46”S 57°11’45”W, 88 spec, 14.xi.2019, light trap, coll. J. Urcola. **San Nicolás RS:** 28°10’48”S 57°26’46”W, 2 ♀♀, 7.xi.2022, coll. J. Urcola.

*Distribution:* Bolivia (Régimbart 1899a; Grosso 1979), Brazil (Sharp 1882a; Régimbart 1903; Zimmermann 1921; Grosso 1979), Paraguay (Zimmermann 1919; Grosso 1979; Urcola et al. 2020), Peru (Chaboo and Shepard 2015), Argentina (provinces): Buenos Aires (Régimbart 1903; Bruch 1915; Grosso 1979), Chaco (Régimbart 1889a; Grosso 1979; Libonatti et al. 2013; Urcola et al. 2020), Córdoba (Grosso 1979), Corrientes (Gómez Lutz et al. 2012; Torres et al. 2012; Urcola et al. 2020), Entre Ríos (Régimbart 1903; Urcola et al. 2020), Formosa (Grosso 1979; Urcola et al. 2020), Jujuy (Grosso 1979), Misiones (Bruch 1915), Salta (Grosso 1979), Santa Fe (Grosso 1979; Macchia et al. 2015; Urcola et al. 2020), Tucumán (Bruch 1915; Grosso 1979).

*Habitat:* This species has been found in temporary and semi-permanent ponds (this study), lagoons (Gómez Lutz et al. 2012; Torres et al. 2012), and small streams with very slow current (Torres et al. 2012). All these habitats were characterized by high exposure to sunlight and abundant aquatic vegetation. Additionally, specimens were collected using light traps (Torres et al. 2012; Libonatti et al. 2013; Macchia et al. 2015; this study).

*Suphisellus grossus* (Sharp, 1882)

Figs 13G, H

*Canthydrus grossus* Sharp, 1882a: 270.

*Material examined:* **Laguna Iberá RS:** 28°32’49”S 57°11’48”W, 1 ♂, 14.ii.2018, light trap, coll. J. Urcola; 28°32’46”S 57°11’45”W, 1 ♂ and 1 ♀, 10.xi.2019, light trap, coll. J. Urcola; 28°32’46”S 57°11’45”W, 51 spec, 14.xi.2019, light trap, coll. J. Urcola.

*Distribution:* Brazil (Sharp 1882a; Zimmermann 1921), Paraguay (Grosso 1979), Argentina (provinces): Chaco (Régimbart 1889a; Grosso 1979; Libonatti et al. 2013), Corrientes (Torres et al. 2012), Formosa (Grosso 1979).

*Habitat:* Torres et al. (2012) recorded this species in a small stream with very slow current, high insolation, muddy substrate, and abundant aquatic vegetation. It was also collected using light traps (Torres et al. 2012; Libonatti et al. 2013; this study).

*Suphisellus* cf. *nigrinus* (Aubé, 1838)

Figs 13I, J

*Hydrocanthus nigrinus* Aubé, 1838 (in Aubé 1836–1838): 411.

*Material examined:* **Cambyretá RS:** 27°51’27”S 56°54’10”W, 1 ♂ and 3 ♀♀, 16.xi.2018, coll. J. Urcola; 27°49’27”S 56°50’51”W, 254 spec, 16.xi.2018, light trap, coll. J. Urcola; 27°54’10”S 56°53’14”W, 27 spec, 17.xi.2018, coll. J. Urcola; 27°54’00”S 56°53’06”W, 3 ♂ and 2 ♀♀, 17.xi.2018, coll. J. Urcola; 27°49’27”S 56°50’51”W, 1 ♂ and 1 ♀, 17.xi.2018, light trap, coll. J. Urcola; 27°52’11”S 56°52’59”W, 2 ♀♀, 18.xi.2018, coll. J. Urcola; 27°52’11”S 56°52’59”W, 1 ♀, 18.xi.2018, coll. J. Urcola; 27°52’15”S 56°53’00”W, 2 ♀♀, 18.xi.2018, coll. J. Urcola; 27°52’26”S 56°52’49”W, 1 spec, 19.xi.2018, coll. J. Urcola. **Galarza RS:** [28°04’40”S 56°44’29”W], 2 ♂♂, 17.iv.2013, coll. S. Mazzucconi. **Laguna Iberá RS:** 28°32’47”S 57°11’44”W, 2 ♂♂ and 6 ♀♀, 14.xii.2018, coll. J. Urcola; 28°32’49”S 57°11’48”W, 31 spec, 14.ii.2018, light trap, coll. J. Urcola; 28°33’54”S 57°12’59”W, 56 spec, 15.ii.2018, coll. J. Urcola; 28°32’49”S 57°11’48”W, 4 ♂♂ and 6 ♀♀, 16.ii.2018, light trap, coll. J. Urcola; 28°32’48”S 57°11’48”W, 33 spec, 17.ii.2018, coll. J. Urcola; 28°32’48”S 57°11’48”W, 1 ♂ and 3 ♀♀, 5.xi.2019, coll. J. Urcola; 28°32’51”S 57°11’53”W, 4 ♂♂ and 11 ♀♀, 6.xi.2019, coll. J. Urcola; 28°32’46”S 57°12’05”W, 4 spec, 6.xi.2019, coll. J. Urcola; 28°33’21”S 57°11’40”W, 2 ♂♂ and 1 ♀, 7.xi.2019, coll. J. Urcola; 28°32’46”S 57°11’45”W, 64 spec, 7.xi.2019, light trap, coll. J. Urcola; 28°32’51”S 57°11’53”W, 5 ♂♂ and 18 ♀♀, 9.xi.2019, coll. J. Urcola; 28°33’54”S 57°12’59”W, 3 ♀♀, 10.xi.2019, coll. J. Urcola; 28°33’08”S 57°12’48”W, 2 ♂♂, 11.xi.2019, coll. J. Urcola; 28°33’17”S 57°12’54”W, 2 ♂♂, 12.xi.2019, coll. J. Urcola; 28°33’08”S 57°12’48”W, 1 ♂ and 6 ♀♀, 12.xi.2019, coll. J. Urcola; 28°32’46”S 57°11’45”W, 3 ♂♂ and 6 ♀♀, 12.xi.2019, light trap, coll. J. Urcola; 28°32’46”S 57°11’45”W, 303 spec, 14.xi.2019, light trap, coll. J. Urcola. **San Nicolás RS:** 28°07’51”S 57°26’02”W, 1 ♂ and 2 ♀♀, 4.xi.2022, coll. J. Urcola; 28°08’17”S 57°25’49”W, 1 ♀, 5.xi.2022, coll. J. Urcola; 28°07’43”S 57°26’09”W, 2 ♂♂, 5.xi.2022, coll. J. Urcola; 28°07’43”S 57°26’09”W, 1 ♂, 6.xi.2022, coll. J. Urcola; 28°10’48”S 57°26’46”W, 14 ♂♂ and 10 ♀♀, 7.xi.2022, coll. J. Urcola; 28°09’25”S 57°26’34”W, 3 ♀♀, 8.xi.2022, coll. J. Urcola; 28°07’39”S 57°25’59”W, 1 ♂ and 3 ♀♀, 8.xi.2022, coll. J. Urcola; 28°07’20”S 57°25’57”W, 2 ♂♂ and 3 ♀♀, 9.xi.2022, coll. J. Urcola; 28°07’43”S 57°26’23”W, 1 ♂, 9.xi.2022, coll. J. Urcola; 28°07’43”S 57°26’09”W, 1 ♀, 9.xi.2022, coll. J. Urcola; 28°07’42”S 57°26’02”W, 1 ♂ and 2 ♀♀, 9.xi.2022, light trap, coll. J. Urcola; 28°07’43”S 57°26’09”W, 1 ♂ and 3 ♀♀, 10.xi.2022, coll. J. Urcola. **Yahaveré RS:** [28°32’30”S 57°44’45”W], 1 ♂, 7.xi.2015, coll. S. Mazzucconi.

*Distribution:* Antilles (Aubé 1838 (in Aubé 1836–1838)), Belize (Scheers and Thomaes 2017), Bolivia (Grosso 1979), Brazil (Aubé 1838 (in Aubé 1836–1838); Sharp 1882a; Zimmermann 1921; Grosso 1979; Benetti et al. 2003; Benetti and Hamada 2003; Benetti and Régil Cueto 2004), Costa Rica (Blanco Aller and Régil 2013; Blanco Aller 2015), Cuba (Megna and Deler 2006; Leyva Escobar et al. 2014), Mexico (Arce-Pérez et al. 2018), Paraguay (Régimbart 1889a; Grosso 1979), Argentina (provinces): Buenos Aires (Grosso 1979; von Ellenrieder and Fernández 2000; Fernández and López Ruf 2006; Fernández et al. 2010), Chaco (Régimbart 1889a; Grosso 1979; Libonatti et al. 2013), Corrientes (Grosso 1979; Torres et al. 2012; Gómez Lutz et al. 2015), Entre Ríos (Grosso 1979), Formosa (Grosso 1979), La Rioja (Grosso 1979), Misiones (Grosso 1979), Salta (Grosso 1979), Santa Fe (Grosso 1979; Macchia et al. 2015), Tucumán (Grosso 1979).

*Habitat:* This widely distributed species, ranging from Mexico to Argentina, has been found in a variety of habitats, including temporary and permanent ponds (von Ellenrieder and Fernández 2000; Fernández and López Ruf 2006; Torres et al. 2012; this study), small marshes (Torres et al. 2012), small lagoons (Benetti and Hamada 2003; Torres et al. 2012; Leyva Escobar et al. 2015; this study), slow-flowing streams (Benetti and Hamada 2003; Fernández and López Ruf 2006; Fernández et al. 2010; Torres et al. 2012), artificial channels (this study), and rice fields (Gómez Lutz et al. 2015). Most of these environments were characterized by muddy bottom and abundant aquatic vegetation. Specimens have also been collected using light traps (Torres et al. 2012; Libonatti et al. 2013; Macchia et al. 2015; this study).

*Remarks:* The male specimens studied here exhibit a left lateral lobe of the genitalia that is thin, slightly curved, and bears a tuft of dense setae at the apex.

*Suphisellus* cf. *pereirai* Guignot, 1958

Figs 13K, L

*Suphisellus pereirai* Guignot, 1958: 37.

*Material examined:* **Cambyretá RS:** 27°49’27”S 56°50’51”W, 1 ♂ and 2 ♀♀, 16.xi.2018, light trap, coll. J. Urcola; 27°54’00”S 56°53’06”W, 1 ♂ and 1 ♀, 17.xi.2018, coll. J. Urcola; 27°52’15”S 56°53’00”W, 1 ♂ and 1 ♀, 18.xi.2018, coll. J. Urcola. **Laguna Iberá RS:** 28°33’54”S 57°12’59”W, 1 ♂ and 2 ♀♀, 15.ii.2018, coll. J. Urcola; 28°32’46”S 57°11’45”W, 1 ♂, 8.xi.2019, light trap, coll. J. Urcola; 28°32’46”S 57°11’45”W, 1 ♂ and 2 ♀♀, 14.xi.2019, light trap, coll. J. Urcola.

*Distribution:* Brazil (Guignot 1958), Argentina: Corrientes Province (**new record**).

*Habitat:* In this study, specimens of this species were found in semi-permanent ponds and artificial channels, characterized by high exposure to sunlight, muddy bottom, and abundant aquatic vegetation. Specimens were also collected using light traps.

*Suphisellus pinguiculus* (Régimbart, 1903)

Figs 13M, N

*Canthydrus pinguiculus* Régimbart, 1903: 62.

*Material examined:* **Cambyretá RS:** 27°49’27”S 56°50’51”W, 2 ♂♂ and 19 ♀♀, 16.xi.2018, light trap, coll. J. Urcola; 27°54’10”S 56°53’14”W, 1 ♀, 17.xi.2018, coll. J. Urcola. **Laguna Iberá RS:** 28°32’49”S 57°11’48”W, 1 ♀♀, 14.ii.2018, light trap, coll. J. Urcola; 28°32’46”S 57°11’45”W, 1 ♀, 7.xi.2019 light trap, coll. J. Urcola; 28°32’46”S 57°11’45”W, 2 ♂♂ and 5 ♀♀, 14.xi.2019 light trap, coll. J. Urcola. **San Nicolás RS:** 28°07’43”S 57°26’09”W, 2 ♀♀, 5.xi.2022, coll. J. Urcola; 28°07’43”S 57°26’09”W, 2 ♀♀, 6.xi.2022, coll. J. Urcola; 28°07’39”S 57°25’59”W, 2 ♂♂, 8.xi.2022, coll. J. Urcola; 28°07’43”S 57°26’09”W, 1 ♀, 10.xi.2022, coll. J. Urcola.

*Distribution: Distribution:* Bolivia (Grosso 1979), Brazil (Régimbart 1903; Zimmermann 1921; Benetti et al. 2003; Benetti and Régil Cueto 2004), Argentina (provinces): Buenos Aires (Régimbart 1903; Bruch 1915; Zimmermann 1919; Grosso 1979; Fernández and López Ruf 1999; von Ellenrieder and Fernández 2000), Corrientes (Grosso 1979; Torres et al. 2012), Salta (Grosso 1979), Santa Fe (Grosso 1979).

*Habitat:* This species has been found in temporary and semi-permanent ponds (von Ellenrieder and Fernández 2000; Benetti and Régil Cueto 2004; this study), as well as in streams with very slow current (Torres et al. 2012). All these environments are characterized by high exposure to sunlight, muddy bottom, and abundant aquatic vegetation. Specimens were also collected using light traps (Torres et al. 2012; this study).

*Suphisellus punctipennis* (Sharp, 1882)

Figs 13O, P

*Pronoterus punctipennis* Sharp, 1882a: 263.

*Material examined:* **Cambyretá RS:** 27°49’27”S 56°50’51”W, 271 spec, 16.xi.2018, light trap, coll. J. Urcola; 27°54’27”S 56°53’19”W, 1 ♂, 17.xi.2018, coll. J. Urcola; 27°54’10”S 56°53’14”W, 4 ♂♂, 17.xi.2018, coll. J. Urcola; 27°54’00”S 56°53’06”W, 2 ♂♂ and 3 ♀♀, 17.xi.2018, coll. J. Urcola; 27°52’11”S 56°52’59”W, 1 ♂, 18.xi.2018, coll. J. Urcola; 27°52’15”S 56°53’00”W, 1 ♂ and 1 ♀, 18.xi.2018, coll. J. Urcola. **Laguna Iberá RS:** 28°32’46”S 57°11’45”W, 1 ♀, 8.xi.2019, light trap, coll. J. Urcola; 28°33’01”S 57°12’45”W, 1 ♀, 11.xi.2019, coll. J. Urcola; 28°32’46”S 57°11’45”W, 2 ♂♂ and 5 ♀♀, 14.xi.2019, light trap, coll. J. Urcola. **San Nicolás RS:** 28°07’43”S 57°26’09”W, 1 ♀, 5.xi.2022, coll. J. Urcola; 28°07’39”S 57°25’59”W, 2 ♀♀, 8.xi.2022, coll. J. Urcola. **Yahaveré RS:** 28°23’16”S 57°54’26”W, 1 ♀, 5.xi.2015, coll. S. Mazzucconi.

*Distribution:* Bolivia (Grosso 1979), Brazil (Sharp 1882a; Régimbart 1895; Zimmermann 1921; Benetti et al. 2003; Benetti and Hamada 2003; Benetti and Régil Cueto 2004), Paraguay (Grosso 1979), Argentina (provinces): Buenos Aires (Bruch 1915; Grosso 1979; Fernández and López Ruf 2006), Chaco (Régimbart 1889a; Bruch 1915; Grosso 1979; Libonatti et al. 2013), Corrientes (Grosso 1979; Torres et al. 2012), Formosa (Grosso 1979), Salta (Grosso 1979), Santa Fe (Grosso 1979), Tucumán (Grosso 1979).

*Habitat:* This species has been found in temporary and semi-permanent ponds (Fernández and López Ruf 2006; Torres et al. 2012; present study), as well as in shallow, slow-flowing streams (Benetti and Hamada 2003). All these habitats had abundant aquatic vegetation. Specimens were also collected using light traps (Torres et al. 2012; Libonatti et al. 2013; present study).

*Remarks:* This species was originally included in the genus Pronoterus Sharp, 1882, until a recent study based on molecular data (Baca et al. 2017) synonymized Pronoterus with Suphisellus.

*Suphisellus remator* (Sharp, 1882)

Figs 13Q, R

*Canthydrus remator* Sharp, 1882a: 272.

*Material examined:* **Cambyretá RS:** 27°49’27”S 56°50’51”W, 1 ♂ and 1 ♀, 16.xi.2018, light trap, coll. J. Urcola. **Laguna Iberá RS:** [28°32’47”S 57°11’45”W], 3 ♂♂ and 5 ♀♀, 1.xii.2012, light trap, coll. S. Mazzucconi; [28°32’47”S 57°11’45”W], 2 ♂♂ and 2 ♀♀, 2.xii.2012, light trap, coll. S. Mazzucconi. **San Nicolás RS:** 28°07’51”S 57°26’02”W, 2 spec, 4.xi.2022, coll. J. Urcola; 28°08’17”S 57°25’49”W, 1 ♀, 5.xi.2022, coll. J. Urcola; 28°07’43”S 57°26’09”W, 2 ♀♀, 6.xi.2022, coll. J. Urcola; 28°10’48”S 57°26’46”W, 73 spec, 7.xi.2022, coll. J. Urcola; 28°09’25”S 57°26’34”W, 1 ♂, 8.xi.2022, coll. J. Urcola; 28°07’39”S 57°25’59”W, 2 ♂♂ and 1 ♀, 8.xi.2022, coll. J. Urcola; 28°07’20”S 57°25’57”W, 1 ♂, 9.xi.2022, coll. J. Urcola; 28°07’43”S 57°26’23”W, 1 ♀, 9.xi.2022, coll. J. Urcola; 28°07’43”S 57°26’09”W, 1 ♂ and 3 ♀, 9.xi.2022, coll. J. Urcola; 28°07’42”S 57°26’02”W, 3 spec, 9.xi.2022, light trap, coll. J. Urcola; 28°07’43”S 57°26’09”W, 2 ♂♂, 10.xi.2022, coll. J. Urcola.

*Distribution:* Bolivia (Grosso 1979), Brazil (Zimmermann 1921; Benetti et al. 2003; Benetti and Régil Cueto 2004), Paraguay (Grosso 1979), Uruguay (Sharp 1882a; Régimbart 1903; Grosso 1979; Benetti and Garrido 2004), Argentina (provinces): Buenos Aires (Régimbart 1903; Bruch 1915; Grosso 1979), Chaco (Régimbart 1889a; Régimbart 1903; Bruch 1915; Grosso 1979; Libonatti et al. 2013), Córdoba (Régimbart 1903; Bruch 1915; Grosso 1979), Corrientes (Grosso 1979; Torres et al. 2012), Entre Ríos (Régimbart 1903; Grosso 1979), Formosa (Grosso 1979), Río Negro (Grosso 1979), Salta (Grosso 1979), Santa Fe (Grosso 1979; Macchia et al. 2015), Tucumán (Grosso 1979).

*Habitat:* This species has been found in a wide range of habitats, including temporary and semi-permanent ponds (Benetti and Régil Cueto 2004; Libonatti et al. 2013; this study), small lagoons (this study), artificial channels (this study), streams (Torres et al. 2012; Libonatti et al. 2013), and very slow-flowing rivers (Libonatti et al. 2013). In all cases, these habitats were characterized by high exposure to sunlight, muddy or sandy bottom, and abundant aquatic vegetation. Specimens were also collected using light traps (Torres et al. 2012; Libonatti et al. 2013; Macchia et al. 2015; this study).

*Suphisellus rotundatus* (Sharp, 1882)

Figs 13S, T

*Canthydrus rotundatus* Sharp, 1882a: 270.

*Material examined:* **Cambyretá RS:** 27°49’27”S 56°50’51”W, 1 ♀, 16.xi.2018, light trap, coll. J. Urcola; 27°52’26”S 56°52’49”W, 2 ♂♂ and 2 ♀♀, 19.xi.2018, coll. J. Urcola. **Laguna Iberá RS:** [28°32’47”S 57°11’45”W], 1 ♀, xii.2013, light trap, coll. S. Mazzucconi; 28°33’21”S 57°11’40”W, 1 ♂ and 5 ♀♀, 16.ii.2018, coll. J. Urcola; 28°34’13”S 57°10’13”W, 1 ♀, 16.ii.2018, coll. J. Urcola; 28°32’48”S 57°11’48”W, 1 ♂ and 1 ♀, 17.ii.2018, coll. J. Urcola; 28°32’46”S 57°11’45”W, 11 ♀♀, 7.xi.2019 light trap, coll. J. Urcola; 28°32’46”S 57°11’45”W, 4 ♂♂ and 8 ♀♀, 8.xi.2019 light trap, coll. J. Urcola; 28°32’46”S 57°11’45”W, 1 ♂, 12.xi.2019 light trap, coll. J. Urcola; 28°32’46”S 57°11’45”W, 118 spec, 14.xi.2019 light trap, coll. J. Urcola. **San Nicolás RS:** 28°07’43”S 57°26’09”W, 1 ♀, 5.xi.2022, coll. J. Urcola.

*Distribution:* Bolivia (Grosso 1979), Brazil (Sharp 1882a; Régimbart 1903; Zimmermann 1921; Grosso 1979), Paraguay (Grosso 1979), Argentina (provinces): Buenos Aires (Grosso 1979), Chaco (Régimbart 1889a; Bruch 1915; Grosso 1979; Libonatti et al. 2013), Corrientes (Grosso 1979; Torres et al. 2012; Gómez Lutz et al. 2012), Entre Ríos (Grosso 1979), Formosa (Grosso 1979), Salta (Grosso 1979), Santa Fe (Bruch 1915; Grosso 1979; Macchia et al. 2015).

*Habitat:* This species has been found in temporary and semi-permanent ponds (Libonatti et al. 2013; this study), small lagoons (Gómez Lutz et al. 2012; Torres et al. 2012), and very slow-flowing rivers (this study). These habitats were characterized by high exposure to sunlight, muddy bottom, and nearly complete aquatic vegetation cover. Specimens were also collected using light traps (Torres et al. 2012; Libonatti et al. 2013; Macchia et al. 2015; this study).

*Suphisellus* cf. *rufipes* (Sharp, 1882)

Figs 13U, V

*Canthydrus rufipes* Sharp, 1882a: 273.

*Material examined:* **Itatí RS:** [28°44’27”S 58°07’46”W], 1 ♀, 26.ix.2003 coll. S. Mazzucconi. **Laguna Iberá RS:** [28°32’47”S 57°11’45”W], 1 spec, 24.iii.2002 coll. S. Mazzucconi; **San Nicolás RS:** 28°07’43”S 57°26’09”W, 1 ♀, 10.xi.2022, coll. J. Urcola.

*Distribution:* Brazil (Sharp 1882a; Benetti et al. 2003; Benetti and Hamada 2003; Benetti and Régil Cueto 2004), Cuba (Sharp 1882a), Paraguay (Grosso 1979), Uruguay (Benetti and Garrido 2004), Argentina (provinces): Buenos Aires (Viana 1937; Grosso 1979; Macchia 2022; Macchia and Cicchino 2023), Catamarca (Grosso 1979), Corrientes (**new record**), Jujuy (Urcola et al. 2019c), Salta (Grosso 1979), Tucumán (Grosso 1979).

*Habitat:* Urcola et al. (2019c) collected larval and adult specimens of this species along the Yala-Termas de Reyes road (Jujuy Province), at an elevation of 1873 m a.s.l., in a temporary pond with abundant vegetation. In the hills of the Tandilia system (Buenos Aires Province), this species was recorded in temporary ponds with abundant vegetation cover and also in shaded areas, with the highest frequency of observation occurring during autumn and late spring (Macchia and Cicchino 2023). Additionally, it has been found in temporary and semi-permanent ponds exposed to high solar radiation, with muddy bottom and rich aquatic vegetation (this study), as well as in very slow-flowing streams (Benetti and Hamada 2003).

*Remarks:* In Argentina, only two species of *Suphisellus* with entirely black elytra have been recorded: *S. nigrinus* and *S. rufipes*. Young (1979) synonymized *S. rufipes* with *S. nigrinus*. However, examination of the male genitalia of the specimens studied here show a perfect match with the illustration of *S. rufipes* provided by Grosso (1979), and are distinct from those observed in the specimens assigned to *S. nigrinus* in this study. This clearly indicates the existence of two distinct species, diagnosable by differences in the male genitalia. To avoid generating further taxonomic confusion, we opted to use the name *S. rufipes*, pending examination of the type material of both species.

*Suphisellus rufulus* Zimmermann, 1921

Figs 13W, X

*Suphisellus rufulus* Zimmermann, 1921: 188.

*Material examined:* **Cambyretá RS:** 27°52’15”S 56°53’00”W, 4 ♂♂ and 3 ♀♀, 18.xi.2018, coll. J. Urcola. **Laguna Iberá RS:** 28°32’48”S 57°11’48”W, 1 ♀, 17.i.2018, coll. J. Urcola; 28°32’48”S 57°11’48”W, 2 ♀♀, 5.xi.2019, coll. J. Urcola; 28°33’21”S 57°11’40”W, 1 ♀, 7.xi.2019, coll. J. Urcola. **San Nicolás RS:** 28°07’43”S 57°26’09”W, 2 ♀♀, 6.xi.2022, coll. J. Urcola.

*Distribution:* Brazil (Zimmermann 1921), Paraguay (Grosso 1979), Argentina: Corrientes Province (Torres et al. 2012).

*Habitat:* This species has been found in permanent and semi-permanent ponds (this study), as well as in a small, slow-flowing stream (Torres et al. 2012). These environments are characterized by high exposure to sunlight, muddy bottom, and abundant aquatic vegetation.

*Suphisellus sexnotatus* (Régimbart, 1889)

Figs 13Y, Z

*Canthydrus sexnotatus* Régimbart, 1889b: 259, 260.

*Material examined:* **Cambyretá RS:** 27°54’27”S 56°53’19”W, 5 ♂♂ and 1 ♀, 17.xi.2018, coll. J. Urcola; 27°54’10”S 56°53’14”W, 2 ♂♂ and 2 ♀♀, 17.xi.2018, coll. J. Urcola; 27°52’11”S 56°52’59”W, 1 ♂ and 1 ♀, 18.xi.2018, coll. J. Urcola. **Laguna Iberá RS:** 28°32’49”S 57°11’48”W, 1 ♀, 14.ii.2018, light trap, coll. J. Urcola; 28°32’48”S 57°11’48”W, 2 ♀♀, 17.ii.2018, coll. J. Urcola; 28°32’46”S 57°11’45”W, 5 ♂♂ and 12 ♀♀, 14.xi.2019, light trap, coll. J. Urcola. **San Nicolás RS:** 28°07’43”S 57°26’09”W, 1 ♂ and 1 ♀, 6.xi.2022, coll. J. Urcola.

*Distribution:* Brazil (Régimbart 1889b), Paraguay (Grosso 1979), Argentina: Corrientes Province (Torres et al. 2012).

*Habitat:* In this study, specimens of this species were found in permanent and semi-permanent ponds characterized by high exposure to sunlight, muddy bottom, and abundant aquatic vegetation. Specimens were also collected using light traps (Torres et al. 2012; this study).

*Suphisellus variicollis* Zimmermann, 1921

Figs 13AA, AB

*Suphisellus variicollis* Zimmermann, 1921: 187.

*Material examined:* **Cambyretá RS:** 27°49’27”S 56°50’51”W, 6 ♂♂ and 10 ♀♀, 16.xi.2018, light trap, coll. J. Urcola. **Laguna Iberá RS:** 28°33’54”S 57°12’59”W, 1 ♂, 15,ii.2018, coll. J. Urcola; 28°32’46”S 57°11’45”W, 4 ♀♀, 14.xi.2019. **San Nicolás RS:** 28°07’51”S 57°26’02”W, 1 ♂, 4.xi.2022, coll. J. Urcola.

*Distribution:* Brazil (Zimmermann 1921), Paraguay (Grosso 1979), Argentina (provinces): Buenos Aires (Macchia 2022; Macchia and Cicchino 2023), Corrientes (Torres et al. 2012), Santa Fe (Zimmermann 1921).

*Habitat:* This species has been found in temporary ponds (Macchia and Cicchino 2023), semi-permanent ponds (this study), and irrigation channels (this study), all of which were characterized by abundant vegetation cover. Specimens were also collected using light traps (Torres et al. 2012; this study).

Subfamily Notomicrinae Zimmermann, 1919

Genus *Notomicrus* Sharp, 1882

*Remarks:* This genus of tiny beetles, distributed across the Neotropical, Indomalayan, and Oceanian regions, currently includes 17 valid species (Baca and Short 2021). In Argentina, three species have been recorded (Nilsson 2011): *Notomicrus brevicornis* Sharp, 1882; *Notomicrus reticulatus* Zimmermann, 1921; and *Notomicrus traili* Sharp, 1882. Torres et al. (2012) reported the presence of *N. brevicornis* and *N. traili* in Mburucuyá National Park (Corrientes Province, Argentina), and the specimens identified in this study are morphologically consistent with these species. However, a recent phylogenetic analysis (Baca et al. 2024) revealed that this genus contains several species complexes with strong phylogenetic structuring, including distinct but morphologically cryptic lineages with overlapping distributions. Given that a comprehensive revision of the genus has yet to be undertaken, we have been unable to confidently identify the two species found in this study.

**References**

Alarie Y, Megna YS, Deler-Hernandez A (2009b) First West Indies records of *Thermonectus succinctus* (Aubé, 1838), with notes on other Cuban species (Coleoptera: Dytiscidae). Koleopterologische Rundschau 79: 5‒16.

Alarie Y, Michat MC, Nilsson AN, Archangelsky M, Hendrich L (2009a) Larval morphology of *Rhantus* Dejean, 1833 (Coleoptera: Dytiscidae: Colymbetinae): descriptions of 22 species and phylogenetic considerations. Zootaxa 2317:1‒102. DOI [10.11646/zootaxa.2317.1.1](https://doi.org/10.11646/zootaxa.2317.1.1)

Alarie Y, Michat MC, Watanabe K, Shaverdo H, Wang L-J, Watts CHS (2022) An outlook on larval morphology of Copelatinae diving beetles with phylogenetic considerations (Coleoptera: Adephaga, Dytiscidae). Zootaxa 5175(2): 151–205. <https://doi.org/10.11646/zootaxa.5175.2.1>

Arce-Pérez R (1995) Lista preliminar de coleópteros acuáticos del estado de Morelos, México. Acta Zoológica Mexicana (nueva serie) 65: 43–53.

Arce-Pérez R (2004) Primer registro del género *Suphis* Aubé, 1836 (Coleoptera: Noteridae) para México. Folia Entomológica Mexicana 43(3): 321–322.

Arce-Pérez R, Gómez-Anaya JA, Epler JH (2018) The familiy Noteridae Thomson (Coleoptera: Adephaga) in Miradores Lagoon, Veracruz, Mexico, with a description of its assemblage. The Coleopterists Bulletin 72(1):75–83. DOI.org/10.1649/0010-065X-72.1.75

Archangelsky M, Michat MC (2014) Coleoptera, Haliplidae. In: Roig-Juñent S, Claps LE, Morrone JJ (Eds) Biodiversidad de artrópodos argentinos, Vol. 3. Editorial INSUE/Universidad Nacional de Tucumán, San Miguel de Tucumán, 467–473.

Aubé C (1836–1838) Hydrocanthares. In: Dejean PF: Iconographie et histoire naturelle des coléoptères d’Europe. Vol. 5. Paris: Méquignon-Marvis, xi + 416 pp + 46 pls. [Pp. 1–64 (1836), 65–224 (1837), 225–416 (1838).]

Aubé C (1838) Hydrocanthares et gyriniens. In: Dejean PF: Species géneral des coléoptères de la collection de M. le Comte Dejean. Vol. 6. Méquignon Père et Fils, Paris, xvi + 804 pp.

Babington CC (1842) Dytiscidae Darwinianae. The Transactions of the Entomological Society of London 3(1): 1–17 + 1 pl.

Baca SM, Gustafson GT, DeRaad DA, Alexander A, Hime PM, Short AEZ (2024) A shallow-scale phylogenomics approach reveals repeated patterns of diversification among sympatric lineages of cryptic Neotropical aquatic beetles (Coleoptera: Noteridae). Systematic Entomology 1‒20. <https://doi.org/10.1111/syen.12643>

Baca SM, Short AEZ (2021) Review of the New World *Notomicrus* Sharp (Coleoptera, Noteridae) I: Circumscription of species groups and review of the *josiahi* group with description of a new species from Brazil. ZooKeys 1025: 177‒201. https://doi.org/10.3897/zookeys.1025.60442

Baca SM, Toussaint EFA, Miller KB, Short AEZ (2017) Molecular phylogeny of the aquatic beetle family Noteridae (Coleoptera: Adephaga) with an emphasis on data partitioning strategies. Molecular Phylogenetics and Evolution 107: 282‒291. <https://doi.org/10.1016/j.ympev.2016.10.016>

Balfour-Browne J (1947) A revision of the genus *Bidessonotus* Régimbart (Coleoptera: Dytiscidae). Transactions of the Royal Entomological Society 98: 425‒448.

Balke M (1992) Taxonomische Untersuchungen an neotropischen Wasserkäfern der Gattung *Rhantus* Dejean (Insecta, Coleoptera: Dytiscidae). Reichenbachia 29(6): 27‒39.

Balke M (1993) Neotropische Wasserkäfer der Gattung *Rhantus* Dejean. IV. Liste und Notizen über die “großen” Arten (Coleoptera: Dytiscidae). Reichenbachia 30: 21‒32.

Balke M, Megna YS, Zenteno N, Figueroa L, Hendrich L (2020) New *Liodessus* species from the high Andes of Peru (Coleoptera: Dytiscidae, Bidessini). Zootaxa 4852(2): 151‒165. https://doi.org/10.11646/zootaxa.4852.2.1

Bánki O, Roskov Y, Döring M, Ower G, Hernández Robles DR, Plata Corredor CA, Stjernegaard Jeppesen T, Örn A, Vandepitte L, Pape T, Hobern D, Garnett S, Little H, DeWalt RE, Ma K, Miller J, Orrell T, Aalbu R, Abbott J, et al. (2024). Catalogue of Life (Version 2024-08-29). Catalogue of Life, Amsterdam, Netherlands. https://doi.org/10.48580/dgdwl

Benetti CJ, Garrido J (2004) Fauna de coleópteros acuáticos (Adephaga y Polyphaga) de Uruguay (América del Sur). Boletín de la Asociación Española de Entomología 28(1‒2): 153‒183.

Benetti CJ, Hamada N (2003) Fauna de coleópteros aquáticos (Insecta: Coleoptera) na Amazônia Central, Brasil. Acta Amazonica 33(4): 701‒710.

Benetti CJ, Régil Cueto JA (2004) Fauna composition of water beetles (Coleoptera: Adephaga) in seven water environments in the municipality of Gramado, RS, Brazil. Acta Limnologica Brasiliensia 16(1): 1‒11.

Benetti CJ, Régil Cueto JA, Garrido González J (2003) Estudio faunístico de Hydradephaga (Coleoptera: Dytiscidae, Gyrinidae, Haliplidae, Noteridae) en el Municipio de Gramado, sur de Brasil. Boletín de la Sociedad Entomológica Aragonesa 32(1): 37‒44.

Biström O (1990) Revision of the genus *Queda* Sharp (Coleoptera: Dytiscidae). Quaaestiones Entomologicae 26: 211‒220.

Blanco Aller R (2015) Catálogo y actualización corológica de los notéridos (Coleoptera: Adephaga: Noteridae) de Costa Rica. Boletín de la Sociedad Entomológica Aragonesa 56: 179‒186.

Blanco Aller R (2016) Contribución al conocimiento de los géneros *Megadytes* Sharp, 1882, *Thermonectus* Dejean, 1833 e *Hydaticus* Leach, 1817 (Coleoptera: Adephaga: Dytiscidae) en Costa Rica. Boletín de la Sociedad Entomológica Aragonesa 58: 197‒205.

Blanco Aller R, Régil JA (2013) Los adéfagos acuáticos (Coleoptera: Dytiscidae, Gyrinidae, Noteridae) de la Reserva Biológica Alberto Manuel Brenes (Alajuela, Costa Rica). Boletín de la Sociedad Entomológica Aragonesa 53: 293‒297.

Bousquet Y (2016) Litteratura Coleopterologica (1758–1900): a guide to selected books related to the taxonomy of Coleoptera with publication dates and notes. ZooKeys 583: 1‒776. DOI [10.3897/zookeys.583.7084](https://doi.org/10.3897/zookeys.583.7084)

Braga RB, Ferreira-Jr N (2016a) Vatellini Sharp (Coleoptera, Dytiscidae) from Brazil: two new species, new records, and a checklist. Zootaxa 4111(1): 77‒91. http://doi.org/10.11646/zootaxa.4111.1.7

Braga RB, Ferreira-Jr N (2016b) *Laccomimus xikrin* sp. nov. and new records of other species of *Laccomimus* Toledo & Michat, 2015 (Coleoptera: Dytiscidae; Laccophilinae) from Brazil. Zootaxa 4154(1): 96‒100. <http://doi.org/10.11646/zootaxa.4154.1.7>

Bruch C (1915) Catálogo sistemático de los coleópteros de la República Argentina. Revista del Museo de La Plata 19(2): 471‒479.

Bruch C (1927) Suplemento al catálogo sistemático de los coleópteros de la República Argentina. II. Addenda, corrigenda y lista de especies. Physis 8: 537‒553.

Brullé GA (1835‒1842) Insectes coléoptères: tribus carabiques, hydrocanthares, palpicornes. Pp. 1‒60 + pls. 1‒4. In: Blanchard E. & Brullé G.A. 1835-1847: Insectes de l’Amérique mériodionale recueillis par Alcide d’Orbigny. In: d’Orbigny A.D. 1835‒1847: Voyage dans l’Amérique méridionale (le Brézil, la République orientale de l'Uruguay, la République Argentine, la Patagonie, la République du Chili, la République de Bolivie, la République du Perou) exécuté pendant les années 1826, 1827, 1828, 1829, 1830, 1831, 1832 et 1833 par Alcide d’Orbigny. Tome sixième. 2.e partie: Insectes. Paris & Strasbourg: P. Bertrand & Ve Levrault, vi [unnumbered] + 222 pp + 32 pls. [pl. 1 published in 1835, pls. 2‒4 in 1836, pp. 1‒56 in 1837, pp. 57‒72 in 1842, see Bousquet (2016)]

Bustamante AN (2018) Registro de los coleópteros acuáticos en la región Cusco, Perú. The Biologist (Lima) 16(1): 35‒49.

Caputo FP, Nardi G, Bertolani P (2006) Observations of predaceous diving beetles (Insecta, Coleoptera, Dytiscidae) attacking Terecay, Podocnemis unifilis, (Reptilia, Testudines, Pelomedusidae) in Ecuador. Herpetological Bulletin 96: 14‒16.

Chaboo CS, Shepard WD (2015) Beetles (Coleoptera) of Peru: A survey of the families. Dytiscidae, Gyrinidae, Haliplidae, and Noteridae (Suborder Adephaga). Journal of the Kansas Entomological Society 88(2): 146–150.

Corigliano MC, Raffaini GB (2001) Listado de los coleópteros acuáticos (Insecta: Coleoptera) en ríos de la subcuenca Carcarañá (Córdoba, Argentina). Revista de la Sociedad Entomológica Argentina 60(1‒4): 183‒192.

Fabricius JC (1775) Systema entomologiae, sistens Insectorum classes, ordines, genera, species adiectis synonymis, locis, descriptionibus, observationibus. Flensburgi et Lipsiae: Libraria Korte, xxxii + 832 pp.

Fernández LA, Archangelsky M, Manzo V (2008) Coleópteros acuáticos del Parque Provincial Salto Encantado y Valle del Cuñá Pirú (Misiones, Argentina). Revista de la Sociedad Entomológica Argentina 67(3‒4): 87‒98.

Fernández LA, López Ruf ML (1999) Coleoptera y Heteroptera acuáticos y semiacuáticos de la Isla Martín García (Provincia de Buenos Aires). Physis, Sección B, 57(132‒133): 1‒4.

Fernández LA, López Ruf ML (2006) Aquatic Coleoptera and Heteroptera inhabiting waterbodies from Berisso, Buenos Aires province, Argentina. Revista de Biología Tropical 54(1): 139‒148.

Fernández LA, Torres PLM, Michat MC, Fischer S, Oliva A, Bachmann AO (2010) Coleópteros acuáticos y semiacuáticos del Parque Provincial Ernesto Tornquist (Provincia de Buenos Aires, Argentina). Revista de la Sociedad Entomológica Argentina 69(3‒4): 189‒199.

Ferreira-Jr N, Mendonça EC, Dorvillé LFM, Ribeiro JRI (1998) Levantamento preliminar e distribuição de besouros aquáticos (Coleoptera) na Restinga de Maricá, Maricá, RJ. In: Nessimian JL, Carvalho AL (Eds) Ecología de insetos Aquáticos. Series Oecologia Brasiliensis, vol. V, PPGE-UFRJ, Río de Janeiro, Brasil, 129‒140.

Fontanarrosa MS, Collantes MB, Bachmann AO (2009) Seasonal patterns of the insect community structure in urban rain pools of temperate Argentina. Journal of Insect Science 9(10): 1‒17.

Fontanarrosa MS, Torres PLM, Michat MC (2004) Comunidades de insectos acuáticos de charcos temporarios y lagunas en la ciudad de Buenos Aires (Argentina). Revista de la Sociedad Entomológica Argentina 63(3‒4): 55‒65.

García M (2018) *Prionohydrus plurunum*, nueva especie de coleóptero acuático (Coleoptera: Hydradephaga: Noteridae) del sudeste de Venezuela. Revista Chilena de Entomología 44(4): 419‒426.

García M (2020) Caracterización de nuevas especies del género *Suphisellus* Crotch (Coleoptera: Noteridae: Noterini), en Venezuela. Boletín del Centro de Investigaciones Biológicas 54(1): 41‒64.

García M, Jiménez-Ramos E (2020) Registros de insectos acuáticos (Arthropoda: Insecta) en dos hábitats *anpogeno* de Venezuela. Revista Chilena de Entomología 46(4): 725‒734. https://doi.org/10.35249/rche.46.4.20.18

García M, Navarro E (2001) Descripción de *Notaticus obscurus* (Coleoptera: Dytiscidae: Aubehydrini), nueva especie de escarabajo acuático en el oriente y nuevo registro para *Notaticus fasciatus* en el sur de Venezuela. Boletín del Centro de Investigaciones Biológicas 35(2): 141‒150.

Gómez RA, Miller KB (2013) *Prionohydrus*, a new genus of Noterini Thomson (Coleoptera: Noteridae) from South America with three new species and its phylogenetic considerations. Annals of the Entomological Society of America 106(1): 1‒12. <https://doi.org/10.1603/AN12041>

Gómez Lutz MC, Fernández LA, Kehr AI (2012) Coleópteros acuáticos de lagunas situadas en el noroeste de la provincia de Corrientes, Argentina. Revista de la Sociedad Entomológica Argentina 71(1‒2): 73‒85.

Gómez Lutz MC, Kehr AI (2017) A preliminary study of aquatic Coleoptera in temporary ponds and the ecological variables influencing their richness and diversity. Revista de la Sociedad Entomológica Argentina 76(3‒4): 7‒15. https://doi.org/10.25085/rsea.763402

Gómez Lutz MC, Kehr AI, Fernández LA (2015) Abundance, diversity and community characterization of aquatic Coleoptera in a rice field of Northeastern Argentina. Revista de Biología Tropical 63(3): 629‒638.

Grant S, Webbink K, Turcatel M, Shuman R (2025) Field Museum of Natural History (Zoology) Insect, Arachnid and Myriapod Collection. Version 12.63. Field Museum. Occurrence dataset https://doi.org/10.15468/0ywfpc accessed via GBIF.org on 2025-02-18.

Griffini A (1895) Viaggio del dottor Alfredo Borelli nella Repubblica Argentina e nel Paraguay. XI. Noto intorno ad alcuni ditiscidi. Bollettino dei Musei di Zoologia ed Anatomia Comparata della Reale Università di Torino 10(194): 1‒9.

Grosso LE (1979) Contribución al conocimiento biológico y sistemático de las especies argentinas de Noteridae (Coleoptera: Adephaga). PhD Thesis, Universidad de La Plata, Buenos Aires, Argentina.

Grosso LE (1993) Revisión de las especies neotropicales del género *Suphis* Aubé, con la descripción de *S. ticky* n. sp. (Coleoptera, Noteridae). Acta Zoológica Lilloana 42(2): 225‒238.

Guignot F (1942) Description d’un genre nouveau de Dytiscidae. Bulletin Mensuel de la Société Linnéenne de Lyon 11: 10‒13.

Guignot F (1950) Trente-troisième note sur les hydrocanthares. Bulletin de l’Institut Royal des Sciences Naturelles de Belgique 26(36): 1‒10.

Guignot F (1958) Contribution à la connaissance des dytiscides et gyrinides sud-américains (3ème série). Revue Française d’Entomologie 25: 33‒42.

Guimarães BAC, Ferreira-Jr N, Miller KB (2018) On *Hydrocanthus* Say, 1823 (Coleoptera: Noteridae): description of a new species, two new synonyms and a key to Brazilian species. Zootaxa 4508(2): 288‒300. https://doi.org/10.11646/zootaxa.4508.2.10

Hendrich L, Apenborn R, Burmeister E-G, Balke M (2015) A new species of *Agaporomorphus* Zimmermann, 1921 from Peru (Coleoptera, Dytiscidae, Copelatinae). ZooKeys 512: 63‒76. DOI: 10.3897/zookeys.512.9505

Horn GH (1894) The Coleoptera of Baja California. Proceedings of the California Academy of Sciences 4(2): 302‒449, pls 7‒8.

Laporte FLN Caumont de (1840) Histoire naturelle des insectes coléoptères. Tome premier. P Duménil, Paris, 324 pp. + 19 pls.

Leyva Escobar D, Megna YS, Deler-Hernández A (2014) Lista de los coleópteros acuáticos (Coleoptera: Adephaga, Polyphaga) de Jesús Menéndez, Las Tunas, Cuba. Novitates Caribaea (7): 105‒116.

Libonatti ML, Michat MC, Torres PLM (2011) Key to the subfamilies, tribes and genera of adult Dytiscidae of Argentina (Coleoptera: Adephaga). Revista de la Sociedad Entomológica Argentina 70(3–4): 317–336.

Libonatti ML, Michat MC, Torres PLM (2013) Aquatic Coleoptera from two protected areas of the Humid Chaco eco-region (Chaco Province, Argentina). Revista de la Sociedad Entomológica Argentina 72(3‒4): 155‒168.

Macchia GA (2022) Biological, ecological and faunistic aspects of the aquatic Coleoptera from Laguna de los Padres Integral Reserve and Paititi Private Natural Reserve (General Pueyrredón District, Buenos Aires Province) and its value in the local conservation. Historia Natural, Tercera Serie 12(3): 117‒132.

Macchia GA, Cicchino AC (2023) Nuevos aportes para el conocimiento de las especies de coleópteros acuáticos, semiacuáticos y anfibios del sistema serrano de Tandilia, Provincia de Buenos Aires. Historia Natural, Tercera Serie 13(2): 207‒234.

Macchia GA, Libonatti ML, Michat MC, Torres PLM (2015) Aquatic Coleoptera from El Cristal Natural Reserve (Santa Fe Province, Argentina). Revista de la Sociedad Entomológica Argentina 74(3‒4): 111‒116.

Manuel M (2015) Présence de *Derovatellus lentus* (Wehncke, 1876) en Guadeloupe (Coleoptera, Dytiscidae). Bulletin de la Société entomologique de France 120(4): 449‒450.

Manuel M (2017) Notes on the genus *Laccomimus*, with first record for Guadeloupe and clarification of the identity of *Laccophilus perparvulus* Régimbart, 1895 (Coleoptera, Dytiscidae). Bulletin de la Société entomologique de France 122(2): 215‒222.

Megna YS, Balke M, Apenborn R, Hendrich L (2019) A review of Peruvian diving beetles of the genus *Hydaticus* Leach, 1817, with description of *Hydaticus* (*Prodaticus*) *panguana* sp. nov. and notes on other Neotropical species (Coleoptera: Dytiscidae). Zootaxa 4615(1): 113‒130. <https://doi.org/10.11646/zootaxa.4615.1.5>

Megna YS, Deler A (2006) Composición taxonómica, distribución y bionomía de la familia Noteridae (Coleoptera: Adephaga) en Cuba. Revista de la Sociedad Entomológica Argentina 65(1‒2): 69‒80.

Megna YS, Sánchez-Fernández D (2016) Catálogo faunístico de la familia Dytiscidae (Coleoptera: Adephaga) en Cuba. Boletín de la Sociedad Entomológica Aragonesa 59: 159‒180.

Michat MC (2006) Descriptions of larvae of *Megadytes* (Coleoptera: Dytiscidae: Dytiscinae): The hypothesis of monophyletic origin revisited. European Journal of Entomology 103: 831‒842. DOI: 10.14411/eje.2006.114

Michat MC (2008) Description of the larvae of three species of *Laccophilus* Leach and comments on the phylogenetic relationships of the Laccophilinae (Coleoptera: Dytiscidae). Zootaxa 1922: 47‒61. DOI: 10.5281/zenodo.184741

Michat MC (2010) Descriptions of larvae of *Megadytes* (Coleoptera: Dytiscidae: Dytiscinae): The subgenera *Trifurcitus* and *Megadytes* s. str., ground plan of chaetotaxy of the genus and phylogenetic analysis. European Journal of Entomology 107: 377‒392. DOI 10.14411/eje.2010.047

Michat MC, Alarie Y (2009) Phylogenetic relationships of *Notaticus* (Coleoptera: Dytiscidae) based on larval morphology. Annals of the Entomological Society of America 102(5): 797–808. DOI [10.1603/008.102.0506](https://doi.org/10.1603/008.102.0506)

Michat MC, Alarie Y, Torres PLM, Megna YS (2007) Larval morphology of the diving beetle *Celina* and the phylogeny of ancestral hydroporines (Coleoptera: Dytiscidae: Hydroporinae). Invertebrate Systematics 21: 239‒254. DOI 10.1071/IS06037

Michat MC, Alarie Y, Benetti CJ (2022) Larval morphology of the Neotropical diving beetle genus *Hemibidessus* Zimmermann, 1921 (Coleoptera: Dytiscidae: Bidessini). Zootaxa 5168(3): 388–400. DOI 10.11646/zootaxa.5168.3.9.

Michat MC, Alarie Y, Benetti CJ (2024) Description of the mature larva of *Derovatellus lentus* (Coleoptera: Dytiscidae). Zootaxa 5492(1): 145–150. DOI 10.11646/zootaxa.5492.1.10.

Michat MC, Alarie Y, Benetti CJ, Urcola JI, Rodriguez G, Torres PLM (2025) Larval morphology of the enigmatic genus *Queda* Sharp, 1882 supports monophyly of Hydrovatini (Coleoptera, Dytiscidae). Arthropod Systematics & Phylogeny 83: 303–314. DOI 10.3897/asp.83.e150736

Michat MC, Archangelsky M (2007) Descriptions of larvae of *Desmopachria* Babington (Coleoptera: Dytiscidae: Hydroporinae): the *D. vicina* Sharp species group. The Coleopterists Bulletin 61(2): 264–276.

Michat MC, Archangelsky M (2014) Coleoptera, Gyrinidae. In: Roig-Juñent S, Claps LE, Morrone JJ (Eds) Biodiversidad de artrópodos argentinos, Vol. 3. Editorial INSUE/Universidad Nacional de Tucumán, San Miguel de Tucumán, 455–465.

Michat MC, Torres PLM (2005) Larval morphology of *Macrovatellus haagi* (Wehncke) and phylogeny of Hydroporinae (Coleoptera: Dytiscidae). Insect Systematics & Evolution 36(2): 199–217. DOI [10.1163/187631205788838456](https://doi.org/10.1163/187631205788838456)

Michat MC, Torres PLM (2008) On the systematic position of the diving-beetle genus *Pachydrus* (Coleoptera: Dytiscidae: Hydroporinae): evidence from larval chaetotaxy and morphology. European Journal of Entomology 105(4): 737–750. DOI [10.14411/eje.2008.100](https://doi.org/10.14411/eje.2008.100)

Michat MC, Torres PLM (2009) A preliminary study on the phylogenetic relationships of *Copelatus* Erichson (Coleoptera: Dytiscidae: Copelatinae) based on larval chaetotaxy and morphology. Hydrobiologia 632(1): 309–327. https://doi.org/10.1007/s10750-009-9853-2

Michat MC, Torres PLM (2011) Phylogenetic relationships of the tribe Vatellini based on larval morphology, with description of *Derovatellus lentus* (Coleoptera: Dytiscidae: Hydroporinae). Annals of the Entomological Society of America 104(5): 863‒877. DOI 10.1603/AN11054

Michat MC, Torres PLM (2013) The unknown larva of the minute diving beetle genus *Brachyvatus* Zimmermann, 1919 (Coleoptera: Dytiscidae: Bidessini). Entomological News 123(2):139‒153. http://dx.doi.org/10.3157/021.123.0205

Miller KB (2001) Revision of the Genus *Agaporomorphus* Zimmermann (Coleoptera: Dytiscidae). Annals of the Entomological Society of America 94(4): 520‒529. [https://doi.org/10.1603/0013-8746(2001)094[0520:ROTGAZ]2.0.CO;2](https://doi.org/10.1603/0013-8746(2001)094%5b0520:ROTGAZ%5d2.0.CO;2)

Miller KB (2002) Revision of the Neotropical genus *Hemibidessus* Zimmermann (Coleoptera: Dytiscidae: Hydroporinae: Bidessini). Aquatic Insects 23(4) (2001): 253‒275. http://dx.doi.org/10.1076/aqin.23.4.253.4876

Miller KB (2005) Revision of the New World and south-east Asian Vatellini (Coleoptera: Dytiscidae: Hydroporinae) and phylogenetic analysis of the tribe. Zoological Journal of the Linnean Society 144: 415‒510. <https://doi.org/10.1111/j.1096-3642.2005.00180.x>

Miller KB (2016) New species of *Bidessonotus* Régimbart, 1895 with a review of the South American species (Coleoptera, Adephaga, Dytiscidae, Hydroporinae, Bidessini). ZooKeys 622: 95‒127. https://doi.org/10.3897/zookeys.622.9155

Miller KB (2017) A review of the Neotropical genus *Bidessodes* Régimbart, 1895 including description of four new species (Coleoptera, Adephaga, Dytiscidae, Hydroporinae, Bidessini). ZooKeys 658: 9‒38. https://doi.org/10.3897/zookeys.658.10928

Miller KB, Bergsten J, Whiting MF (2007) Phylogeny and classification of diving beetles in the tribe Cybistrini (Coleoptera, Dytiscidae, Dytiscinae). Zoologica Scripta 36(1): 41‒59. doi:10.1111/j.1463-6409.2006.00254.x

Miller KB, Bergsten J, Whiting MF (2009) Phylogeny and classification of the tribe Hydaticini (Coleoptera: Dytiscidae): partition choice for Bayesian analysis with multiple nuclear and mitochondrial protein-coding genes. Zoologica Scripta 38(6): 591‒615. doi:10.1111/j.1463-6409.2009.00393.x

Moroni JC (1988) Revisión del género *Rhantus* Dejean en Chile (Coleoptera: Dytiscidae: Colymbetinae). Revista Chilena de Entomología 16: 49‒64.

Mouchamps R (1955) Note systématique sur le genre *Suphis* Aubé. Bulletin de l’Institut Royal des Sciences Naturelles de Belgique 31(57): 1‒7.

Mouchamps R (1957) Contribution à la connaissance des Cybisterini (Col. Dytiscidae) du Musée d’Histoire Naturelle de Vienne (9e note). Annalen des Naturhistorischen Museum in Wien 61: 278‒287.

Nilsson AN (2011) A World Catalogue of the Family Noteridae, or the Burrowing Water Beetles (Coleoptera, Adephaga). Version 16.VIII.2011. <http://www.waterbeetles.eu/documents/W_CAT_Noteridae.pdf>

Nilsson AN, Hájek J (2025) A World Catalogue of the Family Dytiscidae, or the Diving Beetles (Coleoptera, Adephaga). Version 1.I.2025. https://www.waterbeetles.eu/documents/W_CAT_Dytiscidae_2025.pdf

Ochs G (1956) Uber die Gyriniden von Uruguay. Comunicaciones Zoológicas del Museo de Historia Natural de Montevideo 4(76): 1‒13.

Olivier AG (1791‒1792) Encyclopédie méthodique, ou par ordre de matières. Histoire naturelle. Insectes. Tome sixième. Paris: C-J Panckoucke, 704 pp. [pp. 1‒368 (livraison 47) published 21 November 1791, pp. 369‒704 (livraison 51) published 01 October 1792]

Pederzani F, Rocchi S (2012) A new species of *Neobidessus* Young from Argentina (Insecta, Coleoptera, Dytiscidae). Quaderno di Studi e Notizie di Storia Naturale della Romagna 35: 109‒113.

Philippi RA, Philippi FHE (1860) Coleoptera nonnulla nova Chilensia praesertim Valdiviana. Entomologische Zeitung 21: 245‒251.

Régimbart M (1883) Essai monographique de la famille des Gyrinidae. 2e partie. Annales de la Société entomologique de France 52: 121–190 + 5 pl.

Régimbart M (1889a) Énumération des Haliplidae, Dytiscidae et Gyrinidae recueillis par Mr. le prof. L. Balzan dans l’Amérique méridionale et description de quelques autres espèces voisines. Annali del Museo Civico di Storia Naturale, Genova (2)7: 256‒268.

Régimbart M (1889b) Voyage de M.E. Simon au Venezuela (décembre 1887-avril 1888). Dytiscidae et Gyrinidae. Annales de la Société Entomologique de France (6) 8 (1888): 379‒387.

Régimbart M (1889c) Dytiscidae et Gyrinidae nouveaux ou rares de la collection du Musée Royal de Leyde. Notes from the Leyden Museum 11: 51‒63.

Régimbart M (1895) Dytiscides trouvés dans les tabacs par les soins de M. Antoine Grouvelle. Annales de la Société Entomologique de France 64: 321‒348 + pl. 8: figs 3‒18.

Régimbart M (1899a) Viaggio del Dott. Alfredo Borelli nel Chaco boliviano en ella Repubblica Argentina. XV. Dytiscidae de la Bolivie. Bolletino dei Musei di Zoologia ed Anatomia comparata della Reale Università di Torino 14(340): 1.

Régimbart M (1899b) Dytiscidae et Gyrinidae nouveaux du Musée Civique de Gênes. Annali del Museo Civico di Storia Naturale, Genova (2) 20: 193‒198.

Régimbart M (1903) Liste des Dytiscidae & Gyrinidae recueillis par le Dr Philippe Silvestri dans l’Amerique méridionale de 1898 à 1900. Bolletino della Società Entomologica Italiana 35: 46‒74.

Régimbart M (1904) Dytiscides et gyrinides recueillis au Vénézuéla et à la Guyane par M.F. Geay et faisant partie des collections du Muséum d’Histoire Naturelle. Bulletin du Muséum National d’Histoire Naturelle Paris 1904(5): 224‒226.

Roback SS, Berner L, Flint Jr OS, Nieser N, Spangler PJ (1980) Results of the Catherwood Bolivian-Peruvian Altiplano Expedition Part. I Aquatic Insects except Diptera. Proceedings of the Academy of Natural Sciences of Philadelphia 132: 176‒217.

Sahlberg RF (1844) Coleoptera diebus XV-XXVII decembris anni MDCCCXXXIX ad Rio Janeiro lecta. Part 1. Acta Societatis Scientiarum Fenniae 2(1): 499‒522.

Scheers K, Thomaes A (2017) A review of the burrowing water beetles of Belize with a key to the species (Coleoptera: Noteridae). Belgian Journal of Entomology 51: 1‒17.

Sharp D (1882a) On aquatic carnivorous Coleoptera or Dytiscidae. The Scientific Transactions of the Royal Dublin Society, Series II 2: 179‒1003 + pls 7‒18.

Sharp D (1882b) Biologia Centrali-Americana. Zoologia. Insecta. Coleoptera. Vol. 1. Part 2. London: Published for the editors by R. H. Porter, xvi + 144 pp. + 4 pls.

Spangler PJ (1973) The nomenclature, bionomics, and distribution of *Notaticus fasciatus* (Coleoptera: Dytiscidae: Aubehydrinae). Proceedings of the Biological Society of Washington 86(42): 495‒500.

Steinheil EW (1869) Symbolae ad historiam Coleopterorum Argentiniae meridionalis, ossia elenco dei coleotteri raccolti dal professore Pellegrino Strobel. Atti della Società Italiana di Scienze Naturali, Milano 12: 238‒260.

Toledo M, Michat MC (2015) Description of *Laccomimus* gen. n. and eleven new species from the Neotropical region (Coleoptera, Dytiscidae, Laccophilinae). Zootaxa 3990(3): 301‒354. http://dx.doi.org/10.11646/zootaxa.3990.3.1

Torres PLM, Mazzucconi SA, Michat MC (2007) Los coleópteros y heterópteros acuáticos del Parque Nacional El Palmar (Provincia de Entre Ríos, Argentina): lista faunística, diversidad y distribución. Revista de la Sociedad Entomológica Argentina 66(3‒4): 127‒154.

Torres PLM, Mazzucconi SA, Michat MC, Bachmann (2008) Los coleópteros y heterópteros acuáticos del Parque Nacional Calilegua (Provincia de Jujuy, Argentina). Revista de la Sociedad Entomológica Argentina 67(1‒2): 127‒144.

Torres PLM, Michat MC, Libonatti ML, Fernández LA, Oliva A, Bachmann AO (2012) Aquatic Coleoptera from Mburucuyá National Park (Corrientes Province, Argentina). Revista de la Sociedad Entomológica Argentina 71(1‒2): 57‒71.

Trémouilles ER (1984) El género *Rhantus* Dejean en la Argentina (Coleoptera, Dytiscidae). Physis, Sección B, 42(102): 9‒24.

Trémouilles ER (1989a) Notas sobre Coleoptera acuáticos neotropicales. III. Datos ampliatorios sobre distribución geográfica de especies de *Megadytes* Sharp (Coleoptera: Dytiscidae). Revista de la Sociedad Entomológica Argentina 45(1‒4): 159‒161.

Trémouilles ER (1989b) Contribución para el conocimiento del género *Thermonectus* Dejean en la Argentina y áreas limítrofes (Coleoptera, Dytiscidae). Revista de la Sociedad Entomológica Argentina 46(1‒4): 95‒115.

Trémouilles ER (1996) Revisión del género *Hydaticus* Leach en América del Sur, con descripción de tres nuevas especies (Coleoptera, Dytiscidae). Physis, Sección B, 52(122‒123): 15‒32.

Trémouilles ER (1998) Dytiscidae. In: Morrone JJ, Coscarón S (Eds) Biodiversidad de artrópodos argentinos. Una perspectiva biotaxonómica. Ediciones Sur, La Plata, 210‒217.

Trémouilles ER, Bachmann AO (1980) La tribu Cybisterini en la Argentina (Coleoptera, Dytiscidae). Revista de la Sociedad Entomológica Argentina 39(1‒2): 101‒125.

Trémouilles ER, Bachmann AO (1981) Distribución del género *Notaticus* en la Argentina (Coleoptera, Dytiscidae). Physis, Sección B, 39(97): 101‒102.

Trémouilles ER, Michat MC, Torres PLM (2005) A synopsis of the South American *Hydrovatus* (Coleoptera: Dytiscidae: Hydroporinnae), with notes on habitat and distribution, and a key to species. Revista de la Sociedad Entomológica Argentina 64(1‒2): 61‒69.

Trémouilles ER, Torres PLM, Michat MC (2004) New distribucional records and comments for the species of the genus *Queda* (Coleoptera: Dytiscidae). Revista de la Sociedad Entomológica Argentina 63(1‒2): 38‒40.

Urcola JI, Alarie Y, Benetti CJ, Michat M (2019c) Larval morphology of *Suphisellus* Crotch, 1873 (Coleoptera: Noteridae): description of first instar of *S. rufipes* (Sharp, 1882) with biological notes and chaetotaxy analysis. Annales Zoologici 69(4): 817‒825. <https://doi.org/10.3161/00034541ANZ2019.69.4.013>

Urcola JI, Alarie Y, Benetti CJ, Rodriguez G, Michat MC (2019b) Larval morphology and analysis of primary chaetotaxy in the genus *Suphis* Aubé, 1836 (Coleoptera: Noteridae). Zootaxa 4619(1): 121‒138. DOI 10.11646/zootaxa.4619.1.5.

Urcola JI, Alarie Y, Benetti CJ, Torres PLM, Michat MC (2022) Description and chaetotaxic analysis of the larval instars of *Hydrocanthus debilis* Sharp, 1882 (Coleoptera: Noteridae). Zootaxa 5196(1): 135‒144. <https://doi.org/10.11646/zootaxa.5196.1.7>

Urcola JI, Baca SM, Rodriguez G, Michat MC (2024b) *Prionohydrus cambyreta* sp. nov. from Iberá wetlands—the first species of the genus from northern Argentina (Coleoptera: Noteridae). Zootaxa 5523(4): 483‒493. https://doi.org/10.11646/zootaxa.5523.4.7

Urcola JI, Benetti CJ, Alarie Y Torres PLM, Michat MC (2019a) Morphology and chaetotaxy of the instars of *Hydrocanthus sharpi* Zimmermann, 1928 (Coleoptera: Noteridae). The Coleopterists Bulletin 73(3): 611‒620.

Urcola JI, Benetti CJ, Alarie Y, Urcola MR, Michat MC (2024a) Description of the first- and third-instar larvae of *Hydrocanthus levigatus* (Brullé, 1837) (Coleoptera: Noteridae). Aquatic Insects 45(2): 260‒272. https://doi.org/10.1080/01650424.2023.2245802

Urcola JI, Benetti CJ, Baca SM, Michat MC (2020) *Suphisellus grossoi* sp. n., a new burrowing water beetle from South America, and notes on *S. flavolineatus* (Régimbart, 1889) and *S. grammicus* (Sharp, 1882) (Coleoptera: Noteridae). Zootaxa 4786(1): 122‒130. https://doi.org/10.11646/zootaxa.4786.1.9

Urcola JI, Michat MC (2023a) Description of the mature larva of *Suphisellus curtus* (Sharp, 1882) (Coleoptera: Noteridae) with chaetotaxy analysis. Aquatic Insects 44(4): 273‒283. https://doi.org/10.1080/01650424.2022.2162086

Urcola JI, Michat MC (2023b) Coleoptera, Noteridae. In: Claps LE, Roig-Juñent S, Morrone JJ (Eds) Biodiversidad de artrópodos argentinos, Vol. 5. Editorial INSUE/Universidad Nacional de Tucumán, San Miguel de Tucumán, 190–197.

van Vondel BJ (2016) Corrections and additions to the “Revision of the Haliplidae of the Neotropical Region including Mexico” (Vondel and Spangler 2008) with description of a new species (Coleoptera: Haliplidae). Koleopterologische Rundschau 86: 51‒59.

van Vondel BJ, Spangler PJ (2008) Revision of the Haliplidae of the Neotropical Region including Mexico (Coleoptera: Haliplidae). Koleopterologische Rundschau 78: 69‒194.

Viana MJ (1937) Lista de insectos de la Isla Martín García. I. Coleoptera. Revista de la Sociedad Entomológica Argentina 9: 101‒109.

Viana MJ, Williner GJ (1972) Evaluación de la fauna entomológica y aracnológica de las provincias cuyanas. Primera comunicación. Acta Scientifica, Serie Entomología 5: 1‒29.

Viana MJ, Williner GJ (1978) Evaluación de la fauna entomológica y aracnológica de las provincias centrales y cuyanas (cuarta comunicación). Acta Scientifica, Serie Entomología 11: 1‒77.

Vidal Sarmiento J, Grosso LE (1971) Notas sobre halíplidos argentinos (Coleoptera). II. Revisión de las especies argentinas. Revista de la Sociedad Entomológica Argentina 33(1‒4): 147‒157.

von Ellenrieder N, Fernández LA (2000) Aquatic Coleoptera in the Subtropical-Pampasic Ecotone (Argentina, Buenos Aires): species composition and temporal changes. The Coleopterists Bulletin 54(1): 23‒35. DOI: 10.1649/0010-065X(2000)054[0023:ACITSP]2.0.CO;2

Wehncke E (1876) Neue Dytisciden. Stettiner Entomologische Zeitung 37: 356‒360.

World Conservation Monitoring Centre (1996) *Rhantus orbignyi*. The IUCN Red List of Threatened Species 1996: e.T19461A8894689. http://dx.doi.org/10.2305/IUCN.UK.1996.RLTS.T19461A8894689.en

Young FN (1979) Water beetles of the genus *Suphisellus* Crotch in the Americas north of Colombia (Coleoptera: Noteridae). The Southwestern Naturalist 24(3): 409‒429.

Young FN (1981) Predaceous water beetles of the genus *Neobidessus* from South America (Coleoptera: Dytiscidae). The Coleopterists Bulletin 35(3): 317‒340.

Young FN (1985) A key to the American species of *Hydrocanthus* Say, with descriptions of new taxa (Coleoptera: Noteridae). Proceedings of the Academy of Natural Sciences of Philadelphia 137: 90‒98.

Young FN (1986) Review of the predaceous water beetles of the genus *Bidessodes* Régimbart (Coleoptera, Dytiscidae). Entomologica Basiliensia 11: 203‒220.

Zimmermann A (1919) Die Schwimmkäfer des Deutschen Entomologischen Museums in Berlin-Dahlem. Archiv für Naturgeschichte, Abteilung A 83(12) (1917): 68‒249.

Zimmermann A (1921) Beiträge zur Kenntnis der südamerikanischen Schwimmkäferfauna nebst 41 Neubeschreibungen. Archiv für Naturgeschichte 87A(3): 181‒206.

Zimmermann A (1924) Wissenschaftliche Ergebnisse der schwedischen entomologischen Reise des Herrn Dr. A. Roman in Amazonas 1914‒1915. 9. Dytiscidae und Gyrinidae. Arkiv för Zoologi 16(4): 1‒3.

Zimmermann A (1925) Drei neue Dytisciden aus Sao Paulo (Brasilien). (Dytisc.). Entomologische Mitteilungen 14(3‒4): 254‒256.

Zimmermann A (1928) Neuer Beitrag zur Kenntnis der Schwimmkäfer. Wiener Entomologische Zeitung 44: 165‒187.
